# Effect of lifestyle interventions on cardiovascular risk factors among adults without impaired glucose tolerance or diabetes: A systematic review and meta-analysis

**DOI:** 10.1371/journal.pone.0176436

**Published:** 2017-05-11

**Authors:** Xuanping Zhang, Heather M. Devlin, Bryce Smith, Giuseppina Imperatore, William Thomas, Felipe Lobelo, Mohammed K. Ali, Keri Norris, Stephanie Gruss, Barbara Bardenheier, Pyone Cho, Isabel Garcia de Quevedo, Uma Mudaliar, Christopher D. Jones, Jeffrey M. Durthaler, Jinan Saaddine, Linda S. Geiss, Edward W. Gregg

**Affiliations:** 1 Division of Diabetes Translation, National Centers for Chronic Disease Prevention and Health Promotion, Centers for Disease Control and Prevention, Atlanta, Georgia, United States of America; 2 Office of Public Health Scientific Services, Centers for Disease Control and Prevention, Atlanta, Georgia, United States of America; 3 Hubert Department of Global Health, Rollins School of Public Health, Emory University, Atlanta, Georgia, United States of America; 4 Health Policy and Administration, Fulton-DeKalb Hospital Authority, Atlanta, Georgia, United States of America; 5 Office on Smoking and Health, National Center for Chronic Disease Prevention and Health Promotion, Centers for Disease Control and Prevention, Atlanta, Georgia, United States of America; 6 Division for Heart Disease and Stroke Prevention, National Center for Chronic Disease Prevention and Health Promotion, Centers for Disease Control and Prevention, Atlanta, Georgia, United States of America; Florida International University Herbert Wertheim College of Medicine, UNITED STATES

## Abstract

Structured lifestyle interventions can reduce diabetes incidence and cardiovascular disease (CVD) risk among persons with impaired glucose tolerance (IGT), but it is unclear whether they should be implemented among persons without IGT. We conducted a systematic review and meta-analyses to assess the effectiveness of lifestyle interventions on CVD risk among adults without IGT or diabetes. We systematically searched MEDLINE, EMBASE, CINAHL, Web of Science, the Cochrane Library, and PsychInfo databases, from inception to May 4, 2016. We selected randomized controlled trials of lifestyle interventions, involving physical activity (PA), dietary (D), or combined strategies (PA+D) with follow-up duration ≥12 months. We excluded all studies that included individuals with IGT, confirmed by 2-hours oral glucose tolerance test (75g), but included all other studies recruiting populations with different glycemic levels. We stratified studies by baseline glycemic levels: (1) low-range group with mean fasting plasma glucose (FPG) <5.5mmol/L or glycated hemoglobin (A1C) <5.5%, and (2) high-range group with FPG ≥5.5mmol/L or A1C ≥5.5%, and synthesized data using random-effects models. Primary outcomes in this review included systolic blood pressure (SBP), diastolic blood pressure (DBP), total cholesterol (TC), low density lipoprotein cholesterol (LDL-C), high density lipoprotein cholesterol (HDL-C), and triglycerides (TG). Totally 79 studies met inclusion criteria. Compared to usual care (UC), lifestyle interventions achieved significant improvements in SBP (-2.16mmHg[95%CI, -2.93, -1.39]), DBP (-1.83mmHg[-2.34, -1.31]), TC (-0.10mmol/L[-0.15, -0.05]), LDL-C (-0.09mmol/L[-0.13, -0.04]), HDL-C (0.03mmol/L[0.01, 0.04]), and TG (-0.08mmol/L[-0.14, -0.03]). Similar effects were observed among both low-and high-range study groups except for TC and TG. Similar effects also appeared in SBP and DBP categories regardless of follow-up duration. PA+D interventions had larger improvement effects on CVD risk factors than PA alone interventions. In adults without IGT or diabetes, lifestyle interventions resulted in significant improvements in SBP, DBP, TC, LDL-C, HDL-C, and TG, and might further reduce CVD risk.

## Introduction

Cardiovascular disease (CVD) is the number one killer globally.[1] CVD is also the major cause of morbidity and mortality among persons with diabetes, and the largest contributor to health care costs associated with diabetes.[2,3] On the other hand, CVD and diabetes share similar risk factors such as unhealthy diet, physical inactivity, and obesity.[2–4] Previous studies have demonstrated that structured lifestyle interventions incorporating physical activity, diet, and behavior change strategies could prevent or delay type 2 diabetes incidence and reduce CVD risk factors.[5–7] However, these major prevention trials focused on populations with impaired glucose tolerance (IGT).[5–7] Although individuals with IGT are the priority target population because they lie at the higher end of the diabetes risk spectrum, populations without IGT but with other CVD risk factors may outnumber those with high diabetes risk and have the same urgent needs for risk reduction, as many RCT studies have indicated.[8–14] According to the American Diabetes Association’s (ADA) definitions of pre-diabetes (which includes impaired fasting glucose (IFG): 100-125mg/dL), about 60% of US individuals with pre-diabetes do not have IGT,[15] and according to the World Health Organization’s (WHO) definition of intermediate hyperglycemia (measured by fasting plasma glucose (FPG): 110-139mg/dL), about 70% of individuals with this condition do not have IGT.[16] Whether lifestyle interventions should be applied more broadly to the population at lower risk (i.e. those below the IGT threshold) to reduce CVD risk needs to be examined.

According to an American Heart Association (AHA) Special Report,[17] cardiovascular health is defined by 7 metrics, including health behaviors and health indicators as follows: smoking status, body mass index (BMI), physical activity (PA) levels, healthy diet scores, total cholesterol (TC), blood pressure (BP) level, and fasting plasma glucose level. To achieve the AHA ideal cardiovascular health promotion goal, each indicator must fall into certain ranges (e.g., FPG<100 mg/dL). This definition of cardiovascular health addresses health behaviors and health indicators related to both CVD and diabetes, and thus offer guidance for how to achieve improvements in preventing both CVD and diabetes at the same time.

Evidence regarding the effects of lifestyle intervention on CVD risk reduction has previously been systematically synthesized by examining 6 of the 7 CVD health indicators mentioned above, especially by examining the different stratum of BMI (e.g., moderate weight loss will reduce both diabetes and CVD risk among overweight or obese populations[5–7]), as indicated by the 2013 AHA/ACC Guideline on Lifestyle Management to Reduce Cardiovascular Risk.[18] However, how this evidence is aligned with the stratification of different glucose levels is still unclear. Lack of this information may prevent public health practitioners from fully understanding the role lifestyle interventions can play in reducing both diabetes and CVD risk among populations with varying risk levels. In contrast, a synthesis of evidence on the impact of lifestyle interventions among populations with different risk levels may help to inform decisions regarding the allocation of finite public health resources.

We conducted a systematic review to assess the aggregated impact of lifestyle interventions on glucose regulation and CVD risk factors among adults (age≥18 years) without IGT or diabetes. By conducting this review, we intend to answer the following research question: can lifestyle interventions similar to those found efficacious among populations with IGT achieve the same magnitude of improvement in CVD risk reduction among populations with lower diabetes risk? We also aimed to examine whether lifestyle interventions focused on diet, PA or their combination have varying impact on CVD risk reduction. To understand how to reach the comprehensive goal of preventing both CVD and diabetes, we also examined how the lifestyle interventional effect on CVD risk reduction is related to the effect sizes of glucose improvement and weight loss.

## Materials and methods

### Search strategy and selection criteria

We followed Cochrane Collaboration standards for a meta-analysis of randomized control trial (RCT) studies to develop our protocol.[19] We systemically searched MEDLINE, EMBASE, CINAHL, Web of Science, the Cochrane Library, and PsychInfo databases, from inception to May 4, 2016. Medical Subject Headings, text words, and search strategies are presented in our online-only supplements (S1 File). We examined reference lists of all included studies and relevant reviews for additional studies. We directly contacted authors to clarify data as needed.

We selected RCTs published in any language that examined lifestyle strategies involving PA and/or dietary (D) interventions, among adults (≥18 years) and with glycemic indicators and CVD risk factors reported as intervention outcomes (e.g., systolic blood pressure (SBP), diastolic blood pressure (DBP), TC, low density lipoprotein cholesterol (LDL-C), high density lipoprotein cholesterol (HDL-C), or triglycerides (TG)). Included studies investigated persons without IGT or diabetes. We excluded all studies that included individuals with IGT, confirmed by 2-hours oral glucose tolerance test (75g), but included all other studies recruiting populations with different glycemic levels. However, to examine whether there was heterogeneity of effect by baseline glycemia, we grouped all studies as: (1) low range glycemia group with mean fasting plasma glucose (FPG)<5.5mmol/L or mean glycated hemoglobin (A1C)<5.5% and (2) high range group with mean FPG≥5.5mmol/L or mean A1C≥5.5%. Data from the low and high range glycemic groups were analyzed separately. We only included interventions with a follow-up interval of at least 12 months.

### Study selection and data extraction

Two reviewers independently reviewed each article title and abstract for inclusion. If any disagreement occurred between two reviewers, a third reviewed the item and consensus was reached through discussions.

We extracted data regarding demographic and intervention characteristics. Primary outcomes included SBP, DBP, TC, LDL-C, HDL-C, and TG. In our review, all interventions were classified as PA alone, D alone, or combined interventions (PA+D). PA interventions included any strategy used to promote physical activity levels using counseling, exercise prescription, and/or a supervised or unsupervised exercise program. D interventions included any strategy used to reduce or control calorie intake, e.g., very low-calorie diet (<800 kcal/d) or low-calorie diet (800 to 1500 kcal/d). Studies using combined PA and D strategies usually also employed behavioral modification strategies, including counseling, education, cognitive-behavioral therapy, or social support, as an intervention component.

### Statistical analysis and quality assessment

We assessed study quality by examining potential selection, attrition, and detection bias.[19] We did not exclude any study that was considered poor quality (e.g., studies with attrition ≥30%). However, we conducted a sensitivity analysis to compare pooled effects between studies with potentially significant bias and those without. For example, for those studies with attrition ≥30%, their data were not used in our primary meta-analyses, but were used in our sensitivity analyses.

Among studies with similar intervention and comparison groups reporting a similar outcome of interest, we conducted meta-analyses to determine pooled effects. We calculated the mean difference between baseline and follow-up measures for the intervention (I) and comparison (C) groups (delta I and delta C) and the standard error of each difference. We used three strategies to estimate pooled effects: (1) stratified by baseline glucose levels (low range vs. high range); (2) stratified by the length of follow-up (12months vs. 13–23 months vs. ≥24 months); and (3) stratified by type of interventions (PA vs. D vs. PA+D).

We used DerSimonian and Laird random-effects models[20] to determine pooled effects. Effect size was defined by the mean difference between delta I and delta C divided by the standard deviation of the mean. We used meta-regression to determine whether various study-level characteristics (mean age, follow-up interval, duration of the intervention, number of intervention contacts, attrition, and year of publication) affected the between-group differences in SBP, DBP, TC, LDL-C, HDL-C, and TG, and we examined interaction terms for all models. We also used meta-regression analyses to examine the relationship between interventional effects on CVD risk reduction and interventional effects on diabetes risk reduction measured by the effect sizes of glucose improvement and weight loss. The meta-regression was conducted using SPSS (version 20.0, Armonk, NY: IBM Corp.). We used the chi-squared test to examine heterogeneity, and we used Cochrane Review Manager software (version 5.1; Copenhagen, Denmark) to calculate pooled effects.

If a comparison group in a study used a similar approach as the intervention group did, but only differed in dose, intensity, or frequency (e.g., diet plan A vs. diet plan B; or swimming vs. walking), we analyzed the effects of treatment in a single arm model to determine within-group changes (between post-intervention and pre-intervention in one arm) for both intervention and comparison group. These effects were also estimated by using the DerSimonian and Laird random-effect model. We did not, however, conduct any sensitivity analysis for these studies. Because this paper focused on the net lifestyle intervention effect (any lifestyle intervention vs. no intervention [e.g., usual care (UC)]), pooled effects from our single arm model are not reported in our results section, but are presented as an online supplementary table (Table C in S1 File).

## Results

**Fig 1:**
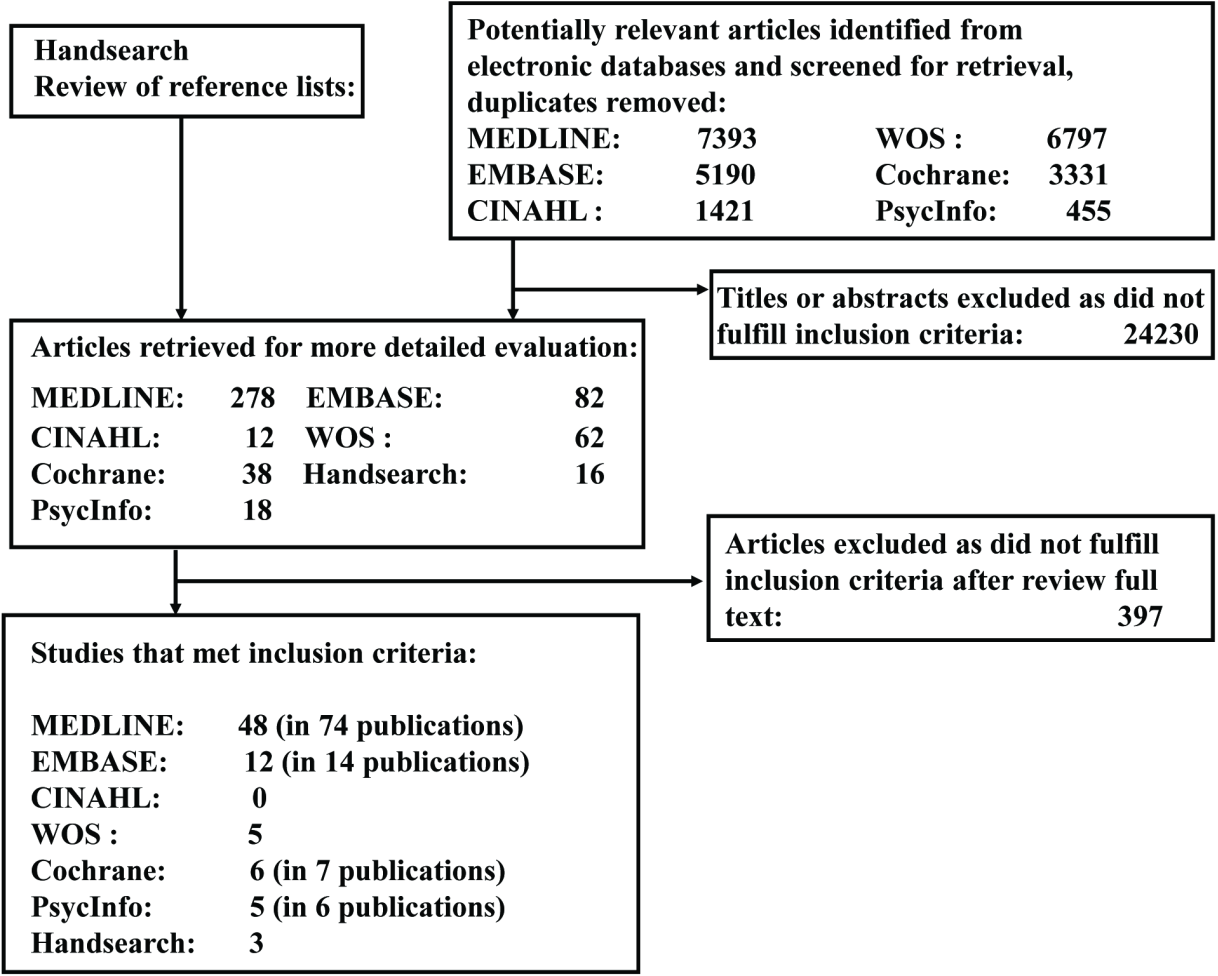
Study flow diagram. CINAHL, Cumulative Index to Nursing and Allied Health Literature EMBASE, Excerpta Medica Database MEDLINE, Medical Literature Analysis and Retrieval System Online PsycInfo, Psychological Information Database WOS, Web of Science.

Seventy-nine studies[10,11,13,14, 21–95] and 30 companion publications[9,96–124] encompassing 15618 participants (Table 1: range, 20 to 1089) fulfilled the inclusion criteria (Fig 1). Follow-up time ranged from 12 to 54 months. The mean age of the participants was 50.6 years (range, 30.2 to 70.4 year), and mean BMI was 30.5 kg/m^2^ (range, 23.3 to 38.7 kg/m^2^). Mean baseline SBP, DBP, TC, LDL-C, HDL-C, and TG were 127.5 mmHg, 79.2 mmHg, 5.4 mmol/L, 3.3 mmol/L. 1.3 mmol/L, and 1.5 mmol/L, respectively. More studies took place in community settings than in clinics (58 vs. 21). Sampling methods varied, but most participants were recruited through screening programs. Attrition ranged from 0% to 60%, and in 16 studies,[21,34–36,45,60,62,66,69,74,76,78,81,82,86,94] attrition was 30% or more; longer follow-up resulted in higher attrition. Thirty-nine studies with mean FPG <5.5mmol/L or mean A1C <5.5% were classified as low range group, and 40 studies with mean FPG ≥5.5mmol/L or mean A1C ≥5.5% were classified as high range group.

**Table 1:** Characteristics of study participants.

Citation	Sample size	Length of follow-up (month)	Age at BL (years) [mean (SD)]	Sex (% female)	Setting; Race/ethnicity	BMI at BL (kg/m^2^) [mean (SD)]	SBP/DBP at BL (mmHg) [mean (SD)]	TC at BL (mmol/L) [mean (SD)]	LDL/HDL at BL (mmol/L) [mean (SD)]	TG at BL (mmol/L) [mean (SD)]	Inclusion criteria	Sampling method	Attrition (%)
Ackermann et al. 2008	92	12	58.3 (10.1)	55.4	Community Indianapolis IN 81.5% white, 12.0% black	31.4 (4.9)	132.5 (16.6)/ 81.5 (9.1)	4.9 (1.0)	NR/ 1.2(0.4)	NR	People with ADA risk score≥10 and casual capillary blood glucose (CCBG) of 110–199 mg/dl	Recruited from YMCA by a community-based screening	32.6
Almeida et al. 2011	53	12	Range: 20–29: 12% 30–39: 26% ≥40: 52%	18.9	Clinic Sao Paulo Brazil	23.3 (2.7)	111.1 (11.6)/ 75.2 (7.3)	4.8 (1.0)	2.8 (0.8)/ 1.2 (0.3)	1.5 (0.8)	Aged: 20-59yrs; without hyperlipidemia, hypertrygliceridemia, hyperglycemia, obesity, cancer, anabolic, or corticosteroid drugs use, or pregnancy	Recruited from a reference HIV clinic	20.8
Anderson et al. 2014 Craigie et al. 2011	329	12	63.6 (6.8)	26.0	Community Scotland UK 99.0% white	30.7 (4.2)	142.5 (17.8)/ 84 (10.0)	5.1 (1.2)	3.0 (1.1)/ 1.4 (0.4)	1.7 (1.1)	Aged: 50-74yrs; BMI>25kg/m2; with polypectomy for adenoma, without pregnancy, DM	Recruited from a bowel screening program	7.3
Anderssen et al. 1996 & 1998 Jacobs et al. 2009 The ODES Investigators 1993 Torjesen et al. 1997	219	12	44.9 (2.5)	9.6	Community Oslo Norway	28.8 (3.4)	131.5 (12.4)/ 90.1 (8.1)	6.3 (0.8)	NR/ 1.0 (0.2)	2.3 (1.1)	BMI>24 kg/m2 DBP: 86–99 mmHg TC: 5.20–7.74 mmol/L HDL-C<1.2 mmol/L TG>1.4 mmol/L	Recruited from a continuously ongoing screening program in Oslo	4.6
Arguin et al. 2012	25	12	60.5 (6.0)	100.0	Community Sherbrooke Quebec Canada	Weight (SD) 79.6 (10.7)	NR/ NR	5.8 (0.7)	3.5 (0.6)/ 1.5 (0.3)	1.8 (0.9)	Sedentary obese postmenopausal women without: (1) abnormal fasting lipid profile (2) CVD (3) DM	Using a computer-generated randomization list	12.0
Bazzano et al. 2014	148	12	46.8 (10.1)	88.5	Community New Orleans LA 45.3% white 51.4% black 2% Hispanic	35.4 (4.2)	122.6 (13.3)/ 78.4 (8.7)	5.2 (1.1)	3.2 (1.0)/ 1.4 (0.3)	1.3 (0.8)	Obese people (BMI: 30–45 kg/m2) without DM and CVD	Recruited from community screenings and TV ads	17.8
Bo et al. 2007&2009	375	48	55.7 (5.7)	58.2	Community Asti Italy	29.7 (4.4)	142.1 (14.7)/ 88.0 (9.2)	5.9 (1.1)	NR/ 1.4 (0.3)	1.9 (0.9)	People with MetS defined by FPG>110 mg/dL, without DM and CVD	Recruited from a metabolic screening	10.7
Bouchonville et al. 2014 Villareal et al. 2011	107	12	69.7 (4.0)	62.6	Community St. Louis MO	37.2 (5.0)	134.7 (18.8)/ 73.0 (10.1)	NR	NR/ 1.4 (0.4)	1.6 (0.7)	Old (≥65yrs) and obese (≥30 kg/m2) people without DM	Recruited from ads	13.0
Brinkworth et al. 2004	58	12	50.2 (NR)	77.6	Community Adelaide Australia	34.0 (NR)	132.0 (13.9)/ 75.1 (10.7)	5.6 (0.9)	3.8 (0.9)/ 1.0 (0.3)	1.9 (0.7)	Obese, hyperinsulinemic persons aged between 20 and 65yrs, insulin > 12 mu/l without DM	NR	25.9
Broekhuizen et al. 2012	340	12	45.3 (12.9)	56.7	Community Amsterdam The Netherland	26.5 (5.0)	124.5 (15.0)/ NR	5.2 (1.3)	3.6 (1.3)/ 1.2 (0.4)	1.2 (0.6)	Aged: 18-70yrs, with familial hypercholesterolemia, a LDL-C level>75th percentile	Recruited from the national cascade screening program	7.4
Burke V, et al. 2007 & 2008	241	36	56.2 (7.3)	55.6	Community Perth Australia	30.1 (2.7)	126.5 (9.5)/ 76.5 (7.5)	5.1 (0.9)	NR/ 1.3 (0.3)	1.3 (0.7)	Overweight, age>40yrs persons using 1 or 2 drugs to treat HT >3 Months without DM, chronic renal failure, CVD	Recruited by media advertising	16.2
Burtscher et al. 2009&2012	36	12	57.5 (6.9)	55.6	Clinic Innsbruck Austria	29.0 (3.9)	191 0 (25.9)/ 91.6 (11.0)	5.8 (1.0)	NR/ 1.4 (0.4)	NR	Patients with IFG (FPG:100–125 mg/dl), aged: 40-65yrs; BMI>25 kg/m2, and without DM	Recruited from family physicians through screening	0.0
Chirinos 2016	120	12	51.7 (8.4)	55.8	Clinics Coral Gables FL 84.0% Hispanic 10.9% black	NR	125.2 (16.8)/ 79.3 (9.5)	NR	NR/ 1.0 (0.2)	2.4 (1.1)	Aged: 30-70yrs, obese adults with WC≥102 cm for males, 88 cm for females, TG≥ 150 mg/dl, HDL-C< 40 mg/dl for males, <50mg/dl for females, IFG≥100 mg/dl.	Recruited from low-income community clinics	22.5
Choo et al. 2014	110	12	43.1 (9.0)	100.0	Community Seoul South Korea	28.5 (3.8)	116.5 (13.1)/ NR	5.5 (1.0)	3.3 (0.9)/ 1.4 (0.3)	1.5 (0.9)	Age: 18-65yrs; elevated waist circumference (≥85cm), abdominal obesity without DM and CVD	Recruited via poster, leaflet, telephone, and ads	55.5
Clifton et al. 2008	119	12	49.0 (9.0)	100.0	Community Adelaide Australia	32.8 (3.5)	NR/ NR	5.8 (1.1)	3.9 (0.9)/ 1.3 (0.3)	1.4 (0.6)	Women, aged: 20-65yrs, BMI:27-40kg/m2, without DM, or renal or liver disease	Recruited from public ads and screened	33.6
Cole et al. 2013	94	12	58.3 (9.6)	46.0	Community San Antonio TX 64.0% white, 17.0% black, 19.0% Hispanic	30.8 (4.9)	143.0 (17.0)/ 83.0 (10.0)	5.0 (1.0)	2.9 (0.9)/ 1.4 (0.4)	1.8 (1.4)	Aged:18+yrs; without DM, but with pre-DM, by ADA defined IFG (100–125 mg/dL)	Recruited from a pre-DM education class	31.0
Coon et al. 1989	20	12	59.5 (7.5)	0.0	Community Baltimore MD	29.0 (3.0)	NR/ NR	4.6 (0.7)	3.1 (0.7)/ 0.8 (0.2)	1.5 (0.4)	Aged 45+yrs, healthy persons without DM	Recruited by ads	0.0
Cox et al 2006 & 2008 & 2010	116	12	55.5 (4.7)	100.0	Community Berth Western Australia	26.4 (3.3)	NR/ NR	5.2 (0.7)	3.2 (0.7)/ 1.5 (0.3)	1.1 (0.5)	Aged: 50-70yrs; BMI<34 kg/m2; non-smoker, with sedentary lifestyle, without DM	Recruited by ads.	25.9
Ditschuneit et al. 1999 & 2001	100	24	45.7 (10.6)	79.0	Clinics Ulm Germany	33.4 (3.6)	139 5 (14.5)/ 82.5 (6.0)	5.9 (1.0)	NR/ 1.3 (0.4)	2.2 (1.3)	Age>18yrs, BMI between 25 and 40 kg/m2 without endocrine disorders	Recruited by referring to the obesity clinics	27.0
Donnelly et al. 2000	22	18	51.5 (8.5)	100.0	Community Kearney NE	31.2 (4.0)	133.0 (16.1)/ 80.5 (9.2)	4.9 (1.1)	NR/ 1.1 (0.3)	NR	BMI>25 kg/m2, low aerobic capacity, at risk for continued weight gain	NR	0.0
Esposito et al. 2003	120	24	34.6 (5.0)	100.0	Clinic Naples Italy	34.9 (2.4)	123.5 (8.2)/ 85.0 (4.8)	5.1 (0.6)	NR/ 1.2 (0.3)	1.6 (0.6)	Obese premenopausal women, aged: 20-46yrs; without DM, IGT (140–200 mg/dl), CAD, pregnancy. OGTT confirmed	Recruited from an outpatient dept.	6.7
Esposito et al. 2004a	110	24	43.3 (5.0)	0.0	Clinic Naples Italy	36.7 (2.4)	127.5 (7.6)/ 85.5 (3.9)	5.5 (0.8)	NR/ 1.0 (0.3)	1.9 (0.6)	Obese men with erectile dysfunction, aged:35-55yrs; without DM and IGT, OGTT confirmed	Recruited from an outpatient department list	5.5
Esposito et al. 2004b (JAMA v.292) & 2009	180	24	43.9 (6.2)	45.0	Clinic Naples Italy	28.0 (3.3)	135.0 (9.5)/ 85.5 (6.5)	5.1 (0.9)	NR/ 1.1 (0.2)	1.9 (0.6)	Sedentary people with MetS, FPG≥110 mg/dL,	Recruited from a screening program	8.9
Fatouros et al. 2005	50	12	70.4 (3.8)	0.0	Community Alexandroupolis Greece	29.5 (3.3)	NR/ NR	NR	NR/ NR	NR	Inactive old men, nonsmoker, without DM, FPG≤7 mmol/L	Recruited from a volunteer database in local community	0.0
Fernandez et al. 2012	40	12	40.9 (13.5)	67.5	Community Leon Spain	31.8 (2.4)	124.8 (17.6)/ 78.5 (12.6)	5.2 (0.9)	3.1 (0.7)/ 1.4 (0.5)	1.7 (1.0)	Aged: 18-70yrs; BMI: 28–35 kg/m2; without DM and pregnancy	Recruited from a clinic trial	60.0
Ferrara et al. 2012	188	24	56.4 (9.5)	47.9	Clinic Naples Italy	29.2 (4.5)	134.1 (16.0)/ 84.4 (10.6)	5.1 (0.9)	3.2 (0.9)/ 1.3 (0.3)	1.5 (1.0)	People with HT	Recruited from an outpatient clinic	0.0
Fischer et al. 2016	163	12	46.4 (11.5)	75.8	Clinics Denver CO	NR	118.8 (14.1)/ NR	NR	NR/ NR	NR	Patients aged 18+yrs, with A1C: 5.7–6.4%; BMI: 25–50 kg/m2; without DM	Recruited from health centers	5.7
Fisher et al. 2012	97	12	Range: 21–46	100.0	Community Birmingham AL 53.6% black; 46.4% white	28.0 (1.0)	NR/ NR	NR	NR/ NR	NR	Aged: 21-46yrs; BMI: 27–30 kg/m2; non-smoker, with sedentary lifestyle premenopausal women	Recruited from a previous parent study	0.0
Fogelholm et al. 2000	82	24	Range: 30–45	100.0	Community Tampere Finland	34.0 (3.6)	119.0 (10.0)/ 78.0 (7.0)	5.0 (0.9)	NR/ 1.2 (0.2)	1.3 (0.5)	Aged: 30-45yrs, BMI: 30–45 kg/m2, physical inactive	Recruited by ads	9.8
Fonolla et al. 2009	297	12	46.0 (8.4)	15.5	Community Granada Spain	28.8 (5.0)	122.1 (15.2)/ 79.5 (9.0)	5.6 (1.0)	3.7 (1.0)/ 1.1 (0.3)	1.6 (1.2)	People with moderate risk of CVD, without DM and pregnancy	Recruited from a screening program	14.8
Frank et al. 2005	173	12	60.7 (6.7)	100.0	Community Seattle Washington	30.4 (3.9)	NR/ NR	NR	NR/ NR	1.4 (0.6)	Postmenopausal women, aged: 50-75yrs, sedentary at baseline BMI≥25 kg/m2 without DM, nonsmoker	Recruited through a combination of mailings and media placements	1.7
Groeneveld et al. 2008 & 2010	816	12	46.6 (9.0)	0.0	Community Amsterdam The Netherlands	28.5 (3.5)	142.9 (15.3)/ 88.8 (9.6)	NR	NR/ 1.1 (0.2)	NR	Male construction workers with elevated risk of CVD	Recruited from Periodical Health Screening	27.6
Heshka et al. 2003	423	24	44.5 (10.0)	84.6	Clinics NY, Medison, Baton Rouge, Boulder, Davis, Durham, Woodbury	33.7 (3.6)	122.0 (13.0)/ 79.0 (8.5)	5.5 (1.0)	NR/ 1.3 (0.3)	1.7 (1.0)	Aged: 18-65yrs; BMI: 27–40 kg/m2; with FPG<7.8 mmol/L,	Recruited from existing clinic records, or by ads	27.0
Imayama et al. 2013 Foster-Schubert et al. 2012 Mason et al. 2011&2013	439	12	58.0 (5.0)	100.0	Community Seattle WA 85.0% white	30.9 (4.1)	NR/ NR	NR	NR/ NR	NR	Aged: 50-75yrs; BMI: ≥25 kg/m2; <100 min/w PA; postmenopausal; without DM; FPG<126 mg/dL	Recruited from mass mailing ads	9.1
Juul et al. 2016	127	12	NR	68.6	Community Holstebro Denmark	NR	133.0 (14.1)/ 82.5 (8.5)	5.3 (1.1)	3.2 (0.9)/ 1.3 (0.3)	NR	Aged<70yrs, FPG: 6.1–6.9 mmol/l; A1C: 6.0-<6.5%	Recruited from a referral	15.0
Kanaya et al. 2012 Delgadillo et al. 2010	238	12	56.5 (16.5)	73.5	Community Berkeley, Oakland, etc CA 22.5% white, 23.0% black, 37.0% Hispanic	30.0 (5.7)	127.2 (20.0)/ NR	NR	3.0 (1.1)/ 1.4 (0.4)	1.6 (1.2)	Aged: 25+yrs; a capillary blood glucose:106–160 mg/dL, without DM	Recruited from a community-based education outreach	12.2
Kanaya et al. 2014	180	12	55.0 (7.0)	72.0	Clinics San Francisco, San Diego CA 65% white	34.3 (6.7)	124.0 (14.0)/ 72.5 (9.0)	5.3 (1.0)	3.2 (0.9)/ 1.3 (0.3)	1.8 (0.8)	Aged: 21-65yrs; with MetS (FPG:100–125 mg/dL), HT, and underactive lifestyle (<150min/w of moderate intensity activity), without DM	Recruited by ads and flyers in community and clinical settings	21.1
Katula et al. 2010&2011&2013	301	24	57.9 (9.5)	57.5	Community Winston-Salem NC 73.8%white, 24.6%black	32.7 (4.0)	NR/ NR	NR	NR/ NR	NR	Patients with pre-DM defined by FPG of 95–125 mg/dl and BMI of 25–39 kg/m2 and without DM and CVD	Recruited from mass mailing, community health fair or referrals	12.6
Kawano et al. 2009	217	17	60.9 (13.8)	66.5	Community Saga City Japan	23.7 (4.4)	127.5 (17.8)/ 72.3 (8.9)	5.3 (0.9)	3.1 (0.7)/ 1.5 (0.4)	1.4 (0.8)	People with FPG: 100–140 mg/dL, or A1C: 5.5–6.0%	Recruited from health checkup	27.2
Keogh et al. 2007	36	12	48.6 (5.2)	68.0	Community Adelaide Australia	32.9 (4.5)	122.0 (10.8)/ 75.0 (3.6)	5.5 (1.4)	3.6 (1.4)/ 1.3 (0.4)	1.6 (0.6)	Overweight or obese people, aged: 20-65yrs; BMI: 27–40 kg/m2; without DM, with FPG≤7.0mmol/L.	Recruited from newspaper ads	30.6
Lawton et al. 2009	1089	24	58.9 (6.9)	100.0	Clinics Wellington New Zealand	29.2 (6.0)	123.1 (17.5)/ 74.3 (9.3)	6.1 (1.2)	NR/ 1.6 (0.5)	NR	Physically inactive women, aged: 40-74yrs without medical condition	Recruited by invitation letters or practice register	7.4
Lim et al. 2010	113	12	47.0 (10.0)	82.3	Community Adelaide Australia	32.0 (6.0)	127.0 (12.6)/ 76.3 (10.2)	5.6 (1.0)	2.9 (1.7)/ 1.3 (0.3)	1.6 (0.8)	Aged: 20-65yrs, BMI: 28–40 kg/m2, with at least one CVD risk factor, without DM	Recruited by ads	38.9
Lombard et al. 2010	250	12	40.4 (4.8)	100.0	Community Melbourne Australia	27.8 (5.4)	NR/ NR	4.9 (0.9)	2.6 (0.8)/ 1.7 (0.4)	1.0 (0.7)	Women with a child in schools without pregnancy and serious medical conditions	Recruited through an invitation attached to school newsletter	14.0
Ma et al. 2009&2013	241	15	52.9 (10.6)	47.0	Clinic San Francisco CA 78% white, 17% Asian	32.0 (5.4)	118.8 (11.7)/ 73.6 (8.3)	4.9 (0.9)	2.8 (0.8)/ 1.2 (0.3)	1.9 (0.8)	Patients aged≥18yrs, BMI≥25 kg/m2, with pre-DM defined by FPG of 100–125 mg/dl, or MetS	Recruited from a single primary care clinic	8.3
Marrero et al. 2016	225	12	52.0 (11.0)	84.4	Community Indianapolis IN 64.5% white 25.3% black	36.8 (7.2)	130.2 (14.0)/ 81.4 (8.5)	4.9 (0.9)	NR/ 1.2 (0.4)	NR	Aged 18+yrs, BMI>24 kg/m2 (>/ = 23 kg/m2 for Asian); ADA risk score≥5; A1C: 5.7–6.5%	Recruited from a screening	22.2
Marsh et al. 2010	96	12	30.2 (5.2)	100.0	Clinic Sydney Australia	34.5 (4.2)	NR/ NR	4.8 (0.7)	2.8 (0.7)/ 1.4 (0.7)	1.3 (0.7)	Women, aged: 18-40yrs; BMI<25 kg/m2, with polycystic ovary syndrome, without pregnancy and DM	Recruited from a screening program	49.0
Mason et al. 2016	194	12	47.0 (12.7)	78.0	Community San Francisco CA 58.8% white 12.9% black 11.9% Hispanic	35.5 (3.6)	NR/ NR	NR	NR/ NR	NR	Obese adults aged 18+yrs, with BMI: 30–45.9 kg/m2; WC>102 cm for males, >88 cm for females, without DM, confirmed by FPG<126 mg/dl	Recruited from community by newspaper ads.	23.2
McAuley et al. 2005&2006	93	12	Range: 30–70	100.0	Community Dunedin New Zealand	35.7 (5.0)	126.8 (13.0)/ 81.9 (10.0)	5.8 (1.0)	3.8 (0.8)/ 1.2 (0.3)	1.9 (0.7)	Overweight women, aged: 30-70yrs; BMI:>27 kg/m2; without pregnancy	Recruited by local ads	18.3
Mellberg et al. 2014	70	24	59.9 (5.7)	100.0	Community Umea Sweden	32.7 (3.5)	139.5 (13.0)/ 83.0 (8.3)	5.7 (1.1)	3.8 (1.0)/ 1.4 (0.4)	1.2 (0.6)	Postmenopausal non-smoking women, BMI≥27 kg/m2, without DM, FPG<7 mmol/L	Recruited by newspapers ads	30.0
Muto et al. 2001	326	18	42.5 (3.7)	0.0	Community Tokyo Japan	24.7 (3.0)	123.2 (15.6)/ 78.4 (12.1)	5.5 (0.9)	NR/ 1.3 (0.4)	2.3 (1.4)	Male workers with at least one abnormality, including FPG>100 mg/dL	Recruited from a building maintenance company	7.4
Narayan et al. 1998	95	12	Range: 25–50	75.8	Community Pima AZ	Range: 20.2–59.9	Range: 90.0-176/ 48.0–98.0	Range: 2.1–6.1	NR/ NR	Range: 0.3–3.6	Overweight/obese people, aged: 25-54yrs; BMI>25kg/m2, without DM, OGTT<7.8mmol/L	Recruited from an epidemiological study	2.0
Nilsson et al. 1992	94	12	55.0 (7.2)	NR	Community Dalby Sweden	Weight (kg): 81.4 (11.6)	145.0 (18.0)/ 84.3 (7.6)	5.6 (0.8)	3.9 (0.7)/ 0.9 (0.2)	1.6 (0.7)	Patients with or without HT, but no DM	Recruited from a cross-sectional study	8.5
Nilsson et al 2001	113	18	49.7 (6.2)	60.9	Community Helsingborg Sweden	27.8 (5.6)	132.5 (18.0)/ 77.4 (9.7)	5.8 (0.9)	3.9 (0.9)/ 1.2 (0.3)	1.3 (0.7)	Aged: 40-50yrs; with a cardiovascular risk score sum of ≥9	Recruited from a screening program	18.6
Ockene et al. 2012 Merriam et al. 2009	312	12	52.0 (11.2)	74.4	Community Lawrence MA	33.9 (5.6)	128.7 (12.4)/ NR	NR	NR/ 1.2 (0.3)	NR	Age>25+yrs, BMI>24kg/m2, with risk for DM, but without DM	Recruited from the Greater Lawrence Family Health Center	7.4
Poston et al. 2006	250	12	41.0 (8.5)	92.4	Community Huston TX	36.1 (3.1)	121.5 (12.0)/ 72.3 (8.6)	5.2 (1.0)	3.1 (0.8)/ 1.4 (0.3)	1.5 (0.8)	Overweight/obese people, aged: 25-55yrs; BMI: 27–40 kg/m2; without DM or pregnancy, FPG<7mmol/L, confirmed by OGTT	Recruited from a screening program	45.6
Potteiger et al. 2003 & 2002	66	16	NR	57.6	Community Denver CO	Range: 25–34.9	NR/ NR	NR	NR/ NR	NR	Sedentary people without DM and heart disease	Recruited from the Midwest Exercise Trial	10.1
Reid et al. 2014	426	12	51.5 (11.6)	61.3	Clinic Ottawa Canada 95.3% white	29.4 (5.7)	121.1 (16.1)/ 76.5 (9.5)	5.2 (1.0)	3.3 (0.9)/ 1.3 (0.4)	1.3 (0.8)	Obese people with coronary risk, without DM, pregnancy, FPG<7 mmol/L	Recruited from a care cardiac center by ads and flyers	25.8
Rossner et al. 1997	93	12	41.0 (NR)	67.7	Clinics Stockhlom Sweden	38.7 (4.5)	136.3 (16.9)/ 86.5 (12.2)	5.7 (0.9)	NR/ NR	1.9 (1.0)	Obese people with BMI> 30 kg/m2, without DM	Recruited from hospital waiting list	38.7
Ryttig et al. 1997	81	28	42.5 (10)	54.3	Clinics Stockhlom Sweden	37.7 (4.6)	136.2 (17.3)/ 85.3 (9.9)	5.7 (1.0)	NR/ 1.1 (0.2)	2.0 (1.2)	Obese people, aged: 21-64yrs; BMI:>30 kg/m2; without DM and pregnancy	Recruited from hospital waiting list	4.9
Sartorelli et al. 2005	104	12	45.5 (9.1)	79.8	Community Sao Paulo Brazil	28.7 (2.5)	116.6 (17.6)/ 77.5 (18.3)	5.3 (1.2)	3.6 (1.1)/ 1.2 (0.4)	1.6 (0.9)	Overweight or obese people, aged: 30-65yrs; BMI: 24–35 kg/m2; without DM, or pregnancy	Recruited from a screening of high-risk group for DM	31.7
Sattin et al. 2016	604	12	46.5 (10.9)	83.0	Community Augusta GA	35.7 (7.3)	130.5 (16.6)/ 82.6 (9.7)	NR	NR/ NR	NR	African Americans aged: 20-64yrs; BMI≥25 kg/m2; without DM, confirmed by FPG<126 mg/dl	Recruited from church	0.0
Simkin-Silverman et al. 1995 & 1998 & 2003 Kuller et al. 2001 & 2006&2012	535	54	47.0 (1.0)	100.0	Community Allegheny PN 92.0% white	25.1 (3.3)	110.0 (12.8)/ 68.0 (8.2)	4.9 (0.6)	3.0 (0.6)/ 1.5 (0.3)	0.9 (0.5)	Premenopausal women, aged: 44-50yrs; BMI: 20–34 kg/m2; FPG<7.8mmol/L	Recruited from the Women's Healthy Lifestyle Project	2.8
Siu et al. 2015	182	12	56.0 (9.1)	74.2	Community Hong Kong China	NR	133.8 (16.8)/ 82.4 (9.8)	NR	NR/ 1.2 (0.3)	2.2 (1.8)	Aged: 18-94yrs; with MetS by 1) WC: 90 cm for males, 80 cm for females; 2) SBP>130 mmHg, DBP>85 mmHg; 3) FPG>/ = 5.5 mmol/l; 4) TG>1.7 mmol/l; 5) HDL-C<40 mmol/l for males, 50 mmol/l for females	Recruited from a screening	35.7
Staten et al. 2004	361	12	57.2 (4.8)	100.0	Community Tucson AZ 100% Hispanics	29.5 (5.3)	124.8 (16.7)/ 74.1 (9.6)	5.6 (1.3)	NR/ NR	NR	Uninsured Hispanic women, aged≥50yrs,	Recruited from clinic registration	33.4
Stefanick et al. 1998	377	12	52.1 (7.3)	47.7	Community Palo Alto CA	26.7 (3.0)	115.5 (12.8)/ 73.2 (7.4)	6.2 (0.6)	4.2 (0.5)/ 1.2 (0.2)	1.8 (0.8)	Postmenopausal women, aged: 45-64yrs; men aged:30-64yrs; without DM, FPG<7.8mmol/L, OGTT confirmed	Recruited from the Diet and Exercise for Elevated Risk Trial	27.0
Tapsell et al. 2014	120	12	48.9 (9.3)	75.0	Community Wollongong Australia	30.0 (2.7)	NR/ NR	5.2 (0.9)	3.2 (0.8)/ NR	NR	Healthy adults aged 18-65yrs, BMI: 25–35 kg/m2, without DM	Recruited by ads in the local media	22.5
ter Bogt et al. 2009	457	12	56.1 (7.8)	57.9	Community Bilthoven The Netherlands	29.6 (3.4)	145.5 (17.0)/ 86.5 (8.9)	5.6 (1.0)	3.5 (0.9)/ 1.4 (0.4)	NR	Overweight or obese people, aged: 40-70yrs; BMI: 25–40 kg/m2; with HT or dyslipidemia, without DM	Recruited from a screening program	9.0
Thompson et al. 2005	90	12	41.4 (8.9)	85.6	Clinic Knoxville TN	34.8 (3.1)	NR/ NR	5.0 (0.9)	3.1 (0.9)/ 1.1 (0.3)	1.8 (1.2)	Obese people, aged: 25-70yrs; BMI: 30–40 kg/m2; without DM or pregnancy	Recruited from ad posters	13.3
Tsai et al. 2010	50	12	49.4 (11.9)	88.0	Clinic Philadelphia PA 81% black; 19% white	36.5 (6.0)	129.4 (12.2)/ 80.7 (8.2)	4.9 (0.9)	3.0 (0.9)/ 1.4 (0.3)	1.1 (0.7)	Overweight or obese people with BMI: 27–50 kg/m2, without serious psychiatric illness	Recruited from flyers, and referrals from PCPs	6.0
Vainionpaa et al. 2007	120	12	Range: 35–40	100.0	Community Oulu Finland	25.3 (4.6)	NR/ NR	5.3 (0.9)	3.2 (0.8)/ 1.7 (0.4)	1.0 (0.5)	Women with age: 35-40yrs, without chronic disease	Recruited from the National Population Register of Finland	33.3
Vetter et al. 2013 Wadden et al. 2011	390	24	51.5 (11.5)	79.7	Clinic Philadelphia PA 59% white, 38.5%black	38.5 (4.7)	121.4 (16.3)/ 76.2 (10.4)	4.6 (1.0)	2.9 (0.8)/ 1.1 (0.3)	1.3 (0.7)	Aged: 21+yrs; BMI: 30–50 kg/m2; with MetS (FPG≥110mg/dL); without cardiovascular events	Recruited from primary care practices	13.8
von Thiele Schwarz et al. 2008	195	12	46.6 (10.8)	100.0	Community Stockholm Sweden	NR	114.0 (16.9)/ 79.1 (11.6)	5.2 (1.0)	2.9 (0.8)/ 1.8 (0.4)	1.0 (0.6)	Working age women without DM and pregnancy	Recruited from a public dental health care organization	9.2
Watanabe et al. 2003	173	12	55.1 (7.1)	0.0	Community Tokyo Japan	24.4 (2.9)	121.7 (14.4)/ 76.9 (10.5)	5.2 (0.9)	NR/ 1.4 (0.4)	1.4 (0.8)	Male workers with risk for DM, aged:35-70yrs; OGTT confirmed	Recruited from annual check-up list	9.8
Weinstock et al. 1998	45	23	43.3 (7.4)	100.0	Community Syracuse NY	35.9 (6.0)	NR/ NR	NR	NR/ NR	NR	Women without DM, CAD, and pregnancy	Recruited from a cohort study	0.0
Weiss et al. 2006	48	12	56.8 (3.0)	63.2	Community St. Louis MO	27.3 (2.1)	NR/ NR	NR	NR/ NR	NR	Sedentary people, aged: 50-60yrs; BMI:23.5–29.9kg/m2; non-smoker without DM. FPG<7mmol/L, OGTT confirmed	Recruited from a screening program	4.2
Wing et al. 1995	202	18	37.4 (5.3)	48.1	Community Pittsburgh PA	30.9 (2.1)	111.7 (10.7)/ 71.8 (8.1)	5.0 (0.8)	NR/ 1.2 (0.2)	1.2 (0.7)	Aged: 25-45yrs; 13.6–31.8 kg above ideal body weight, without serious disease	Recruited from newspaper or radio ads	21.3
Wing et al. 1998	154	24	45.7 (4.4)	79.0	Community Pittsburgh PA	35.9 (4.3)	116.7 (14.9)/ 74.8 (10.1)	5.0 (0.8)	3.1 (0.8)/ 1.2 (0.3)	NR	Overweight people, aged:40-55yrs; with diabetic parents	Recruited from newspaper ads	22.0
Wycherley et al. 2012	123	12	50.8 (9.3)	0.0	Clinic Adelaide Australia	33.0 (3.9)	135.1 (12.5)/ 84.0 (10.7)	5.2 (0.9)	3.2 (0.8)/ 1.3 (0.4)	1.7 (0.7)	Overweight or obese males, aged: 20-65yrs; BMI: 27–40 kg/m2, without DM	Recruited by a screening program	44.7
Yeh et al. 2016	60	12	58.9 (10.9)	56.7	Community New York 100% Asian	26.1 (2.4)	126.9 (16.1)/ 78.4 (9.6)	4.8 (1.0)	2.8 (0.9)/ 1.4 (0.3)	1.4 (0.7)	Patients with pre-DM defined by A1C: 5.7–6.4% and BMI>/ = 24kg/m2	Recruited from hospital record	3.3
Mean (SD)			50.6 (8.7)			30.5 (4.6)	127.5 (15.2)/ 79.2 (9.3)	5.4 (1.0)	3.3 (0.9)/ 1.3 (0.3)	1.5 (0.9)			
Total Range	15618 20–1089	12–54		0–100		23.3–38.7							0–60.0

Abbreviations: BG: blood glucose; BL: baseline; BMI: body mass index; CAD: coronary Artery Disease; CVD: cardiovascular disease; DBP: diastolic blood pressure; DM: diabetes mellitus; FBG: fasting blood glucose; FPG: fasting plasma glucose; HDL-C: high density cholesterol; HT: hypertension; IGT: impaired glucose tolerance; LDL-C: low density cholesterol; MetS: metabolic syndrome; min/w: minutes/week; NR: not reported; OGTT: oral glucose tolerance test; PG: plasma glucose; SD: standard deviation; TC: total cholesterol; TG: triglycerides.

We observed considerable heterogeneity in the treatments provided to both intervention and comparison groups (Tables A&B in S1 File). In 29 studies, a similar approach was used in both intervention and control groups: data from these studies were synthesized by a single arm model, and are presented in Table C in S1 File as an online supplement. In the other 50 studies, UC was used in the control group. In the 50 studies that compared an intervention to UC, 38 had two arms, 5 studies[49,64,87,88,91] had 3 arms, and 7 studies[13,24,28,44,54,62,93] had 4 arms (e.g., PA, D, PA+D and control arm). The randomization procedure was described in 48 studies (Table B in S1 File). In 29 studies, allocation concealment was adequately reported. Meta-regression analyses indicated that there was no significant interaction between the between-group change in FPG and all study-level characteristics, such as mean age, publication date, the length of F/U, number of contacts, attrition, and their interaction terms. An Egger’s plot demonstrated a symmetrical shape distribution (except for two outliers) which is consistent with no publication bias.

### Changes in CVD risk factors

In 57 studies or study arms comparing interventions to UC with attrition <30%, the pooled effect estimate from all studies demonstrated that compared to UC, all lifestyle interventions, including PA, D, or PA+D interventions, achieved significant improvements in SBP (-2.05mmHg [95%CI, -2.81, -1.28]), DBP (-1.65mmHg [-2.16, -1.14]), TC (-0.09mmol/L [-0.14, -0.04]), LDL-C (-0.08mmol/L [-0.13, -0.03]), HDL-C (0.03mmol/L [0.01, 0.04]), and TG (-0.08mmol/L [-0.14, -0.03]) (Table 2). When including the 15 studies with attrition ≥30% in the sensitivity analysis, we observed similar effects. The remaining results are limited to studies with attrition <30%.

**Table 2:** Lifestyle interventional effect: Meta-analyses results.

	SBP (mmHg)			DBP (mmHg)			TC (mmol/L)			LDL-C (mmol/L)			HDL-C (mmol/L)			TG (mmol/L)		
	Studies (sample size)	Pooled effect mean (effect size) (95% CI)	Hetero- Geneity p value	Studies (sample size)	Pooled effect mean (effect size) (95% CI)	Hetero- Geneity p value	Studies (sample size)	Pooled effect mean (effect size) (95% CI)	Hetero- Geneity p value	Studies (sample size)	Pooled effect mean (effect size) (95% CI)	Hetero- Geneity p value	Studies (sample size)	Pooled effect mean (effect size) (95% CI)	Hetero- Geneity p value	Studies (sample size)	Pooled effect mean (effect size) (95% CI)	Hetero- Geneity p value
LI vs UC (all studies*)	42 (8331)	-2.05 (0.06) (-2.81, -1.28)	<0.01	39 (7631)	-1.65 (0.07) (-2.16, -1.14)	<0.01	36 (6925)	-0.09 (0.04) (-0.14, -0.04)	<0.01	27 (4563)	-0.08 (0.05) (-0.13, -0.03)	<0.01	43 (8414)	0.03 (0.03) (0.01, 0.04)	<0.01	38 (5926)	-0.08 (0.03) (-0.14, -0.03)	<0.01
LI vs UC (all studies^ƚ^)	50 (9053)	-2.13 (0.04) (-2.88, -1.38)	<0.01	46 (8261)	-1.57 (0.06) (-2.07, -1.07)	<0.01	44 (7541)	-0.11 (0.05) (-0.16, -0.06)	<0.01	34 (5087)	-0.09 (0.04) (-0.15, -0.04)	<0.01	52 (9212)	0.03 (0.03) (0.01, 0.04)	<0.01	46 (6632)	-0.08 (0.04) (-0.13, -0.03)	<0.01
LI vs UC (Group 1^ⱡ^)	17 (3492)	-0.95 (0.04) (-1.75, -0.15)	0.02	15 (2949)	-1.40 (0.06) (-2.24, -0.56)	<0.01	16 (2904)	-0.06 (0.03) (-0.13, 0.01)	<0.01	15 (3065)	-0.08 (0.05) (-0.14, -0.02)	<0.01	19 (3770)	0.01 (0.03) (0.00, 0.03)	0.06	19 (3240)	-0.04 (0.02) (-0.10, 0.02)	0.19
LI vs UC (Group 2^§^)	25 (4839)	-2.89 (0.08) (-3.95, -1.83)	<0.01	24 (4682)	-1.83 (0.08) (-2.50, -1.17)	<0.01	20 (4021)	-0.12 (0.06) (-0.18, -0.05)	<0.01	12 (1498)	-0.10 (0.06) (-0.18, -0.01)	0.02	24 (4644)	0.04 (0.06) (0.02, 0.06)	<0.01	20 (2686)	-0.12 (0.05) (-0.21, -0.04)	<0.01
LI vs UC (F/U = 12m)	34 (6616)	-2.07 (0.05) (-3.19, -0.95)	<0.01	31 (5916)	-1.62 (0.06) (-2.29, -0.95)	<0.01	29 (5813)	-0.06 (0.04) (-0.10, -0.01)	<0.01	23 (3643)	-0.08 (0.05) (-0.13, -0.02)	<0.01	33 (6782)	0.02 (0.05) (0.01, 0.03)	<0.01	27 (3959)	-0.08 (0.04) (-0.14, -0.03)	<0.01
LI vs UC (F/U = 13-23m)	6 (1418)	-1.73 (0.08) (-2.80, -0.65)	0.98	6 (1436)	-1.25 (0.08) (-2.02, -0.48)	0.60	6 (974)	-0.19 (0.17) (-0.26, -0.11)	0.46	5 (1033)	-0.12 (0.10) (-0.19, -0.05)	0.36	7 (1494)	0.00 (0.0) (-0.03, 0.03)	0.37	7 (1494)	-0.08 (0.03) (-0.21, 0.05)	<0.01
LI vs UC (F/U≥24m)	14 (3123)	-1.58 (0.05) (-2.71, -0.45)	<0.01	14 (3122)	-1.36 (0.05) (-2.30, -0.41)	<0.01	13 (2788)	-0.07 (0.03) (-0.17, 0.03)	<0.01	5 (543)	0.06 (0.04) (-0.07, 0.20)	0.39	14 (3122)	0.05 (0.06) (0.02, 0.08)	<0.01	13 (2034)	-0.08 (0.03) (-0.19, 0.03)	<0.01
PA vs UC	7 (1466)	-0.72 (0.03) (-1.89, 0.44)	0.22	7 (1465)	-1.12 (0.05) (-2.34, 0.10)	0.22	6 (1429)	-0.02 (0.01) (-0.09, 0.06)	0.76	3 (256)	-0.03 (0.02) (-0.18, 0.12)	0.91	7 (1463)	0.01 (0.02) (-0.02, 0.04)	0.10	6 (375)	-0.10 (0.08) (-0.22, 0.02)	0.48
D vs UC	4 (263)	-1.45 (0.07) (-3.83, 0.94)	0.23	4 (263)	-2.28 (0.16) (-4.07, -0.49)	0.74	3 (228)	-0.17 (0.13) (-0.34, -0.01)	0.89	3 (228)	-0.14 (0.11) (-0.30, 0.02)	0.99	4 (263)	0.00 (0.00) (-0.04, 0.04)	0.78	4 (263)	-0.15 (0.07) (-0.41, 0.10)	0.14
PA+D vs UC	31 (6602)	-2.29 (0.06) (-3.19, -1.40)	<0.01	28 (5903)	-1.66 (0.07) (-2.24, -1.09)	<0.01	27 (5268)	-0.10 (0.05) (-0.16, -0.05)	<0.01	21 (4079)	-0.08 (0.04) (-0.14, -0.02)	<0.01	32 (6688)	0.03 (0.07) (0.02, 0.05)	<0.01	29 (5288)	-0.07 (0.03) (-0.13, -0.01)	0.02

Abbreviations: D: dietary; DBP: diastolic blood pressure; HDL-C: high density lipoprotein cholesterol; LDL-C: low density lipoprotein cholesterol; LI: lifestyle intervention; m: month; NA: not applicable; PA: physical activity; SBP: systolic blood pressure; TC: total cholesterol; TG: triglycerides; UC: usual care; vs: versus

* All studies with attrition <30%.

ƚ All studies with attrition <30% plus studies with attrition ≥30%.

ǂ All studies with attrition <30% and participants with FPG<5.5 mmol/L or A1C <5.5%.

§ All studies with attrition <30% and participants with FPG≥5.5 mmol/L or A1C≥5.5%.

### Comparison according to participant baseline glycemic level

In the 39 studies among persons with low range glycemic level, lifestyle interventions were associated with significantly improved SBP (-0.95mmHg [-1.75, -0.15]), DBP (-1.40mmHg [-2.24, -0.56]), LDL-C (-0.08mmol/L [-0.14, -0.02]), and HDL-C (0.01mmol/L [0.00, 0.03])), except for TC (-0.06mmol/L [-0.13, 0.01]) and TG (-0.04mmol/L [-0.10, 0.02). In the 40 studies among persons with high range glycemic level, lifestyle interventions significantly improved most CVD risk indicators, and the improvements were more substantial: SBP (-2.89mmHg [-3.95, -1.83]), DBP (-1.83mmHg [-2.50, -1.17]), TC (-0.12mmol/L [-0.18, -0.05]), LDL-C (-0.10mmol/L [-0.18, -0.01]), HDL-C (0.04mmol/L [0.02, 0.06]), and TG (-0.12mmol/L [-0.21, -0.04]).

### Comparison according to intervention modality

**Fig 2:**
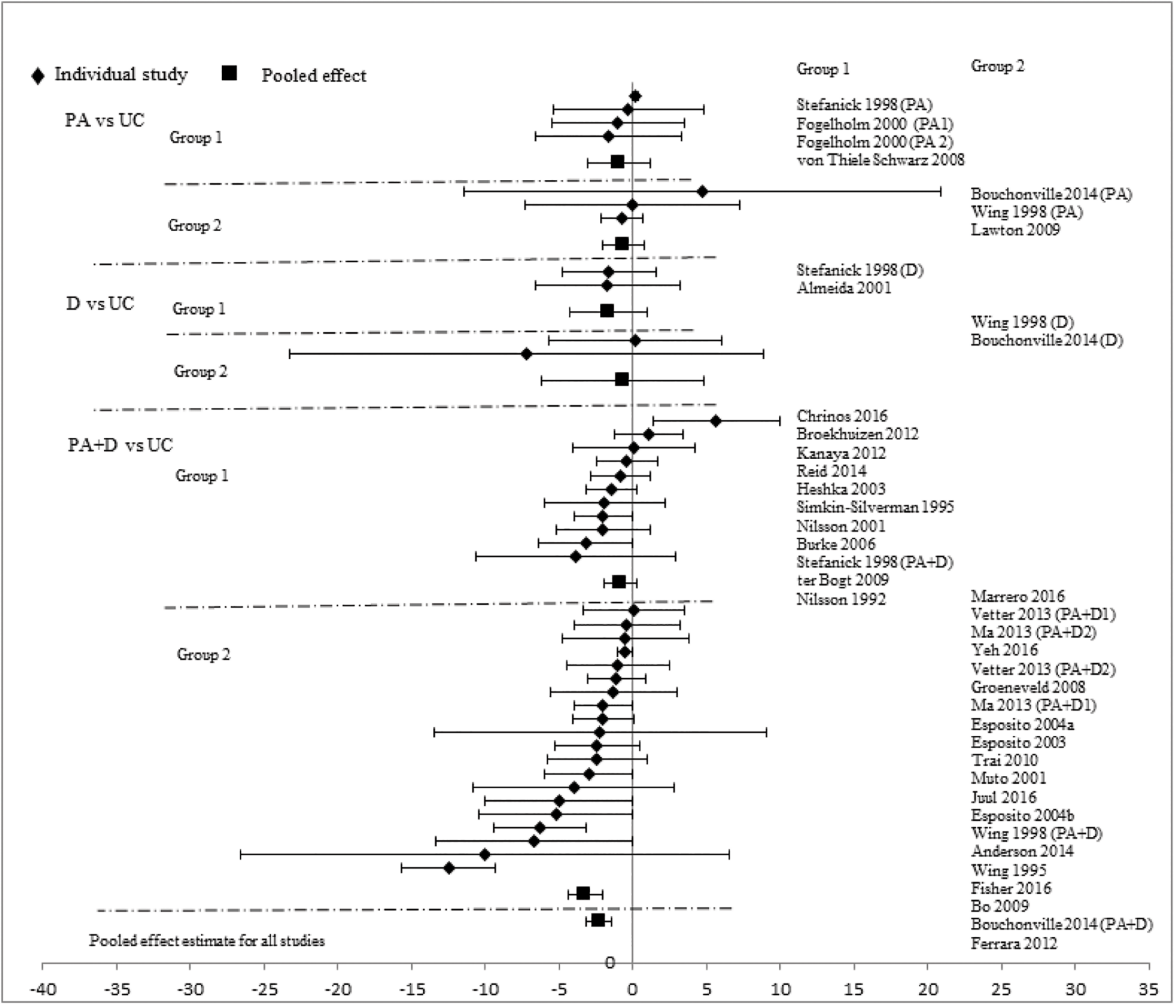
changes in systolic blood pressure in the intervention versus usual care groups (mmHg). Group 1: low-range glycemic group (FPG<5.5mmol/L or A1C <5.5%). Group 2: high-range glycemic group (FPG ≥5.5mmol/L or A1C ≥5.5%). D, diet, PA, physical activity, UC, usual care, vs, versus.

**Fig 3:**
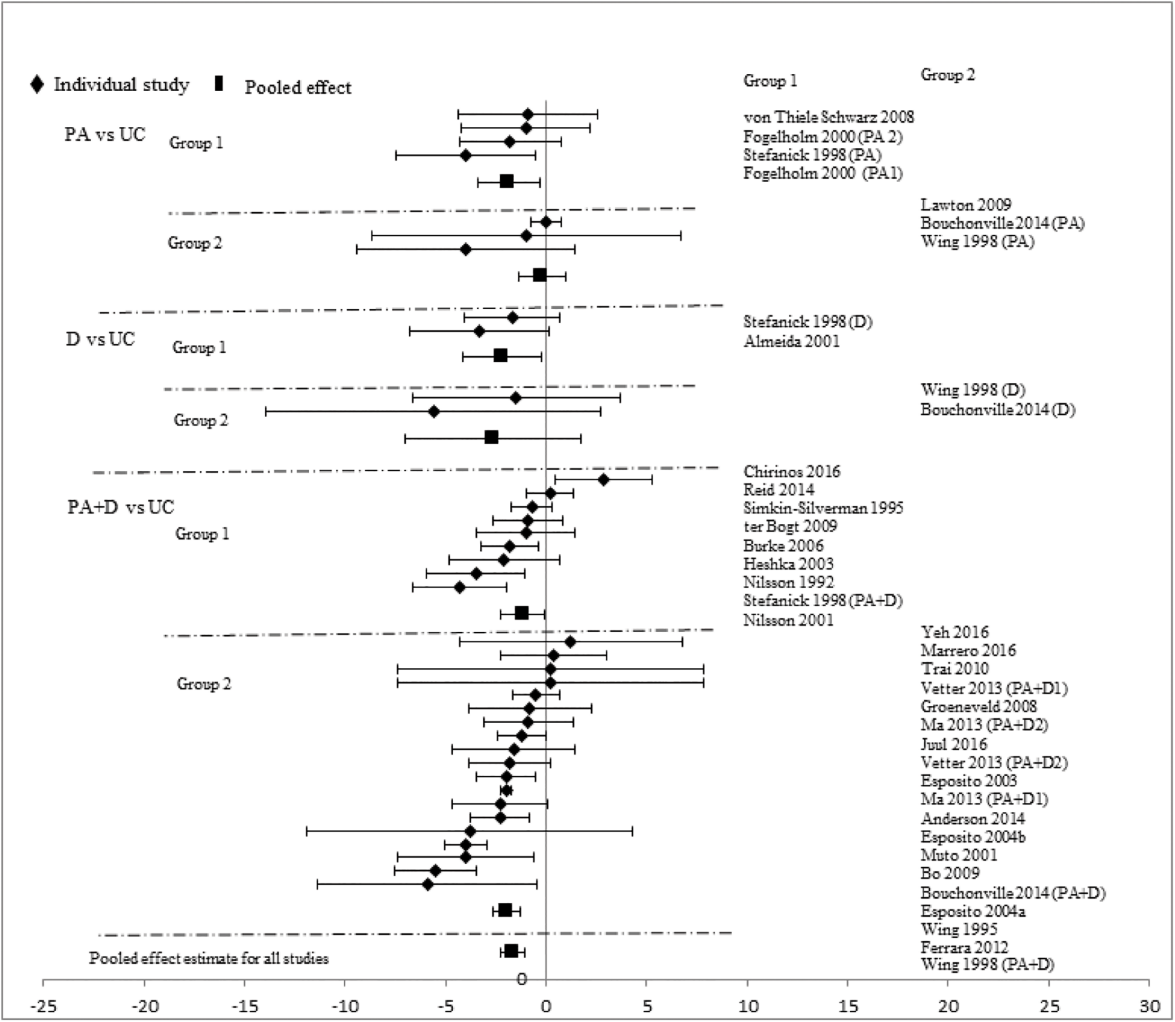
changes in diastolic blood pressure in the intervention versus usual care groups (mmHg). Group 1: low-range glycemic group (FPG<5.5mmol/L or A1C <5.5%). Group 2: high-range glycemic group (FPG ≥5.5mmol/L or A1C ≥5.5%). D, diet, PA, physical activity, UC, usual care, vs, versus.

**Fig 4:**
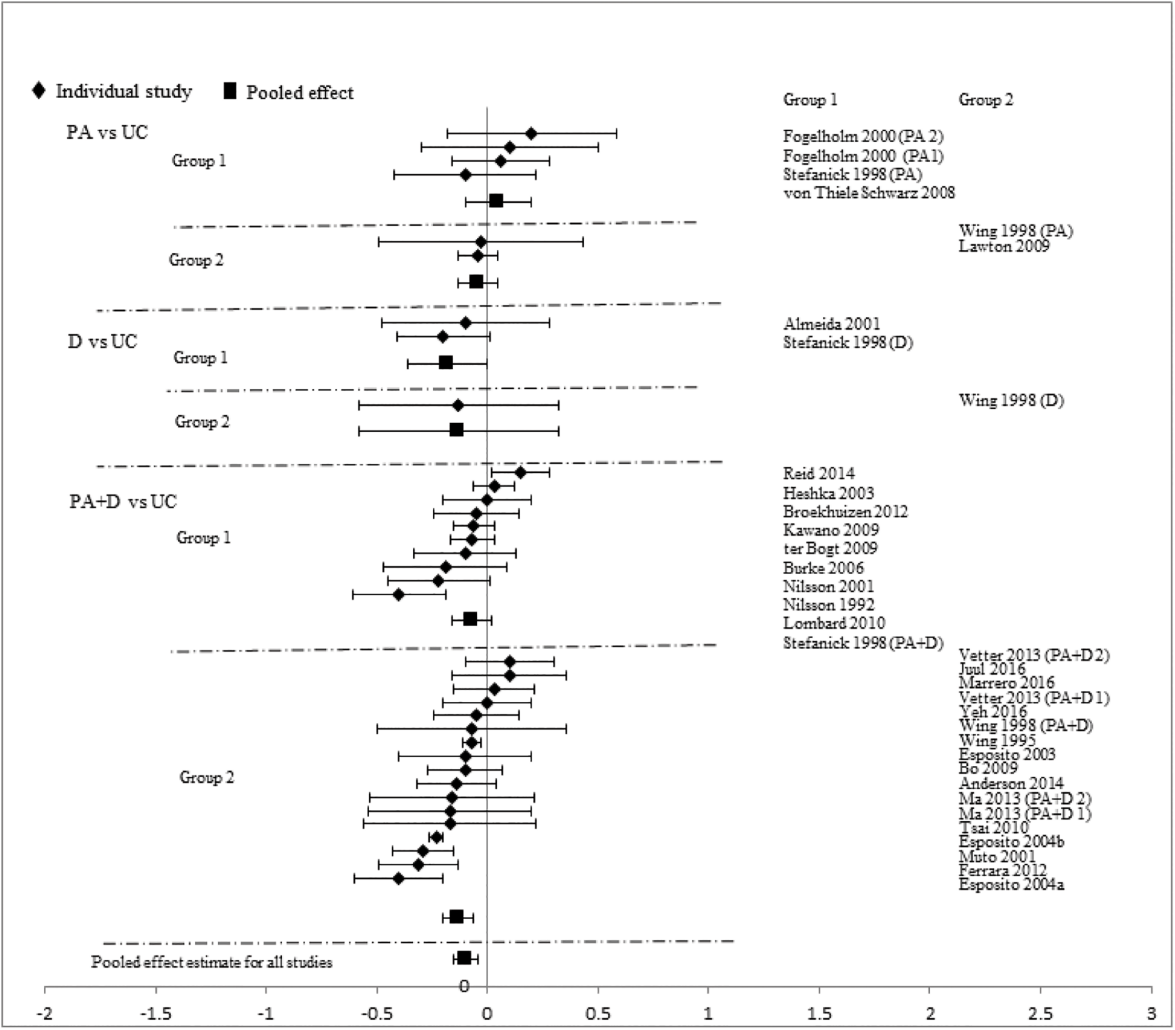
changes in total cholesterol in the intervention versus usual care groups (mmol/L). Group 1: low-range glycemic group (FPG<5.5mmol/L or A1C <5.5%). Group 2: high-range glycemic group (FPG ≥5.5mmol/L or A1C ≥5.5%). D, diet, PA, physical activity, UC, usual care, vs, versus.

**Fig 5:**
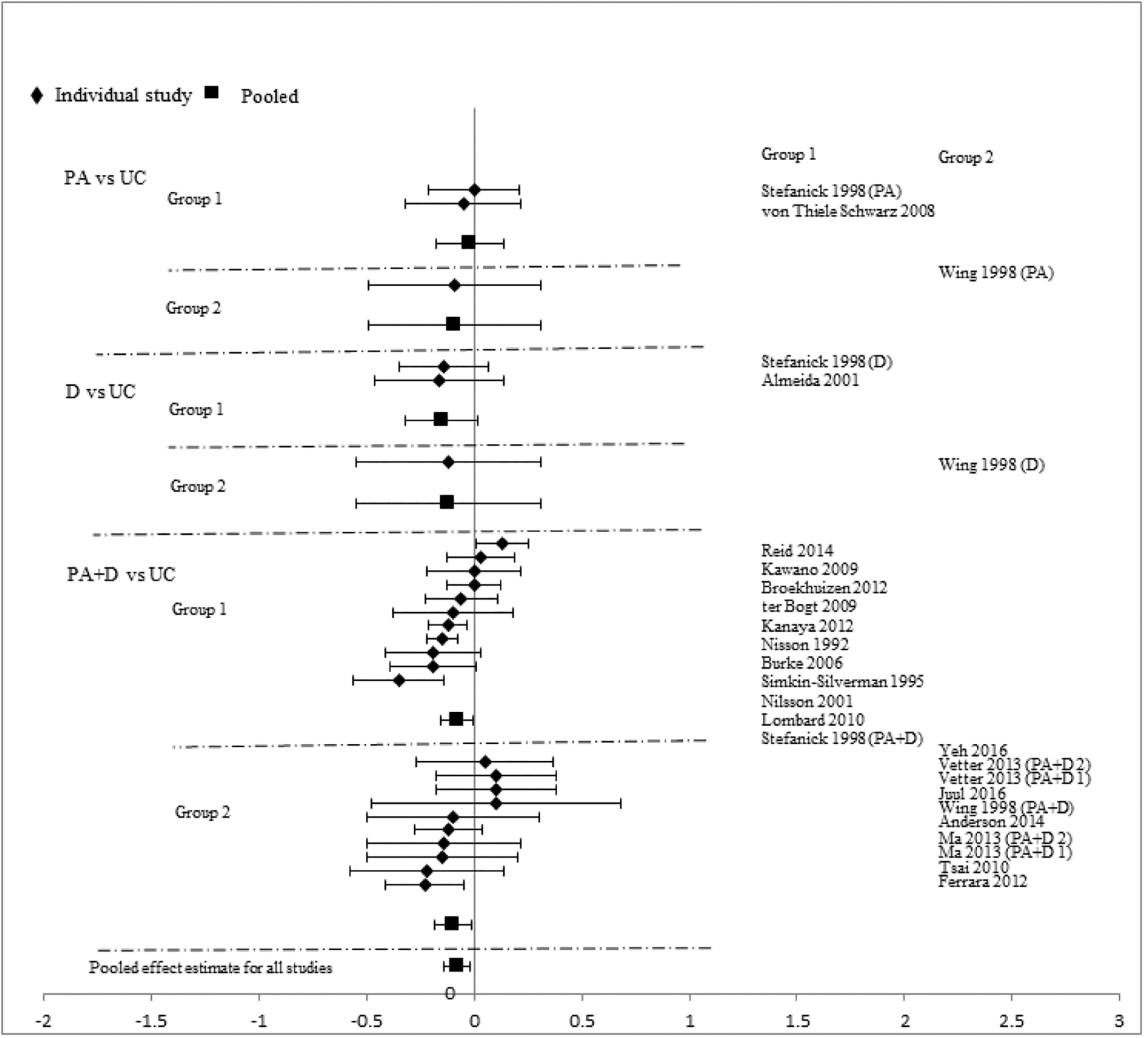
changes in low density lipoprotein cholesterol in the intervention versus usual care groups (mmol/L). Group 1: low-range glycemic group (FPG<5.5mmol/L or A1C <5.5%). Group 2: high-range glycemic group (FPG ≥5.5mmol/L or A1C ≥5.5%). D, diet, PA, physical activity, UC, usual care, vs, versus.

**Fig 6:**
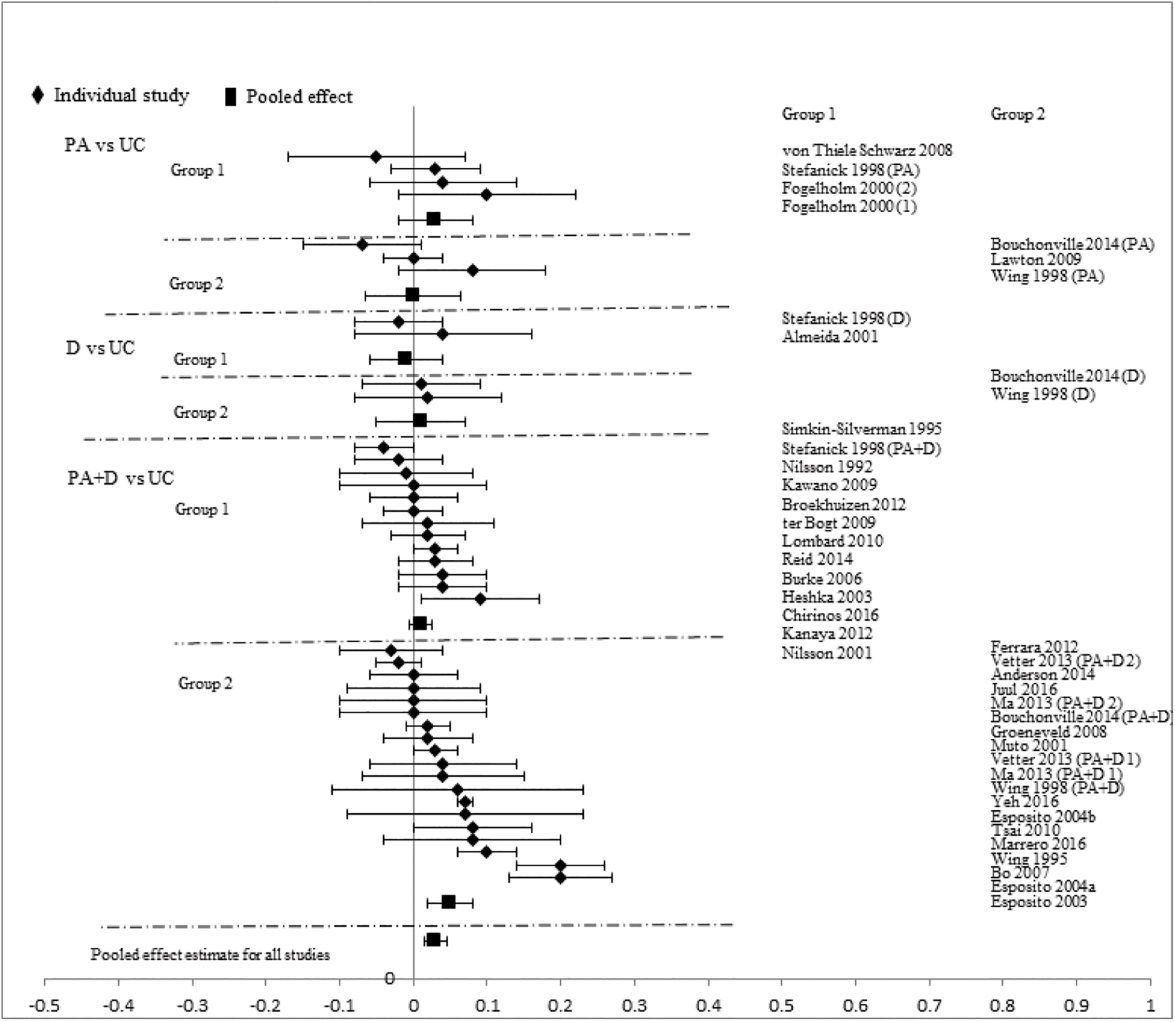
changes in high density lipoprotein cholesterol in the intervention versus usual care groups (mmol/L). Group 1: low-range glycemic group (FPG<5.5mmol/L or A1C <5.5%). Group 2: high-range glycemic group (FPG ≥5.5mmol/L or A1C ≥5.5%). D, diet, PA, physical activity. UC, usual care, vs, versus.

**Fig 7:**
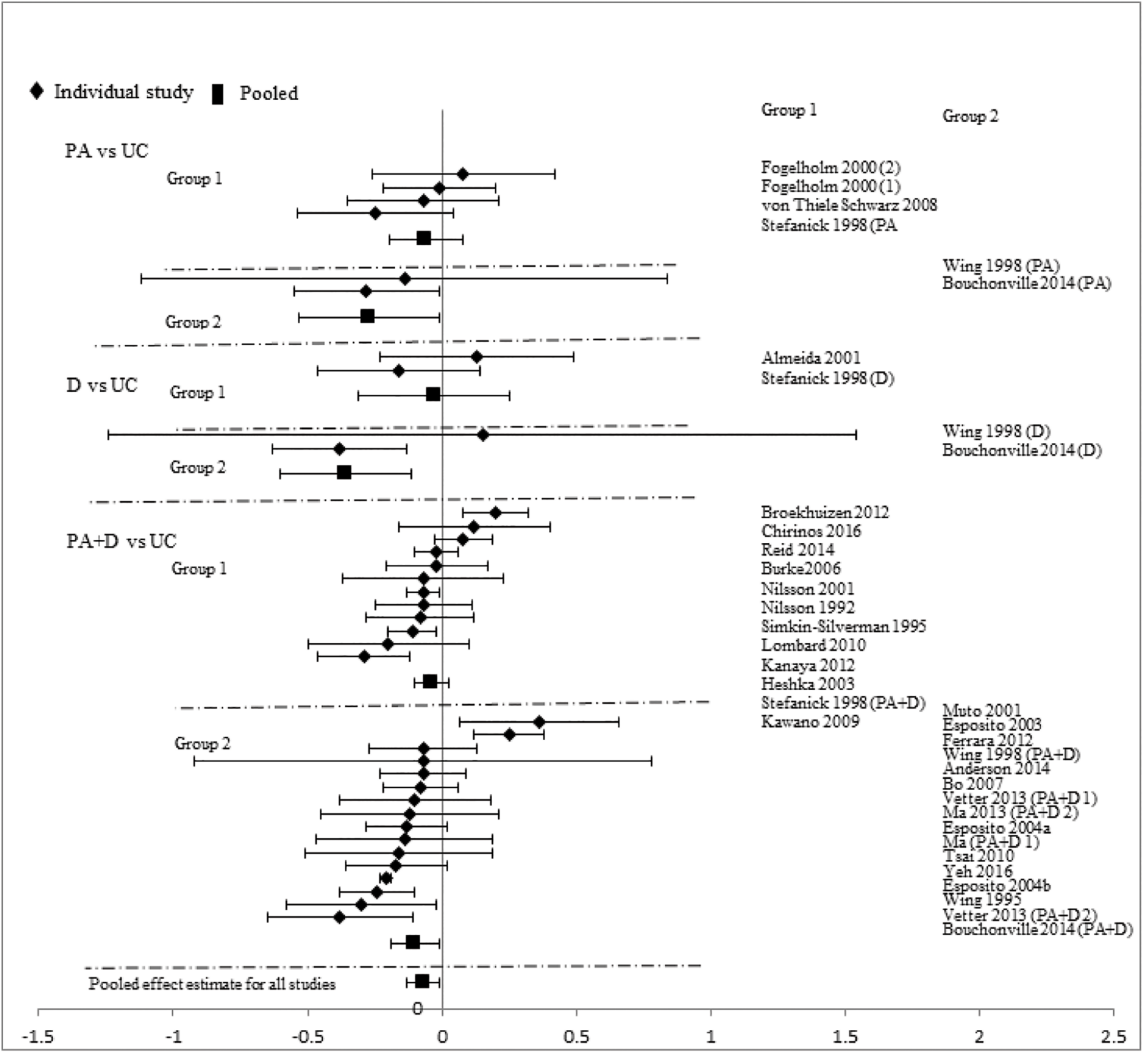
Changes in triglycerides in the intervention versus usual care groups (mmol/L). Group 1: low-range glycemic group (FPG<5.5mmol/L or A1C <5.5%). Group 2: high-range glycemic group (FPG ≥5.5mmol/L or A1C ≥5.5%). D, diet, PA, physical activity, UC, usual care, vs, versus.

Analyses stratified by intervention types showed that PA+D vs UC achieved the best incremental improvements in SBP (-2.29mmHg [-3.19, -1.40]), DBP (-1.66mmHg [-2.24, -1.09]), TC (-0.10mmol/L [-0.16, -0.05]), LDL-C (-0.08mmol/L [-0.14, -0.02]), HDL-C (0.03mmol/L [0.02, 0.05]), and TG (-0.07mmol/L [-0.13, -0.01]). D vs UC showed significant improvements in two categories: DBP (-2.28mmHg [-4.07, -0.49]), TC (-0.17mmol/L[-0.34, -0.01]); improvements in other measures did not reach statistical significance. Improvements with PA vs UC did not reach statistical significance in any category: SBP (-0.72mmHg [-1.89, 0.44]), DBP (-1.12mmHg [-2.34, 0.10]), TC (-0.02mmol/L [-0.09, 0.06]), LDL-C (-0.03mmol/L [-0.18, 0.12]), HDL-C (0.01mmol/L [-0.02, 0.04]), and TG (-0.10mmol/L [-0.22, 0.02]). Pooled effects of CVD risk reduction are presented in Figs 2–7.

### Comparison according to length of follow-up

In 34 studies or study arms with 12 months of follow-up, lifestyle interventions significantly improved all CVD risk factors: SBP (-2.07mmHg [-3.19, -0.95]), DBP (-1.62mmHg [-2.29, -0.95]), TC (-0.06mmol/L [-0.10, -0.01]), LDL-C (-0.08mmol/L [-0.13, -0.02]), HDL-C (0.02mmol/L [0.01, 0.03]), and TG (-0.08mmol/L [-0.14, -0.03]). For 7 studies or study arms with 13–23 months of follow-up, significant improvements were observed in four CVD risk factors: SBP (-1.73mmHg [-2.80, -0.65]), DBP (-1.25mmHg [-2.02, -0.48]), TC (-0.19mmol/L [-0.26, -0.11]), and LDL-C (-0.12mmol/L [-0.19, -0.05]). When the follow-up was ≥24 months (n = 14), significant improvements remained visible only for: SBP (-1.58mmHg [-2.71, -0.45]), DBP (-1.36mmHg [-2.30, -0.41]), and HDL-C (0.05mmol/L [0.02, 0.08]).

### Correlation between interventional effects on CVD risk reduction and glucose change and weight loss effect sizes

Findings from meta-regression analyses demonstrated that except for LDL-C category, Pearson’s correlation, r between CVD risk reduction effect sizes and glucose effect sizes ranged from 0.73 to 0.83 in SBP, DBP, TC, HDL-C, and TG, but r between CVD risk reduction effect sizes and baseline FPG were very low, only ranging from 0.26 to 0.44 in SBP, DBP, TC, HDL-C, and TG. The r between CVD risk reduction effect sizes and weight followed the same patterns: except for LDL-C category, r between CVD risk reduction effect sizes and weight loss effect sizes ranged from 0.51 to 0.75 in SBP, DBP, TC, HDL-C, and TG, but r between CVD risk reduction effect sizes and baseline weight were very low, only ranging from 0.02 to 0.30 in SBP, DBP, TC, HDL-C, and TG. Compared to weight loss, glucose response is a better indicator of the CVD risk factor response because the glucose response has a stronger correlation with the CVD risk factor response as r ranges showed above (Table 3).

**Table 3:** Correlation between CVD Risk Reduction and FPG and Weight.

CVD risk reduction		R		
Effect size	Baseline FPG	FPG effect size	Baseline weight	Weight loss effect size
SBP	0.32	0.752	0.068	0.506
DPB	0.259	0.728	0.023	0.58
TC	0.301	0.827	0.127	0.75
LDL-C	0.186	0.117	0.196	0.18
HDL-C	0.437	0.82	0.301	0.708
TG	0.38	0.82	0.172	0.707

Abbreviations: CVD: cardiovascular disease; DBP: diastolic blood pressure; FPG: fasting plasma glucose; HDL-C: high density cholesterol; LDL-C: low density cholesterol; SBP: systolic blood pressure; TC: total cholesterol; TG: triglycerides

## Discussion

In this review of the effectiveness of lifestyle interventions on the reduction of CVD risk factors among adults with low glycemic levels (below the IGT threshold), we found that lifestyle interventions, including physical activity, diet, and behavioral modification, can significantly improve CVD risk profiles, including SBP, DBP, TC, LDL-C, HDL-C, and TG. When stratified by glycemic levels, we found similar intervention effects between studies of participants with low vs high-range glycemic levels, except for TC and TG. Greater improvements were observed among studies with 12 months of follow-up than those with longer follow up, such that only SBP, DBP, and HDL-C improvements were sustained after 24 months. Studies that used a combined strategy of PA and D had the strongest effect on improving CVD profiles, followed by studies using D interventions only; studies only using a PA intervention strategy had the weakest effect. We have previously reported that multi-faceted interventions combining PA and D are effective in improving glucose regulation in populations with average low-range and high-range glucose levels.[125] The results of the present analyses suggest the effect of such interventions also applies to traditional biologic CVD risk factors.

Lifestyle interventional effects on CVD risk reduction observed in our studies among people without IGT or diabetes are consistent with those from the main trials of diabetes prevention among persons with IGT. For example, the US Diabetes Prevention Program (DPP) Study among people with IGT reported improvements in CVD profiles for all categories as measured by the mean differences between lifestyle intervention and placebo groups. The magnitude of improvements in CVD profiles in the DPP[126] in 1-year follow-up are consistent with those from our review (DPP vs this review: SBP, -2.50 vs -2.07 mmHg; DBP, -2.71 vs -1.62 mmol/L; TC, -0.06 vs -0.06 mmol/L; LDL-C, -0.02 vs -0.08 mmol/L; HDL-C, 0.01 vs 0.02 mmol/L; TG, -0.18 vs. -0.08 mmol/L, respectively). This comparison is also true for other major diabetes prevention trials (e.g., Finish Diabetes Prevention Study).[127]

Our findings may have important implications for decision makers in the areas of both diabetes and CVD primary prevention. Our meta-regression analyses indicated that the magnitude of improvements in CVD risk profiles is less correlated with baseline glucose level, but highly correlated with the effect sizes of glucose improvement. Meanwhile, the meta-regression analyses also indicated that the magnitude of improvements in CVD risk profiles is less correlated with baseline body weight, but highly correlated with the effect sizes of weight loss. We thus conclude that lifestyle interventions may provide important benefits across the full distribution of glycemic levels and body weight, including populations with glycemic levels below the IGT threshold, for both the low and high ranges of baseline FPG, and for populations with normal weight but with CVD risk factors. However, economic factors as well as the effectiveness of interventions influence decisions regarding the types of interventions provided to individuals with glycemic levels below the IGT threshold.[128,129] The cost-effectiveness of lifestyle interventions that can simultaneously reduce diabetes and CVD risk among individuals with glycemic levels below the IGT threshold should be examined.

Our findings demonstrate that lifestyle interventions, compared to UC, achieved improvement in both diabetes prevention and CVD risk reduction, and these improvements were not only statistically significant, but also have clinical relevance. Previous studies indicated that each 0.03 mmol/L increase in HDL-C is associated with the reduction of coronary heart disease risk by 2–3%,[130] and each 5 mmHg reduction in SBP and 2 mmHg reduction in DBP reduce stroke risk by 13% and 11.5%, respectively.[131] According to an epidemiology study, a 1% decrease in total cholesterol leads to a decrease in the incidence of coronary events by 2%.[132] One study also indicated that weight loss improved CVD profiles because each kilogram change in body weight was related to the change in the risk of coronary heart disease by 3.1%.[133]

Given that lifestyle intervention program participants in our reviewed studies usually achieved improvements in CVD across a full spectrum of outcomes simultaneously, the overall combined benefits brought by lifestyle interventions could be amplified. An estimation of overall effect on CVD risk would be helpful for our understanding the importance of interventional impact. Unfortunately, although there are several models available for CVD risk calculation (e.g., Framingham Risk Score,[134] and the ACC/AHA CVD risk calculator[135]), we are not aware of any available estimation model by which we can calculate the overall combined effect of changes of different individual risk factor. Further research and validation test, therefore, maybe needed for creating this model. If this kind model is available in the future, we can apply this model to our meta-analytic findings to estimate the overall combined effect of changes of different individual risk factor. For example, if a population, through lifestyle and behavior changes, achieved CVD risk reductions as much as showed in our meta-analyses, we can estimate the overall health benefits (e.g., how many CVD events can be prevented in the future). Despite this unavailability, the improvement in glucose regulation[125] coupled with our findings regarding the improvement in CVD risk reduction suggested that lifestyle interventions can achieve a comprehensive improvement goal as stated in AHA Special Report[17] of preventing CVD and diabetes simultaneously among persons with lower diabetes risk.

Strong evidence shows that PA programs have important independent effects on non-insulin-mediated glucose transport, markers of inflammation, insulin resistance, blood pressure, lipid profile, fitness, and improved lean-to-fat mass ratio.[136] Our findings suggest that these effects were more likely observed in studies using multi-component interventions, including PA, calorie restriction, and behavioral support but less so for PA-only interventions. This finding may be related to methodological shortcomings in exercise-only interventions such as low adherence, insufficient exercise volume or length of intervention. Previous studies suggest that it may take up to 2 years for a previously sedentary obese individual to attain enough volume of exercise to effectively reduce CVD risk factors, and individuals in unverified, out-patient interventions are less likely to engage in the prescribed amount of exercise.[137,138] However, we previously reported that exercise-only interventions in our included studies significantly reduced FPG and body weight[125] which in turn further prevented diabetes. Since PA-related improvements in glucose regulation and weight loss can lead to reductions in CVD risk profiles, potential indirect benefits should be taken into account when interpreting our findings.

Unhealthy lifestyle factors are related to the atherosclerotic process and these long-term exposures lead to the clinical manifestations of cardiovascular events.[139] A previous study also indicated that lifestyle changes, only in the long-term, are likely to lead to CVD risk factor reduction.[30] Our findings demonstrate that the effects of lifestyle changes on the reduction in CVD risk factors reached their highest point at 12 months of follow-up, then gradually decreased over time. This may reflect the fact that the longer-term intervention may be more effective on reducing CVD risks only if participates remain highly adherent to the intended interventions, which is seldom observed. It could be also true that using CVD mortality, rather than CVD risk reduction alone, to measure the long-term effect of lifestyle changes on CVD is more appropriate as the extended legacy findings of the Chinese Da Qing Study indicated.[140]

Because we used a comprehensive search strategy including all major medical databases, we found a large number of eligible studies. Pooled effects based on a large sample size provide more robust findings than those from any single study. Our review has some limitations as well. First, lifestyle interventions were used in heterogeneous settings, among different populations of varying ages, health status, and race/ethnicity background. While the main components of the lifestyle interventions were generally PA and D, each of the strategies had its own requirements in type, dose, intensity, and frequency. UC also had varying definitions among different comparison groups. Heterogeneity across studies was also reflected in the length of intervention, duration and follow-up, and number of sessions. However, our meta-regression analyses found no interactions between the between-group change in glycemic indicators and study-level characteristics. We also stratified our data syntheses by glycemic level, length of follow-up, and type of interventions, taking the heterogeneity among included studies into account. Second, although we stratified by level of glycemic risk at the study level, there was considerable heterogeneity within studies, and the nature of aggregated data prevented individual level classification by glucose level. As a result, there was likely considerable overlap in participant characteristics between low range and high range glycemic groups in our study, which may introduce some misclassification bias. Misclassification bias could be also introduced by usage of both FPG and A1C in our review to identify population with low glycemic risks. Although a previous study indicated that the agreement between FPG and A1C is high,[141] they are not equal with each other.[142] Because of this misclassification bias, some individuals identified as with low glycemic risks could actually have glucose metabolism abnormalities. Audiences need to be cautious when interpreting our findings.

## Conclusions

Our review is the first comprehensive examination of the impact of lifestyle interventions on risk for progression of dysglycemia and CVD risk reduction among persons below the IGT threshold. This systematic review suggests that lifestyle change is critical to both CVD risk reduction and diabetes prevention across the full spectrum of risk, complementing the major trials of diabetes prevention that focused on persons with IGT. This review also provides supportive evidences for designing strategies aimed at reducing CVD burden as delineated in the AHA Strategic Impact Goal through 2020 and Beyond.[17] Our findings demonstrated that among adults without IGT or diabetes, PA and D interventions, especially combined can significantly improve SBP, DBP, TC, LHL-C, HDL-C, and TG, in addition to glucose regulation and weight loss, and that these risk reductions may further prevent CVD events.

## Supporting information

S1 File Appendix A. Protocol-Study Protocol with Search Strategy.
Appendix B. PRISMA Checklist- Preferred Reporting Items for Systematic Reviews and Meta-Analyses checklist.
Table A. Intervention Characteristics.
Table B. Quality Assessment.
Table C. Lifestyle Interventional Effect: Meta-analyses Results in A Single Arm Model.
Table D. Intervention effect on FPG and percent weight: meta-analyses results.
(DOCX)
