# Supplementary material for: Effect of lifestyle interventions on cardiovascular risk factors among adults without impaired glucose tolerance or diabetes: A systematic review and meta-analysis
Source: PLoS One. 2017 May 11;12(5):e0176436. doi: 10.1371/journal.pone.0176436 (PMC5426619; doi:10.1371/journal.pone.0176436)
Supplement: S1 File — Appendix A. Protocol-Study Protocol with Search Strategy. Appendix B. PRISMA Checklist- Preferred Reporting Items for Systematic Reviews and Meta-Analyses checklist. Table A. Intervention Characteristics. Table B. Quality Assessment. Table C. Lifestyle Interventional Effect: Meta-analyses Results in A Single Arm Model. Table D. Intervention effect on FPG and percent weight: meta-analyses results. (DOCX) [file pone.0176436.s001.docx]

**Appendix A in S1 File. Study Protocol-** **Study Protocol with Search Strategy**

Study Protocol

Effect of Lifestyle Interventions on Cardiovascular Risk Factors among Adults without Impaired Glucose Tolerance or Diabetes: A Systematic Review and Meta-analysis

1. **Rationale for the study**

Structured lifestyle interventions can reduce diabetes incidence and cardiovascular disease (CVD) risk among persons with impaired glucose tolerance (IGT), but debate remains about whether they should be implemented among persons without IGT.

**B. Objective**

The objective of this study is to assess the effect of lifestyle interventions on CVD risk reduction among adults without IGT or diabetes.

**Primary research questions**

1. How lifestyle intervention strategies affect CVD risk reduction among adults without IGT or diabetes?

**Secondary research questions**

If these interventions have significant effects on CVD risk reduction, then analyses will be undertaken for the following secondary research questions:

1. What characteristics of those strategies correlate with the changes in CVD risk factors?
2. Whether lifestyle interventions focused on diet, PA or their combination have varying impact on CVD risk reduction?

**C.** **Criteria for considering studies for this review**

**Summary of inclusion criteria:**

1. Study design:
   1. All study designs that include a comparison group: either a contemporaneous comparison group (randomized controlled trial, controlled clinical trial, controlled before and after trial), or an interrupted time series or pre versus post design
   2. Follow-up period (including duration of the intervention) ≥12 months
2. Participants:
   1. Persons ≥18 years
   2. Any glycemic level without IGT or DM
   3. Any BMI or weight at baseline
3. Intervention:
   1. One of the stated goals of the intervention is intentional reduction of CVD risk factors
   2. Improve health in general, but measure CVD risk reduction as one of outcomes
   3. The intervention focuses primarily on the person with normal glucose, not on the provider
4. Outcomes:
   1. Systolic blood pressure (SBP), diastolic blood pressure (DBP), total cholesterol (TC), low density lipoprotein cholesterol (LDL-C), high density lipoprotein cholesterol (HDL-C), and triglycerides (TG)
   2. Weight loss, BMI, measured at both baseline and follow-up

**Summary of exclusion criteria:**

1. Study populations with IGT or diabetes
2. Provider-focused interventions

Details of the inclusion and exclusion criteria are provided below.

**1. Types of studies**

a. Trial design

The following types of study designs will be included in this review: randomized controlled trials, controlled clinical trials, controlled before and after trials, interrupted time series or pre versus post design. Ideally we would identify an adequate number of randomized, controlled trials and controlled clinical trials (quasi-randomized studies) and use these for our pooled estimates and regression analyses. However, if we fail to identify these, we will also consider other types of comparative design studies.

Observational studies yield important information on intervention effectiveness. Classic randomized controlled trials emphasize efficacy, to the exclusion of factors influencing effectiveness such as adoption (the proportion and representativeness of settings that adopt a policy or program), reach (the percentage and risk characteristics of persons who receive or are affected by a program), and institutionalization.^1^ Thus, randomized controlled trials generally have limited applicability as populations and settings are narrowly defined. In this review we would like to determine the effectiveness of interventions for a broad range of populations and settings.

We recognize the potential for bias from confounding and secular trends in studies without randomization. We will assess the quality of these studies and if they are used in the analyses, they will be analyzed separately from the randomized trials in the meta-analysis. We will discuss potential sources of bias and indicate how they might have affected results.

Studies with a follow-up period of 12 months or greater will be included in this review. We define follow-up period from the time of randomization (or for non-randomized trials from the time of entrance into the study) until the last measurement in the study. The intervention itself may be of any duration. In order for lifestyle interventions to produce long-term effects on health outcomes such as IGT incidence, the demonstration of long-term improvement of glucose tolerance is needed. We exclude interventions for which follow-up is less than 12 months.

When we synthesize data from the included studies and conduct a meta-analysis, we will take into account the duration of follow-up, and stratify the pooling estimates according to duration of follow-up.

1. **Types of participants**

Studies will include adults 18 years of age or greater without IGT. If participants are a mixture of people with normal glucose and IGT, or diabetes, we only include these studies in which outcomes data have been broken down between NGT and other groups.

Study participants can be of any weight or BMI at baseline; they do not have to be overweight or obese.

**3. Types of interventions**

The interventions examined in this review focus on the patient, rather than the provider or health care system. Interventions which focus on changing provider behavior will be excluded, even when outcomes are measured in the patient (e.g., an intervention to educate providers about counseling, with patients’ CVD risk factors as the main outcome).

Interventions included in the review must have change in CVD risk factors as one of the primary stated goals of the intervention.

The types of interventions included in this review are described below. These categories are not intended to be mutually exclusive, but rather to describe the broad interventions categories that we are including in this review. Our analytic approach is to examine a broad array of variables describing each intervention, permitting us to correlate outcomes with individual and combined intervention characteristics.

**a. Behavioral strategies**

Behavioral strategies are based on behavioral and learning principles, and provide tools for overcoming barriers to improving and optimizing diet and physical activity levels.

Behavioral strategies include one or more of the following interventions:

i. Education

Educational interventions focus on the transfer of information from educator to patient. The intervention might be implemented in a didactic manner where the participant simply attends to the educator or written material, or in a collaborative fashion involving patient participation.

ii. Cognitive-behavioral therapy

Cognitive therapy is concerned with the modification of behavior through the influence of thought processes. The principles include the modification of behavior patterns, new adaptive learning, problem solving, and the establishment of a collaborative relationship with therapist. Cognitive therapy may be performed as part of a treatment package called behavioral therapy, which may include one or more of the following strategies to overcome barriers to positive changes in behaviors: self-monitoring (weight, diet, or physical activity), stimulus control, reinforcement, problem solving, stress management, contingency management, cognitive restructuring (the promotion of more positive thoughts about bad eating), goal setting, inoculation training, relaxation skills, meditation, and relapse prevention training.

iii. Social support

These interventions focus on providing or promoting social support to achieve changes in glucose regulations. This support might be provided by family members, friends, or the community.

iv. Other

Several other types of behavioral interventions have been utilized, including self-monitoring of blood glucose for the purposes of glucose regulations and psychotherapy.

**b. Dietary programs**

Dietary programs involve providing recommendations and/or material support for achieving a specific dietary regime where the direct goal is weight loss or weight maintenance, and the final goal is changes in glucose regulations. All types of dietary programs initiated for the purpose of weight loss or control will be examined in this review.

**c. Physical activity programs**

Trials of these programs are included if one of the primary goals of the program is to achieve changes in glycemic indicators by increased physical activity. Where it is simply stated that participants were advised to increase their level of exercise, with no further details, this will not be considered a physical activity program, unless the authors can provide further details.

**d. Combination interventions**

Studies will be included that examine the combined effect of two or more of the interventions discussed above.

**Excluded interventions:**

We will exclude pharmacologic therapy for the purpose of glucose tolerance improvement as the intervention has very different mechanisms of action. We will also exclude herbal remedies and dietary supplements. Provider-focused interventions will be excluded, as mentioned above.

**Examination of intervention characteristics**

A number of key dimensions of lifestyle interventions have been suggested,^2-3^ and these data will be abstracted from included studies and analyzed. These include intervention characteristics, mode of delivery of the intervention, and the specific intervention components. These characteristics will also be gathered for the comparison group (if any).

**4. Types of comparison interventions**

Studies that involve a comparison group will be included regardless of the nature of the comparison intervention. We are including studies with a range of comparison groups as we want to determine which interventions are more effective than others.

The comparison group could receive:

a. Placebo

No intervention

Usual care

1. The same intervention at a different intensity (frequency, duration, time frame for delivery)
2. Any other glucose regulation intervention: behavioral strategy, dietary program, physical activity program, other

**5. Types of outcome measures**

a. Main outcome measures:

Serum lipids

Total cholesterol

Triglycerides

LDL-C

HDL-C

Blood pressure

Systolic blood pressure

Diastolic blood pressureGlycemic indicators

Glycemic indicators

FPG change

A1C change

Fasting insulin change

Homeostasis model assessment-estimated insulin resistance (HOMA-IR)

Weight

BMI

Abdominal fat distribution: waist hip ratio, waist circumference

Quality of life

b. Additional outcome measures

Morbidity

Incidence of IGT, hypertension

Other

Lifestyle changes

Dietary intake: calories, fat, fiber, carbohydrate, protein

Physical activity, smoking status

c. Timing of outcome assessment

Studies with a follow-up period of 12 months or greater will be included in this review. In order for CVD risk reduction to produce long-term effects on health outcomes such as decrease of CVD events, the demonstration of long-term changes in CVD risk factors are needed. Therefore we exclude interventions for which follow-up is less than 12 months.

**D. Search strategies for identification of studies**

A number of electronic databases will be examined. The titles and abstracts obtained from searches of electronic databases will be examined, and potentially relevant full-text articles will be requested. There will be no language restrictions on our searches. Two people will screen the Medline titles and abstracts. Conference proceedings and abstracts will be included in the review, but not in the analysis because there is insufficient detail available in these to evaluate the intervention and the quality of the study. These will be summarized in narrative form, and presented as potentially important studies that may appear in future in the literature. Dissertations will be excluded, as these are difficult to locate in full text, and will likely yield few additional reports.

**1. Databases:**

The following electronic databases will be searched:

Cochrane Library

Cochrane Controlled Trials Register (including DARE)

MEDLINE (1966*) (includes Healthstar)

EMBASE

CINAHL (1982*)

Web of Science (1981*)

Psychoinfo (1967*)

Searches will commence at the start date of the database (given in parentheses above with (*), or in the year indicated).

**2. Other search strategies**

Potential missing and unpublished studies will also be sought by contacting experts in the field. We will review the reference lists of all relevant review articles and of the studies included in the review.

Additional key words of relevance may be identified during any of the electronic or other searches. If this is the case, electronic search strategies will be modified to incorporate these terms.

1. **Search strategies**

**Example 1 (Publications in MEDLINE in English):**

Database(s): **Ovid MEDLINE(R) and Ovid OLDMEDLINE(R)** 1946 to January Week 3 2013 (Updated search conducted through April 17, 2015)
Search Strategy:

| **#** | **Searches** | **Results** |
| --- | --- | --- |
| 1 | glucose tolerance test/ | 27601 |
| 2 | glucose intolerance/pc (prevention & control) | 199 |
| 3 | blood glucose/an (analysis) | 45132 |
| 4 | blood glucose/me (metabolism) | 56949 |
| 5 | prediabetic state/pc | 109 |
| 6 | (normal adj3 "glucose toleran*").tw. | 3909 |
| 7 | "normal glucose regulation".tw. | 85 |
| 8 | "normal fasting glucose".tw. | 305 |
| 9 | "baseline glucose tolerance".tw. | 20 |
| 10 | "glucose tolerance status*".tw. | 322 |
| 11 | (improve* adj3 "glucose tolerance").tw. | 1883 |
| 12 | (prevent* adj3 ("impaired glucose" or "impaired fasting")).tw. | 28 |
| 13 | (prevent* adj3 (igt or ifg)).tw. | 25 |
| 14 | or/1-13 | 117255 |
| 15 | weight loss/ | 21081 |
| 16 | exp diet therapy/ | 38294 |
| 17 | food habits/ | 18465 |
| 18 | feeding behavior/ | 35111 |
| 19 | exp diet/ | 177116 |
| 20 | exp exercise/ | 98460 |
| 21 | motor activity/ | 69067 |
| 22 | "physical activity".tw. | 43001 |
| 23 | "Physical Education and Training"/ | 11350 |
| 24 | Physical Fitness/ | 20255 |
| 25 | exp exercise therapy/ | 26459 |
| 26 | resistance training/ | 2258 |
| 27 | exp Sports/ | 101425 |
| 28 | strength train*.tw. | 2432 |
| 29 | life style/ | 38906 |
| 30 | health education/ | 49814 |
| 31 | health promotion/ | 47471 |
| 32 | "patient education as topic"/ | 65937 |
| 33 | health behavior/ | 29062 |
| 34 | risk reduction behavior/ | 5828 |
| 35 | "Tobacco Use Cessation"/ | 608 |
| 36 | smoking cessation/ | 17720 |
| 37 | smoking/ | 110147 |
| 38 | "tobacco use disorder"/ | 7043 |
| 39 | cognitive therapy/ | 13481 |
| 40 | behavior therapy/ | 22365 |
| 41 | counseling/ | 26000 |
| 42 | or/15-41 | 807160 |
| 43 | 14 and 42 | 21510 |
| 44 | limit 43 to english language | 19806 |
| 45 | 44 not animals/ | 14334 |
| 46 | 44 and (humans/ and animals/) | 639 |
| 47 | 45 or 46 | 14973 |
| 48 | 47 and (random* or trial* or placebo*).mp. (any field) | 4521 |

**Example 2 (Publications in MEDLINE in non-English):**

Database(s): **Ovid MEDLINE(R) In-Process & Other Non-Indexed Citations and Ovid MEDLINE(R)** 1946 to Present (Updated search conducted through April 17, 2015)
Search Strategy:

| **#** | **Searches** | **Results** |
| --- | --- | --- |
| 1 | glucose tolerance test/ | 28010 |
| 2 | glucose intolerance/pc | 220 |
| 3 | blood glucose/an | 45682 |
| 4 | blood glucose/me | 58135 |
| 5 | prediabetic state/pc | 118 |
| 6 | (normal adj3 "glucose toleran*").tw. | 4180 |
| 7 | "normal glucose regulation".tw. | 90 |
| 8 | "normal fasting glucose".tw. | 336 |
| 9 | "baseline glucose tolerance".tw. | 20 |
| 10 | "glucose tolerance status*".tw. | 351 |
| 11 | (improve* adj3 "glucose tolerance").tw. | 2145 |
| 12 | (prevent* adj3 ("impaired glucose" or "impaired fasting")).tw. | 29 |
| 13 | (prevent* adj3 (igt or ifg)).tw. | 27 |
| 14 | or/1-13 | 119580 |
| 15 | weight loss/ | 21756 |
| 16 | exp diet therapy/ | 38948 |
| 17 | food habits/ | 19053 |
| 18 | feeding behavior/ | 35928 |
| 19 | exp diet/ | 180518 |
| 20 | exp exercise/ | 101022 |
| 21 | motor activity/ | 70976 |
| 22 | "physical activity".tw. | 48797 |
| 23 | "Physical Education and Training"/ | 11401 |
| 24 | Physical Fitness/ | 20533 |
| 25 | exp exercise therapy/ | 27104 |
| 26 | resistance training/ | 2439 |
| 27 | exp Sports/ | 103227 |
| 28 | strength train*.tw. | 2704 |
| 29 | life style/ | 39763 |
| 30 | health education/ | 50476 |
| 31 | health promotion/ | 48753 |
| 32 | "patient education as topic"/ | 66739 |
| 33 | health behavior/ | 30176 |
| 34 | risk reduction behavior/ | 6187 |
| 35 | "Tobacco Use Cessation"/ | 630 |
| 36 | smoking cessation/ | 18273 |
| 37 | smoking/ | 112159 |
| 38 | "tobacco use disorder"/ | 7296 |
| 39 | cognitive therapy/ | 13994 |
| 40 | behavior therapy/ | 22682 |
| 41 | counseling/ | 26492 |
| 42 | or/15-41 | 826852 |
| 43 | 14 and 42 | 22052 |
| 44 | limit 43 to english language | 20339 |
| 45 | 44 not animals/ | 14702 |
| 46 | 44 and (humans/ and animals/) | 666 |
| 47 | 45 or 46 | 15368 |
| 48 | randomized controlled trial.pt. | 346677 |
| 49 | controlled clinical trial.pt. | 85722 |
| 50 | randomized.ab. | 264499 |
| 51 | placebo.ab. | 143200 |
| 52 | clinical trials as topic/ | 163888 |
| 53 | randomly.ab. | 192601 |
| 54 | trial.ti. | 112945 |
| 55 | or/48-54 | 833571 |
| 56 | 47 and 55 | 3453 |
| 57 | 47 and meta-analysis.mp. | 118 |
| 58 | 47 and "systematic review".ti. | 41 |
| 59 | 57 or 58 | 135 |
| 60 | 56 not 59 | 3404 |
| 61 | 56 or 59 | 3539 |
| 62 | remove duplicates from 61 | 3458 |
| 63 | follow-up studies/ | 464828 |
| 64 | 47 and 63 | 837 |
| 65 | Hemoglobin A, Glycosylated/ | 20630 |
| 66 | 65 and 42 and (55 or 63 or meta-analysis.mp. or "systematic review".ti.) | 1480 |
| 67 | HbA1c.tw. | 12093 |
| 68 | 67 and 42 and (55 or 63 or meta-analysis.mp. or "systematic review".ti.) | 841 |
| 69 | 64 or 66 or 68 | 2413 |
| 70 | limit 69 to english language | 2329 |
| 71 | 70 not animals/ | 2305 |
| 72 | 70 and animals/ and humans/ | 14 |
| 73 | 71 or 72 | 2319 |
| 74 | 73 not 61 | 1452 |
| 75 | remove duplicates from 74 | 1415 |
| 76 | 43 not 44 | 1713 |
| 77 | 69 not 70 | 84 |
| 78 | 76 or 77 | 1765 |
| 79 | 78 and 55 | 158 |
| 80 | 78 and (meta-analysis.mp. or "systematic review".ti. or follow-up studies/) | 93 |
| 81 | 79 or 80 | 234 |
| 82 | 81 not animals/ | 225 |
| 83 | 81 and animals/ and humans/ | 3 |
| 84 | 82 or 83 | 228 |

**4. Handsearches**

The following journals, felt to be of high topic relevance will be hand-searched from 1980 to the present:

Circulation

International Journal of Obesity

American Journal of Clinical Nutrition

Journal of the American Dietetic Association

**E. Methods of the review**

**1. Trials selection**

Two independent reviewers will identify potentially relevant studies by reviewing titles and abstracts retrieved from electronic searches. For studies identified as potentially relevant, the full text article will be retrieved. Two persons (one content expert, one not) will review potential studies to see if they fulfill inclusion criteria. There will be no blinding of these reviewers to study author and affiliation. If there are disagreements between the reviewers, they will be discussed and consensus achieved. Studies excluded at this point or thereafter will be recorded in the bibliographic database, noting the reason for exclusion.

**2. Quality assessment**

An assessment of internal validity will be performed by two persons for each study and inter rater agreement measured with a kappa statistic. For randomized controlled trials, the component assessment method of Cochrane will be used,^4^ and quality scoring will not be performed. The key domains that determine internal validity will be assessed as ‘met’ or ‘unmet’ or ‘unclear’ for each study. These domains are: ^4^

- method of randomization

- allocation concealment (for RCTs only)
- attrition (>30% from baseline at one year follow-up and for each follow-up interval thereafter)
- blinding of the assessor for outcomes measured directly (in-person) by the researcher (e.g., weight or quality of life). Since blinding of the participant and provider is impossible in lifestyle and behavioral interventions, we will only consider blinding of the assessor.
- similarity at baseline of the groups being compared
- performance bias (likely contamination of the comparison group or cointervention to either group being compared)
- consistency of the data presented

The risk of bias will be then assessed as low (all criteria are met), moderate (one or more criteria only partly met), or high (one or more criteria are not met).

For studies that are not randomized controlled trials, the Newcastle-Ottawa Quality Assessment Scale for cohort studies will be used. This scale incorporates representativeness of the exposed cohort, selection of the non-exposed cohort, ascertainment of exposure, comparability of groups, potential confounders, attrition, and detection bias. For studies with a pre vs. post design or interrupted time series, a modified Newcastle-Ottawa Scale will also be used, with questions omitted about comparability and selection of the non-exposed cohort.

Studies will not be excluded on the basis of poor quality. A sensitivity analysis will be performed to compare results between studies of high and low quality.

There will be no blinding of reviewers of quality assessment to study authors, institution, and journal as there is evidence that little benefit is achieved with blinded assessments.^5^

The applicability of each study will be noted with respect to intervention, population, and setting. An overall subjective assessment of applicability of the body of evidence for each subgroup with a sufficient number of studies will be made using the categories of applicability developed by *the Guide to Community Preventive Services.*^6^  These categories indicate whether the existing body of literature is: 1) likely to be applicable across a broad range of settings, populations, or intervention characteristics, 2) likely to be applicable across a broad range of settings, populations, or intervention characteristics assuming that the intervention is appropriately tailored to the population of interest, 3) applicable to specific populations or settings (with a list provided) with broader applicability uncertain, and 4) applicable only the specific populations or settings (with a list provided). Although these categories are subjectively determined, we feel that a narrative summary of potential applicability will be of use to policy- and other decision-makers.

**3. Data abstraction process**

For studies that fulfill inclusion criteria, two reviewers will abstract the relevant data using a standardized template in an Access database. If there are specific elements of the abstraction template for which there are consistent disagreements between the two reviewers, the template and the instructions for that element will be reviewed and modifications made as indicated. Disagreements will be resolved by a consensus process with a third reviewer, referring back to the original article.

Extraction of data will not be blinded, as there is no evidence that blinding of this process decreases bias in the conduct of systematic reviews and meta-analyses.^5, 7^ We will attempt to contact the authors for missing data or when we need clarification of the data presented.

**4. Data to be extracted**

a. Participant characteristics

- age

- initial weight
- BMI

- race/ethnicity

- presence of one of: IGT, or type 2 diabetes if mixture of population
- diagnostic criteria specified for IGT, or type 2 diabetes
- sex
- baseline DBP, SBP, TC, LDL-C, HDL-C, TG, fasting blood glucose, A1C, FI, HOMA-IR
- comorbidities: hypertension, hyperlipidemia, cardiovascular disease, arthritis, cancer
- disability status
- smoking status

b. Intervention setting

- country
- type of health care setting (academic center, community clinic, solo or small group practice, other)
- community-based (school, work-site, community center)
- urban, rural
- in-patient, out-patient

c. Intervention characteristics

- tailoring based on ethnicity or other group characteristics
- tailoring based on the individual
- stated goals of the intervention: changes in glycemic indicators, change in glucose regulations, intentional weight loss, and combination weight loss and control (maintenance)
- theory-based or not
- commercial program
- financial (or monetary equivalent) incentives

d. Mode of delivery of the intervention

These characteristics will also be gathered for the comparison group (if any).

If the study combines a nutrition, physical activity intervention, then the following characteristics will be noted for both components.

- group, individual, combined
- didactic vs. collaborative interventions
- time frame over which the intervention was delivered
- number of participant contacts (specify individual or group)
- duration of each participant contact
- medium: in-person, telephone, electronic interactive with provider (internet, telemedicine), computer-based, written, mailed
- who delivers the intervention: diabetes educator, nurse, dietician, physician, physical activity trainer, lay health care worker, other
- family involvement
- physician monitoring

e. Intervention components

If the study combines a nutrition, physical activity intervention, then the following characteristics will be noted for both components

- single vs. multicomponent
- physical activity program
- dietary program
- behavioral intervention
- family-focused intervention, e.g., spouse involvement
- social support intervention
- community support
- smoking cessation

g. Physical activity interventions

- supervised vs home
- number of times a week
- number of minutes a week
- intensity: % VO_2_ max; % maximal heart rate; low, moderate, vigorous
- aerobic training, strength training, flexibility

h. Dietary programs

- mean intake: calories, carbohydrate, fiber, fat, protein
- main dietary regime:

very low calorie diets (VLCD) (<800 kcal/d)

low calorie diets (800-1500 kcal/d)

500-600 kcal. deficit diets or low fat

protein-sparing diet (<40g carbohydrate/d)

high fiber diet

- Other dietary program characteristics
- meal replacement diet
- sodium restriction

i. Behavioral interventions

- specific behavioral strategy: self-monitoring, stress management, stimulus control, problem solving, contingency management, cognitive restructuring, goal setting, inoculation training, relaxation skills, meditation, relapse prevention training, reinforcement

- therapist contact vs peer support

- underlying model: social learning theory, behavioral model, social psychology, health education

j. Study design characteristics

- study design: RCT, non-randomized trial, observational study with concurrent comparison group, pre vs post
- sample size
- follow-up interval from randomization or first intervention contact
- follow-up interval from last intervention contact
- date of publication
- run-in period or no; if yes, how long

k. Quality Assessment (internal validity)

- blinding of participants to the intervention; if yes, specify method
- blinding of assessor for subjective outcome measures (e.g., in-person assessment of quality of life); if yes, specify method
- blinding of the care provider
- unit of randomization
- baseline comparability of study groups
- identical treatment of groups being compared
- definition of inclusion criteria
- definition of exclusion criteria
- allocation concealment adequate
- withdrawals, drop-outs, and losses to follow-up: intervention, control, total
- likely contamination of the control group or cointervention to either comparison group
- consistency of the data presented
- intention-to-treat analysis
- last-measure-carried-forward utilized
- power calculation performed

m. Analytic approach

- intention-to-treat or no
- last-measure-carried-forward, or no
- power calculation performed

n. Compliance with intervention regime

- % of visits attended: intervention, control, total group
- % self-monitoring (weight, dietary intake, physical activity)

o. Outcomes

See C5 above for a list of outcomes. For continuous outcomes we will extract for each study group the baseline sample size, pre and post intervention mean and measure of dispersion (SD [standard deviation], standard error of the mean (SEM), or 95% confidence interval) for the intervention and comparison groups. If the post intervention measures of dispersion are not available, they will be assumed to be the same as the pre intervention measure. When necessary, mean and SD will be approximated from figures using an image scanner to optimize resolution. For dichotomous variables (e.g., mortality) the number of participants, person-years, and the number of events will be extracted for each group in the study.

**5. Data analysis**

**a. Exploratory data analysis**

Funnel plots will be used in exploratory data analysis to assess for the potential existence of small sample bias. There are a number of explanations for the asymmetry of a funnel plot, including true heterogeneity of effect with respect to study size, poor methodological design of small studies, and publication bias. Thus this exploratory data tool may be misleading and we will not place undue emphasis on this tool.

**b. Statistical pooling**

If data are available which are sufficiently similar with respect to interventions and outcomes, and of sufficient quantity, statistical analyses will be performed using Review Manager software (version 5.1; Copenhagen, Denmark).

For continuous variables reported in the same scale, we will calculate weighted mean differences. The mean difference between the intervention and control group (delta) for each individual study is equal to (delta I – delta C), where (delta I) and (delta C) are the absolute differences in outcome between each follow-up and the baseline measure for each study group. The estimate of variance of (delta I) and (delta C) will be calculated from the outcome measures in each study group using the formula V_pre_+ V_post_ - 2*r*(SE_pre_*SE_post_), where V_pre_ is the variance of the mean baseline outcome, V_post_ is the variance of the mean follow-up outcome, *r* is the correlation between the baseline and follow-up values, and SE_pre_ and SE_post_ are the standard errors of the baseline and follow-up groups, respectively. The variance of (delta) will then be calculated as the sum of the variance of (delta I) and the variance of (delta C). It is unlikely that studies will report *r*, and its true value is unknown, a sensitivity analysis will be performed using values of 0.25, 0.5 and 0.75.

When results for continuous outcomes are presented on different scales (e.g., quality-of-life) we will use standardized mean differences or we will develop dichotomous variables. The effect size for dichotomous data will be expressed in terms of relative risk.

**c. Regression analyses**

Data will be pooled using the random effects model, with the DerSimonian and Laird formula for calculating between-study variance.^8^ Each study will be weighted by the inverse of the study variance. Heterogeneity between trial results will be tested for using a standard chi-squared test and a significance level of alpha=0.1, in view of the low power of such tests. If heterogeneity is found, we will attempt to determine potential reasons for this by examining individual study characteristics and those of subgroups of the main body of evidence. If heterogeneity is felt to be too great to meaningfully pool the results in a quantitative manner, the results will be presented in a narrative fashion.

We will perform a meta-regression to determine if various intervention and study population characteristics affect outcomes. The meta-regression will also be weighted by the inverse of the variance of delta. Interaction terms will be examined for all models. SPSS will be used to perform the meta-regression (version 20.0, Armonk, NY: IBM Corp.).

The following variables will be examined in the meta-regression model:

1. Intervention characteristics:

Total contact time (=number of contacts multiplied by the duration of each contact)

Number of contacts

Mean age,

Follow-up duration,

Duration of the intervention,

Attrition, and

Year of publication

**6. Subgroup analyses**

If the review demonstrates a significant effect on glucose regulation, and if the amount of data permit, subgroup analyses will be conducted according to the following:

Care delivered to the comparison group

**-** Usual care

- The same intervention at a different intensity (frequency, duration, time frame for delivery)
- Any other dietary program, physical activity program, other

Demographic characteristics

- Fast plasma glucose
- overweight (25.0<=BMI<30.0) , obese (BMI >30.0), normal weight (BMI <25.0) at baseline
- age: young (<40y), middle-aged (40-65y), old (>65y)
- sex
- race/ethnicity: white, African American, Hispanic, Asian, other
- presence of comorbidities: yes or no

Design

- study design: RCT or controlled clinical trial; controlled before-and-after; pre versus post or time series

**6. Sensitivity analyses**

The effect of internal validity upon study results will be examined with a sensitivity analysis. Studies will be categorized as having 1) low risk of bias, 2) moderate risk of bias, and 3) high risk of bias depending on the presence of selection, detection, performance and attrition bias.^9^ We will perform a sensitivity analysis to determine if the overall results differ from the results using studies with low risk of bias.

Sensitivity analyses will also be performed based on:

1. Study quality: attrition ≥30%; adequate allocation concealment or not (applies to RCTs only), categorization as high, moderate, or low quality

2. Model for meta-analysis: Fixed effects versus random effects

3. Missing values: studies with values imputed for measures of variation; studies with all values reported

4. Use of last-outcome-carried-forward techniques

5. Language of publication: English; all languages including English

6. Publication status: all studies; only published studies

**Potential conflict of interest**

None identified

**Cover sheet**

**Sources of support**

Centers for Disease Control and Prevention, Atlanta, GA

**Referenc**e **List**

1. Glasgow R, Vogt T, Boles S. Evaluating the public health impact of health promotion interventions: the RE-AIM framework. *AJPH* 1999;89:1322-1327.
2. Wing RR. Behavioral treatment of obesity: its application to type II diabetes. *Diabetes Care* 1993;16:193-199.
3. Wing RR, Gorin A, Tate D. Strategies for changing eating and exercise behavior, in Bowman BA and Russell RM (eds), Present Knowledge in Nutrition: Washington, DC, ILSI Press, 2001. p. 650-660.
4. Higgins JPT, Green SE. Cochrane Handbook for Systematic Reviews of Interventions. [Version 5.1.0 [updated March 2011]]. 2011. The Cochrane Collaboration 2012.
5. Berlin J, Miles C, Crigilano M, Conill A, Goldmann D, Horowitz D, Jones F, Hanchk N, Williams S. Does blinding of readers affect the results of meta-analyses? Results of a randomized trial. *Online Journal of Current Clinical Trials* Document no. 205. 1997.
6. Task Force on Community Preventive Services. Introducing the Guide to Community Preventive Services: methods, first recommendations and expert commentary. *AJPM* 2000;18(suppl 1):1-142.
7. Irwig L, Toteson A, Gatsonis C, Lau J, Colditz G, Chalmers T, Mosteller F. Guidelines for meta-analyses evaluating diagnostic tests. *Ann Intern Med* 1994;120:667-676.
8. DerSimonian R, Laird N. Meta-analysis in clinical trials. *Controlled Clinical Trials* 1954;7:177-188.
9. Clarke M, Oxman AD. Cochrane reviewers handbook (updated October 2001), Oxford, Update Software, 2001.

**Appendix B in S1 File. PRISMA Checklist-** **Preferred Reporting Items for Systematic Reviews and Meta-Analyses checklist**

|  | | |  |  |  | |  |  |
| --- | --- | --- | --- | --- | --- | --- | --- | --- |
| **Section/topic** | | **#** | **Checklist item** | | | | **Reported on page #** | |
| **TITLE** | | | | | | |  | |
| Title | | 1 | Identify the report as a systematic review, meta-analysis, or both. | | | | 1 | |
| **ABSTRACT** | | | | | | |  | |
| Structured summary | | 2 | Provide a structured summary including, as applicable: background; objectives; data sources; study eligibility criteria, participants, and interventions; study appraisal and synthesis methods; results; limitations; conclusions and implications of key findings; systematic review registration number. | | | | 3-4 | |
| **INTRODUCTION** | | | | | | |  | |
| Rationale | | 3 | Describe the rationale for the review in the context of what is already known. | | | | 5-6 | |
| Objectives | | 4 | Provide an explicit statement of questions being addressed with reference to participants, interventions, comparisons, outcomes, and study design (PICOS). | | | | 6-7 | |
| **METHODS** | | | | | | |  | |
| Protocol and registration | | 5 | Indicate if a review protocol exists, if and where it can be accessed (e.g., Web address), and, if available, provide registration information including registration number. | | | | 7 | |
| Eligibility criteria | | 6 | Specify study characteristics (e.g., PICOS, length of follow-up) and report characteristics (e.g., years considered, language, publication status) used as criteria for eligibility, giving rationale. | | | | 7-8 | |
| Information sources | | 7 | Describe all information sources (e.g., databases with dates of coverage, contact with study authors to identify additional studies) in the search and date last searched. | | | | 7 | |
| Search | | 8 | Present full electronic search strategy for at least one database, including any limits used, such that it could be repeated. | | | | 7 | |
| Study selection | | 9 | State the process for selecting studies (i.e., screening, eligibility, included in systematic review, and, if applicable, included in the meta-analysis). | | | | 7-8 | |
| Data collection process | | 10 | Describe method of data extraction from reports (e.g., piloted forms, independently, in duplicate) and any processes for obtaining and confirming data from investigators. | | | | 8 | |
| Data items | | 11 | List and define all variables for which data were sought (e.g., PICOS, funding sources) and any assumptions and simplifications made. | | | | 7-8 | |
| Risk of bias in individual studies | | 12 | Describe methods used for assessing risk of bias of individual studies (including specification of whether this was done at the study or outcome level), and how this information is to be used in any data synthesis. | | | | 8-9 | |
| Summary measures | | 13 | State the principal summary measures (e.g., risk ratio, difference in means). | | | | 9-10 | |
| Synthesis of results | | 14 | Describe the methods of handling data and combining results of studies, if done, including measures of consistency (e.g., I^2^) for each meta-analysis. | | | | 9-10 | |

Page 1 of 2

| **Section/topic** | **#** | **Checklist item** | **Reported on page #** |
| --- | --- | --- | --- |
| Risk of bias across studies | 15 | Specify any assessment of risk of bias that may affect the cumulative evidence (e.g., publication bias, selective reporting within studies). | 8-9 |
| Additional analyses | 16 | Describe methods of additional analyses (e.g., sensitivity or subgroup analyses, meta-regression), if done, indicating which were pre-specified. | 9-10 |
| **RESULTS** | | |  |
| Study selection | 17 | Give numbers of studies screened, assessed for eligibility, and included in the review, with reasons for exclusions at each stage, ideally with a flow diagram. | 10 |
| Study characteristics | 18 | For each study, present characteristics for which data were extracted (e.g., study size, PICOS, follow-up period) and provide the citations. | 10-11 |
| Risk of bias within studies | 19 | Present data on risk of bias of each study and, if available, any outcome level assessment (see item 12). | 11-12 |
| Results of individual studies | 20 | For all outcomes considered (benefits or harms), present, for each study: (a) simple summary data for each intervention group (b) effect estimates and confidence intervals, ideally with a forest plot. | 11-14 |
| Synthesis of results | 21 | Present results of each meta-analysis done, including confidence intervals and measures of consistency. | 11-14 |
| Risk of bias across studies | 22 | Present results of any assessment of risk of bias across studies (see Item 15). | 11-12 |
| Additional analysis | 23 | Give results of additional analyses, if done (e.g., sensitivity or subgroup analyses, meta-regression [see Item 16]). | 11-14 |
| **DISCUSSION** | | |  |
| Summary of evidence | 24 | Summarize the main findings including the strength of evidence for each main outcome; consider their relevance to key groups (e.g., healthcare providers, users, and policy makers). | 14-17 |
| Limitations | 25 | Discuss limitations at study and outcome level (e.g., risk of bias), and at review-level (e.g., incomplete retrieval of identified research, reporting bias). | 17-18 |
| Conclusions | 26 | Provide a general interpretation of the results in the context of other evidence, and implications for future research. | 18-19 |
| **FUNDING** | | |  |
| Funding | 27 | Describe sources of funding for the systematic review and other support (e.g., supply of data); role of funders for the systematic review. | 2 |

*From:*  Moher D, Liberati A, Tetzlaff J, Altman DG, The PRISMA Group (2009). Preferred Reporting Items for Systematic Reviews and Meta-Analyses: The PRISMA Statement. PLoS Med 6(6): e1000097. doi:10.1371/journal.pmed1000097

For more information, visit: **www.prisma-statement.org**.

Page 2 of 2

**Table A in S1 File. Intervention Characteristics**

| **Study** | **General intervention**  **characteristics** | **Dietary intervention** | **Physical activity intervention** | **Behavioral intervention** | **Comparison group care** |
| --- | --- | --- | --- | --- | --- |
| Ackerman et al. 2008 | **Study design:** cluster RCT  **Duration:** 12m  **Frequency:** 1/yr for diet counseling; 1/w for PA  **No. of contacts:** NR  **Group/individual:** Individual;  **Medium:** In-person  **Facilitator:** NR  **Follow-up interval:** 12m | Caloric restriction; lifestyle intervention (weekly and then monthly) in the follow up periods the lifestyle intervention is weekly for 5w then monthly for 8m (eating, menu planning,) | Lifestyle interventions including places to walk and intensity (moderate for 150 min/w) | Personal advice about risk of developing DM to both groups; use of NDEP materials; YMCA wellness program information provided | Usual care (Brief advice for lifestyle change) |
| Almeida et al. 2011 | **Study design:** RCT  **Duration:** 12m  **Frequency:** 1/2m  **No. of contacts:** 6  **Group/individual:** Individuals;  **Medium:** In-person  **Facilitator:** Dietitian counselor  **Follow-up interval:** 12m | 6 individualized nutritional counseling program focusing on adequate energy and macronutrients intake, eating more fruit, vegetables, whole grain cereals, and fish | NR | NR | Usual care |
| Anderson et al. 2014 | **Study design:** RCT  **Duration:** 12m  **Frequency:** 1/m  **No. of contacts:** 15  **Group/individual:** Individuals;  **Medium:** In-person & phone  **Facilitator:** Trained lifestyle counsellor  **Follow-up interval**: 12m | 3 face-to-face lifestyle counsellor plus 15min/m call. A personalized energy prescription (2508 kj (600 kcal)below that required for weight maintenance) and bodyweight scales | PA used for one topic in visit | Goal setting, identifying intentions of implementation, self-monitoring of body weight, and counsellor feedback | Usual care |
| Anderssen et al. 1996 & 1998 Jacobs et al. 2009  The ODES Investigators 1993  Torjesen et al. 1997 | **Study design:** RCT  **Duration:** 12m  **Frequency:** 3/yr for diet counseling; 3/w for PA  **No. of contacts:** NR  **Group/individual:** Individual;  **Medium:** In-person  **Facilitator:** NR  **Follow-up interval:** 12m | Two individualized dietary counseling sessions focusing on reducing total caloric and saturated fat intake, increasing fish intake, and moderate salt restriction. | Aerobics, circuit training and fast walking/jogging, 3 /w, and intensity at 60-80% level of heart rate. | NR | Usual care |
| Arguin et al. 2012 | **Study design:** RCT  **Duration:** 12m  **Frequency:** 1/w for 17w  **No. of contacts:** 18  **Group/individual:** Both  **Medium:** In-person  **Facilitator:** Dietitian  **Follow-up interval:** 12m | 17 weekly group sessions on nutrition, health, and lifestyle habits focusing on weight loss and maintain, nutrition goal, 55%, 30% and 15% of energy intake from carbs, fats, and proteins | NR | NR | Both groups (continuous diet and intermittent diet) follow the same weight loss protocol for the first 5w. |
| Bazzano et al. 2014 | **Study design:** RCT  **Duration:** 12m  **Frequency:** 1/w for 4w, then 1/2w for 5m, then 1/m for 6m  **No. of contacts:** 20  **Group/individual:** Both  **Medium:** In-person  **Facilitator:** Dietitian  **Follow-up interval:** 12m | One-hour counselling session, low-carb group takes digestible carb of less than 40 g/d | NR | Followed a common behavioral curriculum | Low-fat group maintains less than 30% of their daily energy intake from total fat (<7% from saturated fat) and 55% from carb. |
| Bo et al. 2007&2009 | **Study design:** RCT  **Duration:** 12m  **Frequency:** 5/1yr  **No. of contacts**: 5  **Group/individual**: Individual  **Medium:** In-person  **Facilitator:** Physicians, nutritionists  **Follow-up interval:** 48m | Individually prescribed diet in first session. | Individualized with moderate intensity for 150 min PA /w. | 5 sessions of at least 60 min covering diet, exercise, and behavior modifications were held, the first was a one-to-one meeting and was followed by group sessions based on behavioral counseling and focusing on practical lifestyle tips | Usual care (Given general information on importance of a healthy lifestyle from their family physicians) |
| Bouchonville et al. 2014  Villareal et al. 2011 | **Study design:** RCT  **Duration:** 12m  **Frequency:** 1/w or 3/w  **No. of contacts**: NR  **Group/individual**: Both  **Medium:** In-person  **Facilitator:** Dietitian**,** physical therapist  **Follow-up interval**: 12m | Prescribed a balanced diet with a deficit of 500-750 kcal/d; goals for weight loss of 10% at 6mo, maintenance for the remaining 6mo. | Multicomponent exercise session with 90min duration (15-min flexibility, 30-min aerobic, 30-min progressive resistance, and 15-min balance exercise | NR | Usual care (Given general information) |
| Brinkworth et al; 2004 | **Study design:** RCT  **Duration:** 17m  **Frequency:** 1/2w for 16w  **No. of contacts:** 8  **Group/individual:** Individual  **Medium:** In-person  **Facilitator:** Dietitian  **Follow-up interval:** 17m | First 12w calorie-restricted diet (30% restriction or 6500 kJ/d)  30% protein; 40% carbs; 30% fat diet; Followed by a 4w period of energy balance with the same macronutrient composition. 52w follow-up. | NR | NR | High protein |
| Broekhuizen et al; 2012 | **Study design:** RCT  **Duration:** 12m  **Frequency:** 5/1yr  **No. of contacts:** 5  **Group/individual:** Individual  **Medium:** In-person + online  **Facilitator:** Dietitian  **Follow-up interval:** 12m | General Counseling on healthy diet | General counseling on adopting PA | Improve knowledge; motivation, self-efficacy for healthy behaviors and CVD risk reduction; non-smoking and statin therapy compliance | Usual care |
| Burke V et al; 2007 & 2008 | **Study design:** RCT  **Duration:** 4m  **Frequency:** 1/w  **No. of contacts:** 6  **Group/individual:** Both  **Medium:** In person  **Facilitator:** Dietitian  **Follow-up interval:** 36m | Low-sodium (<2 grams/day intake) diet | Recommended to accumulate 30 min of PA/d | Cognitive strategies used to maintain behavior change; Self-directed change, goal setting and time management. Social support encouraged through attendance of partner, relative or friend during sessions; involvement in grocery shopping, meal preparation and PA. | Usual care |
| Burtscher et al. 2009&2012 | **Study design:** RCT  **Duration:** 12m  **Frequency:** 1 /yr for diet counseling; 1/w for PA  **No. of contacts:** 52  **Group/individual:** Group  **Medium:** In-person  **Facilitator:** NR  **Follow-up interval:** 12m | Reduction in body weight of 5% or more, reduction in total fat intake to less than 30% of energy consumed, and reduction in saturated fat intake to less than 10% of energy | Advised to perform moderate exercise (walking, cycling, jogging, etc) for at least 30 min/d. supervised, progressive, individually tailored aerobic exercise programs and circuit type training for 1 hour twice a week. | NR | Usual care (Given written instructions on nutrition and physical activity) |
| Chirinos et al. 2016 | **Study design:** RCT  **Duration:** 12m  **Frequency:** 17 /yr  **No. of contacts:** 17  **Group/individual:** Group  **Medium:** In-person  **Facilitator:** Clinicians  **Follow-up interval:** 12m | Reducing weight by changing dietary habits. Calorie goals: 1200-1500 kcal/d for one weighting<100 kg; 1800-2000 kcal/d for one weighting ≥100k kg. | Unsupervised exercise: brisk walking start with 4 15-min/w in week 1, increase to 5 30-min/w in week 5. | Used modified DPP protocol to change diet and increase PA through education, behavior modification and stress management. | Usual care |
| Choo et al. 2014 | **Study design:** RCT  **Duration:** 12m  **Frequency:** 3/w  **No. of contacts:** 12  **Group/individual:** Both  **Medium:** In-person  **Facilitator:** Exercise trainers  **Follow-up interval:** 12m | Administered diet instruction with daily calorie intake (1200 kcal if weight<90.5kg; 1500 kcal if weight≥90.5kg) and fat intake goals (≤25% of total calories) | 60-min exercise session (3/w), 30-min treadmill, 30-min bike exercise with 50-70% of HRR. | 12 behavioral counselling sessions using behavior change strategies including goal setting and self-monitoring | Three PA groups (aerobic, resistance, and combination), no other comparison |
| Clifton et al. 2007 | **Study design**: RCT  **Duration**: 15m  **Frequency**: 1/m  **No. of contacts**: 4  **Group/individual**: Individual  **Medium**: In person  **Facilitator**: Dietician  **Follow-up interval**: 15m | Dieticians discussed use of high protein diet- 34% protein, 20% fat, 46% carbs | NR | NR | Low protein (high carbs) diet: low in saturated fat, 17% energy from protein, 20% fat, 64% from carbs |
| Cole et al. 2013 | **Study design**: RCT  **Duration**: 12m  **Frequency**: 3/yr  **No. of contacts**: 3  **Group/individual**: Both  **Medium**: In person  **Facilitator**: Dietician  **Follow-up interval:** 12m | A nutrition-based shared medical appointment intervention including lifestyle change | NR | NR | Usual care |
| Coon et al. 1989 | **Study design:** RCT  **Duration:** 9m  **Frequency:** 1/w for weight loss group,; 3/w for exercise group  **No. of contacts:** 36  **Group/individual:** Both  **Medium:** In-person  **Facilitator:** Dietitian; exercise physiologist  **Follow-up interval:** 12m | The weight loss program consisted of weekly sessions in which subjects were taught behavioral techniques to reduce caloric intake and principles of proper nutrition according to the American Heart Association | The endurance training consisted of supervised stationary bicycling  indoors and walking and jogging outdoors three times a week | The development of self-control to limit overeating and reduce caloric intake, understanding the influence of mood and emotions on eating behavior, and learning guidelines for the maintenance of weight loss after completion of the program. | Weight loss group vs exercise group |
| Cox et al. 2006&2008& 2010 | **Study design:** RCT  **Duration:** 12m  **Frequency:** 3/w for 6m  **No. of contacts:** 90  **Group/individual:** Group  **Medium:** In-person  **Facilitator:** Exercise physiologist  **Follow-up interval:** 12m | NR | 3/w PA for 24w. Each session included a 10-min warm-up and 5-min stretching, followed by 30 min of swimming or walking at a moderate intensity  (60%-70% heart rate reserve [HRR]), 10-min cooling  down, and 5-min stretching. | Half of the women received a behavioral intervention  package to encourage adoption and adherence to an exercise program; the other half had standard exercise information or “usual care.” | Swimming vs walking groups |
| Ditschuneit et al. 1999&2001 | **Study design:** RCT  **Duration:** 27m  **Frequency:** 1/m  **No. of contacts:** 27  **Group/individual:** Individual  **Medium:** In-person  **Facilitator:** Staff nutritionist  **Follow-up interval:** 27m | Monthly instruction of balanced diet: 1200-1500 kcal/d, 19-21% of energy as protein, 48-54% as carbs, 25-34% as fat | NR. | NR | Isoenergetic diet vs. energy-restricted diet |
| Donnelly et al, 2000 | **Study design:** RCT  **Duration:** 18m  **Frequency:** 3/w and 10/w  **No. of contacts:** NR  **Group/individual:** Individual  **Medium:** NR  **Facilitator:** Supervisor  **Follow-up interval:** 18m | Participants weren’t given a specific diet to follow but they did measure intake with 3d food records and 24hrs recall | Continuous group exercised for 30min at 60-75% of max aerobic capacity 3/w | NR | Intermittent group was instructed to walk briskly at 50-65% HRR 2/d 15 min/session 5 d/w at home or work with a min or 2hrs between sessions |
| Esposito et al. 2003 | **Study design:** RCT  **Duration:** 24m  **Frequency:** 1/m  **No. of contacts:** 18  **Group/individual:** Group  **Medium:** In-person  **Facilitator**: Nutritionist and exercise trainer  **Follow-up interval:** 24m | Advice on how to reduce 10% or more of body weight through Mediterranean diet, goal: 1300cal/d for the first year and 1500 cal/d for the second. 50-60% carbs, 15-20% protein and less than 30% fat. | Advice on PA, mainly walking, but also included swimming, or aerobic ball games | Education on how to reduce dietary calories, personal goal setting and self-monitoring through small group sessions. Behavioral and psychological counseling was offered. | Usual care |
| Esposito et al. 2004a | **Study design**: RCT  **Duration**: 24m  **Frequency**: 1/m for yr 1, ½m for yr 2  **No. of contacts**: 18  **Group/individual**: Group  **Medium**: In-person  **Facilitator**: Nutritionist, exercise trainer  **Follow-up interval**: 24m | Diet: monthly sessions with nutritionist year 1, then bimonthly year 2 | Monthly sessions with exercise trainer yr 1, then bimonthly yr 2 | Monthly sessions for reducing caloric intake, setting goals, and self monitoring; behavioral and psychological counseling offered | Usual care |
| Esposito et al. 2004b & 2009 | **Study design**: RCT  **Duration**: 24m  **Frequency**: 1/m for 1^st^ yr; ½m for 2^nd^ yr  **No. of contacts**: 18  **Group/individual**: Group  **Medium**: In-person  **Facilitator**: Nutritionist  **Follow-up interval**: 24m | Recommendations: carbs, 50%-60%; proteins, 15%-20%; total fat, <30%; saturated fat, <10%; cholesterol, <300 mg, fruits, 250-300 g; vegetables 125-150 g; walnuts 25-50 g; whole grains, 400 g; and increased consumption of olive oil. | Advised to increase their level of PA, mainly by walking ≥30 min /d, swimming or playing aerobic ball games (e.g., soccer). | Behavioral and psychological counseling | Usual care (Information on healthy food choices and the same general recommendations for macronutrient composition as intervention group) |
| Fatouros et al. 2005 | **Study design**: RCT  **Duration**: 12m  **Frequency**: 3/w for 24w  **No. of contacts**: 72  **Group/individual**: NR  **Medium**: In-person  **Facilitator**: NR  **Follow-up interval**: 12m. | NR | PA: Trained for 60 min with 8 resistance exercises, 2 sets/exercise for 8w then 3 sets w 9-24.  (low, medium and high intensity—LI, MI, HI)  LI: 45% max strength  MI: 60% max strength  HI: 80% max strength | NR | Usual care |
| Fernandez et al, 2012 | **Study design:** RCT  **Duration:** 12m  **Frequency:** Daily record food intake & discuss 2/m for 16w then every 3m  **No. of contacts:** NR  **Group/individual:** Individual  **Medium:** In-person  **Facilitator:** Dietician  **Follow-up interval:** 12m | 2 diet groups:  diet A group: 40%carbs/30%protein/30%fatdiet B group: 55%carbs/15%protein/30%fat | NR | NR | High carbs diet |
| Ferrara et al.2012 | **Study design:** RCT  **Duration:** 12m  **Frequency:** 1/m  **No. of contacts:** 6  **Group/individual:** Group  **Medium:** In-person  **Facilitator:** Dieticians, doctors  **Follow-up interval**: 12m | Mediterranean diet low in salt + 3 monthly sessions with information about hypertension, importance of diet for hypertension and other CV risk factors, how to avoid fatty/salty foods | Advice to do aerobic physical activity 3/w | Information on avoiding smoking | Usual care |
| Fischer et al. 2016 | **Study design:** RCT  **Duration:** 12m  **Frequency:** 6/w  **No. of contacts:** NR  **Group/individual:** Group  **Medium:** Online  **Facilitator:** Health coach, nutritionist, nurse  **Follow-up interval**: 12m | Modified National DPP curriculum using text messages related to nutrition. | Modified National DPP curriculum using text messages related to PA. | Modified National DPP curriculum using text messages related to motivation, skill teaching, problem solving, stress reduction.. | Usual care |
| Fisher et al, 2012 | **Study design:** RCT  **Duration:** 12m  **Frequency:** 2/w  **No. of contacts:** NR  **Group/individual:** NR  **Medium:** NR  **Facilitator: E**xercise physiology study personnel  **Follow-up interval:** 12m | Diet group: balanced diet focused on low-density food intake according to EatRight Weight Management program principles | Two exercise groups were scheduled to train 2/w- one group was aerobic and the other was resistance training. Both exercise groups were also assigned the same diet regimen as the diet-only group. | NR | Diet only |
| Fogelholm et al, 2000 | **Study design:** RCT  **Duration:** 12m  **Frequency:** Walk 2-3h/w for group 1; 4-6h/w for group 2  **No. of contacts:** 52  **Group/individual:** Group  **Medium:** In-person  **Facilitator:** Nutritionist  **Follow-up interval:** 33m | All subjects participated in weight-reduction for 12w with low-energy diet- then were randomized to 3 groups | Group 1: walking program targeted to expend 1004 kcal/w and group 2: walking program targeted to expend 2..8 kcal/w | Monitored high-risk situations for overeating and problems in diet and prevention of relapses were discussed at meetings | Usual care |
| Fonolla et al. 2009 | **Study design**: RCT  **Duration**: 12 m  **Frequency**: Daily  **No. of contacts**: Ongoing milk consumption at home  **Group/individual**: NR  **Medium**: NR  **Facilitator**: NR  **Follow-up interval**: 12m | Enriched milk had polyunsaturated fatty acid (PUFA), oleic acids, vitamins A, D, E, B6 and folic acid. | NR | NR | Provided enriched, semi-skimmed or skimmed milk for one year. |
| Frank et al, 2005 | **Study design:** RCT  **Duration:** 12m  **Frequency:** 5/w  **No. of contacts:**  NR  **Group/individual:** Both  **Medium:** In-person  **Facilitator:** NR  **Follow-up interval:** 12m | Moderate intensity exercise at least 45 min 5/w, 3 at supervised facility/w during months 1-3 and exercise 2d/w at home. For months 4-12 they attended 1/w at the facility and 4/w at home | NR | NR | Control group attended 1/w 45 min stretching class and asked not to exercise otherwise and maintain usual diet |
| Groeneveld et al. 2008 & 2010 | **Study design:** RCT  **Duration:** 12m  **Frequency:** 7/6m  **No. of contacts:** 7  **Group/individual:** Individual  **Medium:** In-person  **Facilitator:** Physicians and nurses as lifestyle coaches  **Follow-up interval:** 12m | Individualized client-centered counselling discussed and assisted in adopting individualized healthy eating via motivational interviews | Individualized client-centered counselling discussed and assisted in adopting individualized physical activity via motivational interviews | Motivational interviews [3 face-to-face (45-60 min each) and 4 phone contacts (15-30 min each)]: including introduction of health problem; choosing type of intervention; exploring ambivalence; determining readiness, willingness and ability; and goal setting. | Usual care |
| Heshka et al. 2003 | **Study design:** RCT  **Duration:** 24m  **Frequency:** 1/w  **No. of contacts:** 104  **Group/individual:** Groups;  **Medium:** In-person  **Facilitator:** Dietitian,  **Follow-up interval:** 24m | Intervention included a food plan – a nutritionally balanced, moderate-deficit diet designed for weight loss up to 0.9 kg/w. | An activity plan according to current NIH guidelines; Attendance at free Weight Watchers sessions supported by $9-vouchers given by the study | A behavior modification plan as part of programs for the intervention group, including weekly support group meetings | Usual care |
| Imayama et al. 2013 Foster-Schubert et al 2012 Mason et al. 2011&2013 | **Study design:** RCT  **Duration:** 12m  **Frequency:** 1/w in the first 6m, then 1/m in the next 6m  **No. of contacts:** 30  **Group/individual:** Both  **Medium:** In-person and e-mail/phone  **Facilitator:** Dietitian,  **Follow-up interval**: 12m | Based on the DPP, caloric intake of 1200-2000 kcal/d, ≤30% fat, 10% weight loss by week 24; with weight maintenance thereafter | 45min/d, 5d/w of moderate-to-vigorous intensity PA for 12m; 3 supervised sessions/w at facility, 2d/w at home till 70-80% of maximal HR | NR | Usual care |
| Juul et al. 2016 | **Study design:** RCT  **Duration:** 12m  **Frequency:** 4/5w in the first 5w  **No. of contacts:** 6  **Group/individual:** Group  **Medium:** In-person  **Facilitator:** Dietitian, occupational therapist  **Follow-up interval**: 12m | Weight reduction>5%, total-fat intake <30%, saturated-fat intake <10%, fibre-intake≥15g/1000kcal | Increase PA. | Lifestyle change based on transformative learning, health literacy theory, and dimensions of health knowledge and action competence. | Usual care |
| Kanaya et al. 2012 Delgadillo et al. 2010 | **Study design:** RCT  **Duration:** 12m  **Frequency:** NR  **No. of contacts:** 19  **Group/individual:** Both  **Medium:** In-person and phone  **Facilitator:** Counselor  **Follow-up interval**: 12m | Live Well, Be Well, 12 telephone counseling calls to educate healthy diet | Live Well, Be Well, 12 telephone counseling calls to educate PA | Self-selected and attainable goal-setting and action plans to enhance self-efficacy, and motivation | Usual care |
| Kanaya et al. 2014 | **Study design:** RCT  **Duration:** 12m  **Frequency:** 2/w 3m, 1/w 3m, 1/m 6  **No. of contacts:** 42  **Group/individual:** Both  **Medium:** In-person  **Facilitator:** Instructors  **Follow-up interval**: 12m | NR | Restorative yoga group practices lyengar poses in a group setting 2/w for 3m, 1/w for 3m, and 1/m for 6m, at home for 3 30min/w; | NR | Stretch group practices stretching as the same as yoga group |
| Katula et al. 2010 & 2011 & 2013 | **Study design**: RCT  **Duration**: 24m  **Frequency**: 4/m, plus 3 RD sessions in m 1-6; 2/m in m 7-24  **No. of contacts**: 65  **Group/individual**: Both  **Medium**: In person & telephone  **Facilitators**: CHWs and RDs  **Follow-up interval**: 24m | Weight loss goal: 1200-1800 kcal/d and to attain ~0.3kg weight loss/w for the first 6m and thereafter, unless BMI falls below 20 kg/m^2^. Dietary topics included nutrition basics, energy balance, healthy eating. | Fitness improvements achieved through moderate PA (≥180 min/w) | Self-efficacy, outcome expectations and incentives targeted, group dynamics and problem-solving with 5 steps: 1) problem-orientation; 2) problem definition and formulation; 3) generation of alternatives; 4) systematic decision-making, and 5) implementation and verification. | Usual care (Individual education program, build awareness of existing community resources and enhance retention. Included 2 individual sessions with a nutritionist during the first 3 months and a quarterly newsletter on healthy lifestyle) |
| Kawano, et al. 2009 | **Study design:** RCT  **Duration:** 12m  **Frequency:** 1/m for diet counseling; 1/w for PA  **No. of contacts:** 109  **Group/individual:** Individual;  **Medium:** In-person  **Facilitator:** Dietitian  **Follow-up interval:** 17m | Monthly dietary classes for 5m with a dietitian | Ball exercises, exercise ergometers, aquatic exercise, tube exercises and stretching (2/w for at least 30 min in addition to their regular activities) | NR | Usual care (Attended lifestyle disease seminars and given written health information) |
| Keogh et al.2007. | **Study design**: RCT  **Duration**: 12m  **Frequency**: ½w  **No. of contacts**: NR  **Group/individual**: Individual  **Medium**: In-person  **Facilitator**: Registered dietitian  **Follow-up interval**: 12m | Low carbs- 33% carbs, 27% fat, 40% protein 7% sat fat, 6% PUFA, 13% monounsaturated fatty acids (MUFA), 26 g fiber | NR | NR | High carbs – 60% carbs, 20% fat, 20% protein 4% sat fat, 5% PUFA, 7% MUFA, 40 g fiber |
| Lawton et al. 2009 | **Study design:** RCT  **Duration:** 12m  **Frequency:** 1/w  **No. of contacts:** 6  **Group/individual:** Individual  **Medium:** In-person  **Facilitator:** Exercise facilitator  **Follow-up interval:** 24m | NR | Motivational interview to increase PA; aerobic exercise 150 min/w (brisk walking for 30 min/5d/w); 7-13 min initial counseling with a Green Exercise prescription; phone support for 3m (15-min- five calls); a 30-min visit at M 6. | Counseled to increase PA using motivational interview techniques | Usual care |
| Lim et al. 2010 | **Study design:** RCT  **Duration:** 3m  **Frequency:** Visits at m 3, 6, 9, 12, and 15  **No. of contacts:** 11  **Group/individual:** Individual  **Medium:** In-person  **Facilitator:** Dietitian  **Follow-up interval:** 12m | 3 isocaloric diets: (1) very low carbs (60% fat, 4% carbs) (2) very low fat (10% fat, 70% carbs); high unsaturated fat (30% fat, 50% carb); Offered a prescriptive meal plan with food for 65% energy consumption; | NR | NR | Usual care |
| Lombard et al. 2010 | **Study design: Cluster** RCT  **Duration:** 4m  **Frequency:** Group sessions at w 1, 2, 3, & 16  **No. of contacts:** 15  **Group/individual:** Group;  **Medium:** In-person  **Facilitator:** Dietitian  **Follow-up interval:** 12m | Not used energy restriction or expenditure goals. | No specific assignment; encouraged to join voluntary school-based walking groups or to walk with friends; a pedometer provided. | Goal setting, self-monitoring, social support, problem solving, & relapse prevention; also focused on behavior change skills related to diet and PA; required to attend 4 1-hour group sessions (W 1, 2, 3, and 16); follow-up support with text messages | Usual care |
| Ma et al. 2009 & 2013 | **Study design**: RCT  **Duration**: 3m  **Frequency**: 1/w in m 1-3  **No. of contacts**: 12  **Group/individual**: Group  **Medium**: In-person or DVD, plus email  **Facilitators**: RD & lifestyle coach  **Follow-up interval**: 15m | 12-session, recommendations: 25% of calories from fat, daily intake reduction of 500-1000 calories, <10% saturated fat, <300mg/d cholesterol, more fruits and vegetables, whole grains and low-fat dairy, and reduced intake of high glycemic index carbohydrates. | Goals were 7% weight loss and 150 min of moderate PA/w, increasing to 60 min/d if weight loss goal not achieved. Gradually and steadily increase their PA; coach led group also had 30-45 min. guided PA after each weekly class. | Enhancing behavior capability through goal-setting, skill building and self monitoring and building self efficacy, positive outcome expectations, and self management skills, including problem-solving, action planning, decision-making and support | Usual care |
| Marrero et al. 2016 | **Study design**: RCT  **Duration**: 12m  **Frequency**: 1/w  **No. of contacts**: NR  **Group/individual**: Group  **Medium**: In-person  **Facilitators**: Trained group leaders  **Follow-up interval**: 12m | Modified DPP model with modest weight loss (5-10%) via caloric restriction, dietary modification | Modeled DPP with modest weight loss (5-10%) via moderate PA. | Modeled DPP with modest weight loss (5-10%) via self-monitoring of weight, stimulus control, and relapse prevention. | Usual care |
| Marsh et al. 2010 | **Study design:** RCT  **Duration:** 12m  **Frequency:** 1/w  **No. of contacts:** NR  **Group/individual:** Individual  **Medium:** In-person/phone/email  **Facilitator:** Dietician  **Follow-up interval:** 12m | Reduced energy, low-fat, low saturated fat, moderate-to –high fiber, low glycemic index carbs. Menu plans, shopping lists, key carbs foods provided every 4-6 w, newsletter sent out to increase compliance. | Both groups informed of the benefits of PA and encouraged to exercise. | NR | Same diet plan, but with moderate-to-high glycemic index (GI) foods instead of low GI foods. |
| Mason et al. 2016 | **Study design:** RCT  **Duration:** 5.5m  **Frequency:** 1/w for 12w, ½w for 3w, ¼w for the last one  **No. of contacts:** 16  **Group/individual:** Group  **Medium:** In-person  **Facilitator:** Dietician  **Follow-up interval:** 12m | Diet-exercise program with mindfulness training, focusing on modest caloric reduction by 500 calories. | Diet-exercise program with mindfulness training, focusing on increasing PA: bicycling, swimming, strength training, and walking | Diet-exercise program with mindfulness training, focusing on mind training, stress reduction. | Diet-exercise program without mindfulness training |
| McAuley et al. 2005&2006 | **Study design:** RCT  **Duration:** 4m  **Frequency:** 1/w  **No. of contacts:** 17  **Group/individual:** NR  **Medium:** In-person  **Facilitator:** NR  **Follow-up interval:** 12m | High-fat group**:** >20g of carbs daily for 1^st^ 2w, increased by 5g/d for w 3-8 up to 50g/d by w8. W 8-16 5g/d increase  High-protein group**:** 40% should be from low GI carbs, 30% from protein and 30% from fat | All groups advised to participate in 30 min of any activity 5 d/w | NR | High-carbs, high-fiber diet |
| Mellberg et al. 2014 | **Study design:** RCT  **Duration:** 24m  **Frequency:** 8/6m, then 4/18m  **No. of contacts:** 12  **Group/individual:** Both  **Medium:** In-person  **Facilitator:** Dietitian  **Follow-up interval:** 24m | Palaeolithic-type diet with 30% of energy intake (E%) from protein, 40 E% fat and 30 E% carb, and high intake of MUFA and PUFA | NR | NR | Nordic Nutrition Recommendations group with 15 E% protein, 25-30 E% fat, and 55-60 E% carb. |
| Muto et al. 2001 | **Study design**: RCT  **Duration**: 18m  **Frequency**: 2/12m  **No. of contacts**: 3  **Group/individual**: Group  **Medium**: In-person  **Facilitator**: Physician, dietician, exercise trainer  **Follow-up interval**: 18m | 4d group program with education on nutrition, through lectures, training, individual counseling, and group discussion. | 4d group program with education on PA through lectures, training, individual counseling, and group discussion | 4d group program with education on stress, CVD risk factors through lectures, training, individual counseling, and group discussion | Usual care (did not attend program but still had yearly health exam and If had lab abnormalities, were advised by mail or their physician) |
| Narayan et al. 1998 | **Study design:** RCT  **Duration:** 12m  **Frequency:** 1/w  **No. of contacts:** NR  **Group/individual:** Group  **Medium:** In-person  **Facilitator:** NR  **Follow-up interval:** 12m | Reduce fat and alcohol intake; increase fiber intake and receive advice from dietician | Walking, water aerobics, softball, volleyball, community farming and gardening, cleaning local cemetery | Weekly meetings reinforced home visits with behavioral techniques, modeling/role play | Self directed learning, culture, history and basic printed materials |
| Nilsson et al. 1992 | **Study design:** RCT  **Duration:** 12m  **Frequency:** 1/m  **No. of contacts:** NR  **Group/individual:** Group  **Medium:** In-person  **Facilitator:** Nurse, dietician or physiotherapist  **Follow-up interval:** 12m | Diet fat<30% of daily energy intake, a polyunsaturated: saturated fat ratio of (0.8-1.0) and more mono-and polyunsaturated fats per meal. Daily cholesterol intake <200mg, daily dietary fiber intake 30g, | 4 special physical activity sessions/y (2hrs outdoor walking) and special physical activity sessions every 2w for the eight most sedentary men. Participation in other physical activities and sports was recommended. | 1m period with seminars, videotapes, and counseling on lifestyle, then put into groups that met monthly for 1-2h with a nurse. Guided tours of supermarkets, cooking, and distribution of free olive oil to help participants adhere to the healthier lifestyle | Usual care |
| Nilsson et al. 2001 | **Study design:** RCT  **Duration:** 12m  **Frequency:** 16/12m  **No. of contacts:** 16  **Group/individual:** Both  **Medium:** In-person  **Facilitator:** Nurses, nutritionists, physiotherapists, a physician  **Follow-up interval:** 18m | NR | NR | Individual counseling by a nurse and group sessions covering weight reduction, improved diet and PA, stress management and smoking cessation. Program included lectures, discussions, videos, and outdoor activities. | Usual care |
| Ockene, et al. 2012  Merriam et al. 2009 | **Study design**: RCT  **Duration**: 12m  **Frequency**: 1/1yr for diet counseling; 1/w for PA  **No. of contacts**: 16  **Group/individual**: Both  **Medium**: In-person  **Facilitator**: NR  **Follow-up interval**: 12m | Increase whole grain intake, decrease intake of nonstarchy vegetables, sodium, total and saturated fat, portions of carbs and refined carbs | Increase walking by 4000 steps/d over baseline | Social Cognitive Theory based to promote positive attitudes towards behavioral change and increase self efficacy | Usual care |
| Poston et al. 2006 | **Study design:** RCT  **Duration:** 12m  **Frequency:** 1/m  **No. of contacts:** 13  **Group/individual:** Individual  **Medium:** In-person  **Facilitator:** Nurse  **Follow-up interval:** 12m | Counseling groups received individual dietary recommendations, including 30% calories consumed daily from fat and encouraged to keep a food diary | Counseling groups encouraged to expend 200 cal/d in exercise and given suggestions on how to accomplish this, encouraged to keep an exercise diary | Monthly 15-20 min sessions, encouraged to make one behavioral change/m, focused on cognitive-behavioral methods such as self-monitoring, stimulus control, cognitive restructuring and stress management skills. | Usual care |
| Potteiger et al. 2002&2003 | **Study design:** RCT  **Duration:** 6m  **Frequency:** 3-5 d/w  **No. of contacts:** NR  **Group/individual:** Group  **Medium:** In-person  **Facilitator:** NR  **Follow-up interval:** 16m | Ad libitum diet that was 30–35% fat, 45–55% carbs, and 10 – 25% protein | 20min/session at baseline to 45min/session 6m and beyond, intensity from 60% of HRR at baseline to 75% at 6m and beyond, 55-70% Vo2max. Minimum energy expenditure | NR | Usual care |
| Reid et al. 2014 | **Study design:** RCT  **Duration:** 12m  **Frequency:** 1/w 12w,  **No. of contacts:** 17  **Group/individual:** Both  **Medium:** In-person and phone  **Facilitator:** Educator  **Follow-up interval:** 12m | Counseling including heathy eating | Counseling session including PA | Counseling session including face-to-face and phone, regarding goal-setting, barriers overcome | Usual care |
| Rossner et al. 1997 | **Study design:** RCT  **Duration:** 12m  **Frequency:** BL, 6w, 26w, 52w, final assessment  **No. of contacts:** 4  **Group/individual:** Group  **Medium:** In-person  **Facilitator:** Research nurse & a dietician  **Follow-up interval:** 12m | Nutrilett group was given a supplement to take with water over 5 meals with 1 vitamin & 1 fish-oil tab (420 kcal/d); VLCD (530 kcal/d) and LCD (880 kcal/d) had no food until week 7 and then began a “balanced hypocaloric diet” | No formal PA was organized or monitored, but walking was encouraged | VLCD and LCD groups had continuous behavioral monitoring reinforcement of balanced diet instructions | Nutrilett group with allowance of 5 meals/d |
| Ryttig et al. 1997 | **Study design:** RCT  **Duration:** 26m.  **Frequency:** 1/w for 1^st^ m, ½w for 2^nd^ m  **No. of contacts:** 25  **Group/individual:** Group  **Medium:** In-person  **Facilitator:** Research nurse & a dietician  **Follow-up interval:** 26m | Group A pts. Ate a balanced diet of 1,600 kcal/d during the entire treatment period. Other pts. In a combined VLCD (Group B + C) ate a diet of 420 kcal/d of Nutrilett nutrition powder with a balanced diet. | No formal PA prescribed, instructed to maintain the same PA habits as they typically do during entire trial. | No formal behavioral treatment prescribed, instructed to maintain the same smoking habits as they typically do during entire trial. | 1600 kcal/d diet |
| Sartorelli et al. 2005 | **Study design:** RCT  **Duration:** 12m  **Frequency:** ½m  **No. of contacts:** 6  **Group/individual:** Group  **Medium:** In-person  **Facilitator:** Research assistants & nutritionist  **Follow-up interval:** 12m | Received diet prescription by a nutritionist with a food exchange list | 30 min of walking/d encouraged | NR | Usual care |
| Sattin et al. 2016 | **Study design:** RCT  **Duration:** 3m  **Frequency:** 1/w for 12w  **No. of contacts:** 12  **Group/individual:** Group  **Medium:** In-person  **Facilitator:** Health provider, church health advisors  **Follow-up interval:** 12m | Fit Body and Soul (FBAS) program used modified DPP model to weight loss>7% | Increase PA by at least 150 MET-min/w. | Behavioral modification: stimulus control, goal setting, and problem solving. | Health Education (HE) program |
| Simkin-Silverman et al. 1995&1998&2003  Kuller et al. 2001&2006&2012 | **Study design:** RCT  **Duration:** 4m  **Frequency:** 15/20w, then 3/m followed by 2-3/12m  **No. of contacts:** 30  **Group/individual:** Group  **Medium:** In-person/phone/email  **Facilitator:** Trained nutritional & behavioral interventionists  **Follow-up interval:** 54m | Lowering intake of total fat to 25% of daily calories, saturated fat to 7% and total cholesterol. To 100 mg/d; followed a 1,300 – 1,500 cal meal plan for 4w | Gradually increased PA to 1,000 kcal/w; emphasized walking | Taught behavioral strategies: problem-solving, stimulus control, goal setting, assertiveness training, relapse prevention, & cognitive techniques to maintain diet & exercise | Usual care |
| Siu et al. 2015 | **Study design:** RCT  **Duration:** 12m  **Frequency:** 3/w for 12m  **No. of contacts:** 156  **Group/individual:** Group  **Medium:** In-person  **Facilitator:** yoga instructors  **Follow-up interval:** 12m | NR | Three 60-min yoga sessions weekly for 1 year. | NR | Usual care |
| Staten et al. 2004 | **Study design:** RCT  **Duration:** 12m  **Frequency:** 1/1yr  **No. of contacts:** 1  **Group/individual:** individual  **Medium:** In-person  **Facilitator:** NR  **Follow-up interval:** 12 months | Increase fruit and vegetable consumption incrementally with an overall goal of 5+ servings/d | Accumulation of 150+ min/w of moderate to vigorous PA | Monthly newsletter, reminder calls to participate, social support by community health workers, use of social cognitive theory | Provider counseling only |
| Stefanick et al. 1998 | **Study design:** RCT  **Duration:** 12m  **Frequency:** 3/w  **No. of contacts:** Diet & Diet + exercise:16; exercise only:38  **Group/individual:** Group  **Medium:** In-person/phone/mail  **Facilitator:** Registered dieticians and exercise staff  **Follow-up interval:** 12m | Diet & Diet + exercise groups both followed the National Cholesterol Education Program Step 2 diet; 12w adoption phase with mixed-sex group sessions | Exercise group & Diet+ exercise group had an initial meeting followed by a 6w, phase of 3 sessions/w (1 hr. ea.; treadmill); 6 to 8m. maintenance phase of brisk walking & mixed-sex group sessions | NR | Usual care |
| Tapsell et al. 2014 | **Study design:** RCT  **Duration:** 12m  **Frequency:** 6/yr  **No. of contacts:** 6  **Group/individual:** Individual  **Medium:** In-person  **Facilitator:** Dietitian  **Follow-up interval:** 12m | All requested to consume at least 5 servings of vegetables each day focusing on vegetables, fruit, grain foods, meat/fish/eggs/cheese, milk/yoghurt, and nuts/seeds/spreads/oils, but different in 0.5 vs 1.0 cup cooked; 1 vs 2.0 cups of raw, respectively. | NR | NR | Two diet groups |
| ter Bogt et al. 2009 | **Study design:** RCT  **Duration:** 12m  **Frequency:** BL, and at 1, 2, 3, 5, 8, and 12m  **No. of contacts:** 7 **Group/individual:** Group  **Medium:** In-person  **Facilitator:** Nurse practitioner  **Follow-up interval:** 12m | NR | NR | Nurse practitioner group provided computer-guided lifestyle counseling | Usual care |
| Thompson et al. 2005 | **Study design:** RCT  **Duration:** 12m  **Frequency:** 1/2w  **No. of contacts:** NR  **Group/individual:** Individual  **Medium:** In-person  **Facilitator:** Dietitian  **Follow-up interval:** 12m | Three diet interventions with a cal deficit of 500 cal/d comparing a moderate calcium diet (800 mg) to a high calcium (1400 mg) diet and high calcium + high fiber diet. Food diaries completed and reviewed weekly | Patients instructed to exercise (e.g., brisk walking, treadmill, or exercise bicycle) at least 30 min four times a week. | NR | Moderate calcium diet (standard) |
| Tsai et al. 2010 | **Study design:** RCT  **Duration:** 18m  **Frequency:** 0, 2, 4, 8, 12, 16,  20, and 24 weeks  **No. of contacts:** 8  **Group/individual:** Individual  **Medium:** In-person  **Facilitator:** Medical assistant  **Follow-up interval:** 18m | Patients were instructed to consume 1,200–1,500 kcal/d (if <250 lb) or 1,500–1,800 kcal/d (if ≥250 lb), to keep daily records of their food intake, Patients were weighed at each visit and then reviewed their food and  activity record | Patients were instructed to gradually increase their PA to 175 min/w (e.g., by walking). | Patients in both groups were provided 1–2 page handouts developed by the Weight-Control Information  Network of the National Institutes of Health, received a calorie counter, a pedometer, and a sample meal plan | Usual care |
| Vainionpaa et al. 2007 | **Study design:** RCT  **Duration** 12m  **Frequency:** 3/w for 12m  **No. of contacts:** 36  **Group/individual**: Both  **Medium:** In-person  **Facilitator:** Physiotherapist  **Follow-up interval:** 12m | NR | Each workout lasted 60 min, including a 10-min warm-up, a 40-min high-impact training, and a 10-min cool-down and stretching period. Additionally, the participants were asked to train for 10 min daily at home | NR | Usual care |
| Vetter et al. 2013 Wadden et al. 2011 | **Study design:** RCT  **Duration** 24m  **Frequency:** 1/m counseling session, 1/3m primary care provider visit  **No. of contacts:** 32  **Group/individual**: Both  **Medium:** In-person  **Facilitator:** Primary care providers  **Follow-up interval:** 24m | A balance diet: 1200-1500 kcal/d for pts <113.6kg; 1500-1800 kcal/d for pts≥113.6kg. 15-20% from protein, 20-35% from fat | Increasing PA to 180min/w, provided pedometer. | DPP-like lesson | Usual care |
| Von Thiele Schwarz et al. 2008 | **Study design:** RCT  **Duration:** 12m  **Frequency:** 24 w  **No. of contacts:** 3  **Group/individual:** Individual  **Medium:** In-person  **Facilitator:** NR  **Follow-up interval:** 12m | NR | 2.5 hrs of weekly workhours were allocated to mandatory PA on 2 days. PA was medium to high intensity at 55-89% maximum heart rate (vigorous). Participants were allowed to choose any activity. | None | Usual care |
| Wantanabe et al. 2003 | **Study design:** RCT  **Duration:** 12m  **Frequency:** 1/12m for diet counseling  **No. of contacts:** 2  **Group/individual:** Both  **Medium:** In-person  **Facilitator:** NR  **Follow-up interval:** 12m | Control portions; assess intake, glucose control and individual dietary counseling, learning to read menus | NR | Increase motivation to improve dietary practice | General oral and written information about results of the health examination and results of the questionnaire but without detailed explanation  Convention diet |
| Weinstock et al. 1998 | **Study design:** RCT  **Duration:** 24m  **Frequency:** 1/w for 1^st^ 28w  **No. of contacts:** NR  **Group/individual:** Group  **Medium:** In-person  **Facilitator:** Psychologist/ dietitian  **Follow-up interval:** 24m | Started with 4 servings of liquid diet (925kcal/d) with a dinner entrée and a salad. Each serving of the liquid provided 150 kcal. After 18w start increasing consumption of conventional foods and diet went up to 1250kcal/d | The exercise group included 3 on-site supervised training sessions (first 28w), 2 workouts/w (w28-49) and unsupervised thereafter. Subjects were randomized to either aerobic or strength training exercises. | NR | Diet only |
| Weiss et al. 2006 | **Study design:** RCT  **Duration:** 12m  **Frequency:** 1/3m  **No. of contacts:** 5  **Group/individual:** Both  **Medium:** In-person  **Facilitator:** Dietitian, exercise technician  **Follow-up interval:** 12m | Decrease calorie intake by 16% during the first 3m and by 20% during the remaining 9m, encouraging reductions in portion size and to substitute foods with a low calorie density with those with a high calorie density. | Increasing exercise energy expenditure by 16% of baseline total daily energy expenditure for the first 3m and by 20% for the subsequent 9m | NR | Usual care |
| Wing et al. 1995 | **Study design:** RCT  **Duration:** 18m  **Frequency:** 0, 6, 12, 18m  **No. of contacts:** 38  **Group/individual:** Individual  **Medium:** In-person  **Facilitator:** NR  **Follow-up interval**: 18m | Emphasizing on complex carbs and reduced fats. 1000 or 1500 cal/d diets based on the initial weight. The calorie intake was gradually increased if they achieved weight-loss goal 14, 18 or 24 kg. | Exercise in the form of walking or cycling gradually increasing from 250kcal/w to 1000 kcal/w | NR | Usual care |
| Wing et al. 1998 | **Study design:** RCT  **Duration:** 24m  **Frequency:** 1/1yr for diet counseling;  **No. of contacts:** 38  **Group/individual:** Group  **Medium:** In-person  **Facilitator:** NR  **Follow-up interval:** 24m | Low calorie and low fat regimen; 800-1000kcal/d diet, with 20% calories as fat (weeks 1-8), then adjust 1200-1500kcal/d diet; self monitored calorie intake | Gradually increase PA to 1500 kcal/w through brisk walking 5d/w (3 miles/d) and monitor daily exercise; introducing line dancing, step aerobics and community walking events to participants | Presented with information to help change their eating behavior, recipe modification, stimulus control, assertion, behavior chain analysis, problem solving and relapse prevention | Usual care |
| Wycherley et al. 2012 | **Study design:** RCT  **Duration:** 12m  **Frequency:** 3/1yr  **No. of contacts:** 3  **Group/individual:** Group  **Medium:** In-person  **Facilitator:** NR  **Follow-up interval:** 12m | Isocaloric and moderate energy restricted diet, high protein (HP): 35% protein; 40% carbs; 25% fat. | NR | NR | High carbs (HC): 17% protein; 58% carbs; 25% fat. |
| Yeh et al. 2016 | **Study design:** RCT  **Duration:** 12m  **Frequency:** 1/2w for 6m, 1/m for 6m  **No. of contacts:** 18  **Group/individual:** Group  **Medium:** In-person  **Facilitator:** Physicians, community leaders  **Follow-up interval:** 12m | Modified DPP model focusing on healthy eating, rice bowls for portion control. | Modified DPP model focusing on PA. | Modified DPP model focusing on stress reduction and problem-solving skill. | Usual care |

The primary study is listed first, followed by additional citations for related publications.

Abbreviations: cal: calorie; carbs: carbohydrates; CV: cardiovascular; CVD: cardiovascular disease; d: day; ea: each; GI: glycemic index; hr: hour; HR: heart rate; HRR: heart rate reserve; Kcal: kilocalorie; kg: kilogram; LCD: low calorie diet; m: month; min: minute; MUFA: monounsaturated fatty acid; NR: not reported; PA: physical activity; PUFA: polyunsaturated fatty acid; RCT: randomized control trail; VLCD: very low calorie diet; VO2max: maximal oxygen uptake; vs: versus; w: week; yr: year.

**Table B in S1 File. Quality Assessment**

| **Study** | **Setting** | **Participants Characteristics** | **Intervention Characteristics (general)** | **Intervention Characteristics (specific)** | **Outcomes** | **Quality Assessment** | |
| --- | --- | --- | --- | --- | --- | --- | --- |
| **Citation** | **Follow-up (months)**  **No. study arms**  **Setting**  **Number** | **Age (y)**  **Sex** (%F)  **Health condition**:  **BL wt, BMI:**  **BL FPG**:  **BL A1C:** | **Duration (m)**  **Frequency**  **No. contacts**  **Group/individual**  **Medium**  **Facilitator**  **Comparison group care** | **Diet:**  **PA:**  **Behavioral:** | **BMI:**  **FPG:**  **A1C:**  **Insulin:**  **Cholesterol:**  **LDL:**  **HDL:**  **TG:**  **SBP:**  **DBP:**  **Other:** | **Sampling method**  **Jadad Score**  **Randomization procedure**  **Allocation** **concealment Attrition (%)**  **Blinding pt**  **Blinding assessor**  **Blinding provider**  **BL comparable** | |
| Ackerman, et al. 2008 | **Follow-up**: 12m  **No. study arms:** 2  **Setting**: Community, Indianapolis, community  **Number**: 92 | **Age (y):** 58.3  **Sex (%F)**: 25.5  **Health condition**: At risk for DM  **BL wt:** 92.7 kg  **BMI:** 31.3 kg/m^2^  **BL FPG**: NR  **BL A1C:** 5.6%  **BL Insulin:** NR | **Duration:** 12m  **Frequency:** 1/yr for diet counseling; 1/w for PA  **No. contacts:** NR  **Group/individual**: Individual  **Medium**: In-person  **Facilitator**: Unclear  **Comparison group care**: UC | **Diet**: Caloric restriction; LI (1/w and then 1/m) in the follow up periods the LI is 1/w for 5w then 1/m for 8m (eating, menu planning,)  **PA:** LI including places to walk and intensity (moderate for 150 min/w)  **Behavioral:** Personal advice about risk for DM; use of NDEP materials; YMCA wellness program information provided | **BMI:** y  **FPG:** n  **A1C:** y  **Insulin:** n  **Cholesterol:** y  **LDL:** n  **HDL:** y  **TG**: n  **SBP:** y  **DBP**: y  **Other**: NR | **Sampling method:** Population screen  **Jadad Score:** 1,0,0, C  **Randomization procedure:** Using a sealed envelop  **Allocation concealment:** Unclear  **Attrition (%):** 32.6  **Blinding pt:** NR  **Blinding assessor:** No  **Blinding provider:** No  **BL comparable:** ND | |
| Almeida et al; 2011 | **Follow-up**: 12m  **No. study arms:** 2  **Setting**: Sao Paulo, Brazil; HIV reference clinic. **Number**: 53 | **Age (y):** NR; 50% were 30-39  **Sex (%F)**: 18.9  **Health condition**: HIV on HAART  **BL wt, BMI:** 23.3 kg/m^2^  **BL FPG**: 4.7 mmol/L  **BL A1C:** NR  **BL Insulin:** NR | **Duration:** 12m  **Frequency** **:** 1 Nutrition counseling session every 2m  **No. contacts:** 6  **Group/individual**: Individual  **Medium**: In-person  **Facilitator**: NR  **Comparison group care:** UC | **Diet**1 Nutrition counseling session every 2m. Client-centered approach; Counseling based on Brazilian dietary guidelines.  **PA:** NR  **Behavioral:** NR | **BMI:** y  **FPG:** y  **A1C:** n  **Insulin:** n  **Cholesterol:** y  **LDL:** y  **HDL:** y  **TG:** y  **SBP:** y  **DBP**: y  **Other:** WC; skinfolds; | **Sampling method:** NR  **Jadad Score:** 0,0,0,C  **Randomization procedure:** NR  **Allocation** **concealment:** Unclear  **Attrition (%):** 20.8  **Blinding pt:** NR  **Blinding assessor:** No  **Blinding provider:** No  **BL comparable:** ND | |
| Anderson et al. 2014 | **Follow-up**: 12m  **No. study arms:** 2  **Setting**: Scotland, UK **Number**: 329 | **Age (y):** 63.6  **Sex (%F)**: 26  **Health condition**: Polypectomy for adenoma  **BL wt, BMI:** 30.7 kg/m^2^  **BL FPG**: 6.1 mmol/L  **BL A1C:** 6.0%  **BL Insulin:** 10.6 µU/ml | **Duration:** 12m  **Frequency:** 1/m  **No. contacts:** 15  **Group/individual**: Individual  **Medium**: In-person, call  **Facilitator**: Trained lifestyle counsellor  **Comparison group care**: UC | **Diet:** A personalized energy prescription (2508 kj (600 kcal)below that required for weight maintenance) and bodyweight scales  **PA**: PA used for one topic in visit  **Behavioral**: Goal setting, identifying intentions of implementation, self-monitoring of body weight, and counsellor feedback | **BMI:** y  **FPG:** y  **A1C:** y  **Insulin:** y  **Cholesterol:** y  **LDL:** y  **HDL:** y  **TG:** y  **SBP:** y  **DBP:** y  **Other**: PA and dietary intake | **Sampling method:** Population screen  **Jadad Score:** 2,0,1, C  **Randomization procedure:** Using a permuted block technique  **Allocation** **concealment:** Adequate  **Attrition (%):** 7.3  **Blinding pt:** No  **Blinding assessor:** Yes  **Blinding provider:** No  **BL comparable:** ND | |
| Anderssen et al. 1996 & 1998 Jacobs et al. 2009  The ODES Investigators 1993  Torjesen et al. 1997 | **Follow-up**: 12m  **No. study arms:** 4  **Setting**: Norway, Oslo, community  **Number**: 219 | **Age (y):** 44.9  **Sex (%F)**: 9.6  **Health condition**: Atherothrombogenic syndrome  **BL wt, BMI:** 28.8 kg/m^2^  **BL FPG**: 5.6 mmol/L  **BL A1C:** NR  **BL Insulin:** NR | **Duration:** 12m  **Frequency:** 3/yr for diet counseling; 3/w for PA  **No. contacts:** NR  **Group/individual**: Individual  **Medium**: In-person  **Facilitator**: Unclear  **Comparison group care:** UC | **Diet:** 3 individualized dietary counseling sessions focusing on reducing total caloric and saturated fat intake, increasing fish intake, and moderate salt restriction.  **PA:** Aerobics, circuit training and fast walking/jogging, 3/w, and intensity at 60-80% level of heart rate.  **Behavioral:** NR | **BMI:** y  **FPG:** y  **A1C:** n  **Insulin:** y  **Cholesterol:** y  **LDL:** n  **HDL:** y  **TG:** y  **SBP:** y  **DBP:** y  **Other:** NR | **Sampling method:** Population screen  **Jadad Score:** 1,0,0, C  **Randomization procedure:** Using a sealed envelop  **Allocation** **concealment:** Unclear  **Attrition (%):** 4.6  **Blinding pt:** NR  **Blinding assessor:** No  **Blinding provider:** No  **BL comparable:** FBS higher in CG, but controlled for in analysis | |
| Arguin et al. 2012 | **Follow-up**: 12m  **No. study arms:** 2  **Setting**: Research Center; Quebec, Canada  **Number**: 25 | **Age (y):** 60.5  **Sex (%F)**: 100  **Health condition**: Pre-menopausal/overweight  **BL wt, BMI:** NR  **BL FPG**: 5.0 mmol/L  **BL A1C:** NR  **BL Insulin:** NR | **Duration:** 12m  **Frequency** **:**  17 weekly nutrition sessions  **No. contacts:**17  **Group/individual**: Both  **Medium:** In-person  **Facilitator**: Dietitian  **Comparison group care:**  Continuous vs intermittent diet arms. No true control group. | **Diet**: Dietitian supervision on macronutrient diet components (55% carbs. 30% fat, 15% protein). Daily weighing at home. Dietitian available for consultation if not losing 1% of weight/w. 17 weekly group lessons on nutrition and healthy lifestyles.  **PA:** NR  **Behavioral:** NR | **BMI:** n  **FPG:** y  **A1C:** n  **Insulin:** n  **Cholesterol:** y  **LDL:** y  **HDL:** y  **TG:** y  **SBP:** n  **DBP:** n  **Other:** WC; %fat and lean body mass; RMR; | **Sampling method:** Population screen  **Jadad Score:** 2,0,1,C  **Randomization procedure:** Computer-generated randomization list  **Allocation** **concealment:** Unclear  **Attrition (%):** 12.0  **Blinding pt:** NR  **Blinding assessor:** NR  **Blinding provider:** No  **BL comparable:** Dietary fiber intake higher in C. | |
| Bazzano et al. 2014 | **Follow-up**: 12m  **No. study arms**: 2  **Setting**: Tulane University, new Orleans, LA  **Number** : 148 | **Age (y):** 46.8  **Sex (%F)**: 88.5  **Health condition**: Obese people  **BL wt, BMI:** 35.4 kg/m^2^  **BL FPG**: 5.2 mmol/L  **BL A1C:** NR  **BL Insulin:** 17.3 µU/ml | **Duration:** 12m  **Frequency** **:**  1/w for 4w, then 1/2w for 5m, then 1/m for 6m  **No. contacts:**20  **Group/individual**: Both  **Medium:** In-person  **Facilitator**: Dietitian  **Comparison group care**: Low fat vs low carb diet arms. No true control group | **Diet**: Low-carb group takes digestible carb of less than 40 g/d; Low-fat group maintains less than 30% of their daily energy intake from total fat (<7% from saturated fat) and 55% from carb.  **PA:** NR  **Behavioral:** Followed a common behavioral curriculum NR | **BMI:** y  **FPG:** y  **A1C:** n  **Insulin:** y  **Cholesterol:** y  **LDL:** y  **HDL:** y  **TG:** y  **SBP:** y  **DBP:** y  **Other:** WC; body weight; | **Sampling method:** Population screen  **Jadad Score:** 2,0,1,C  **Randomization procedure:** Computer-generated randomization  **Allocation** **concealment:** Unclear  **Attrition (%):** 17.8  **Blinding pt:** NR  **Blinding assessor:** NR  **Blinding provider:** No  **BL comparable:** ND | |
| Bo et al. 2007&2009 | **Follow-up**: 48m  **No. study arms**: 2  **Setting**: Northwest Italy  **Number** : 375 | **Age (y)** : 55.7  **Sex (%F)**: 58.2  **Health condition**: MetS  **BL wt, BMI**: 29.7 kg/m^2^  **BL FPG**: 5.8 mmol/L  **BL A1C**: NR  **BL Insulin:** NR | **Duration**: 48 m  **Frequency**: 5/y  **No. contacts**: 5  **Group/individual**: Both  **Medium**: In-person  **Facilitator:** physicians, nutritionists  **Comparison group care**: UC | **Diet**: Individually prescribed in first session  **PA**: individualized with moderate intensity for 150 min/w  **Behavioral:** 5 sessions of 60-min covering behavior modifications, a one-to-one meeting, followed by group sessions based on behavioral counseling and focusing on practical lifestyle tips | **BMI**: y  **FPG**: y  **A1C**: n  **Insulin**: y  **Cholesterol**: n  **LDL**: n  **HDL**: y  **TG**: y  **SBP:** y  **DBP**: y  **Other**: HOMA-B, HOMA-IR, waist, weight | **Sampling method**: Clinic screening  **Jadad Score**; 1, 0, 1, C  **Randomization procedure**: Random sequence generation  **Allocation concealment**: Adequate  **Attrition (%**): 10.7  **Blinding pt**: No  **Blinding assessor**: No  **Blinding provider**: No  **BL comparable**: ND | |
| Bouchonville et al. 2014  Villareal et al 2011 | **Follow-up**: 12m  **No. study arms**: 4  **Setting**: Washington University, St. Louis, MO  **Number** : 107 | **Age (y)** : 69.7  **Sex (%F)**: 62.6  **Health condition**: older and obese persons  **BL wt, BMI**: 37.2 kg/m^2^  **BL FPG**: 5.5 mmol/L  **BL A1C**: NR  **BL Insulin:** 16.6 µU/ml | **Duration**: 12m  **Frequency**: 1/w or 3/w  **No. contacts**: NR  **Group/individual**: Both  **Medium**: In-person  **Facilitator:** Dietitian**,** physical therapist  **Comparison group care:** UC | **Diet**: Prescribed a balanced diet with a deficit of 500-750 kcal/d;  **PA**: 90min duration (15-min flexibility, 30-min aerobic, 30-min progressive resistance, and 15-min balance exercise **Behavioral:** NR | **BMI**: y  **FPG**: y  **A1C**: n  **Insulin**: y  **Cholesterol**: n  **LDL**: n  **HDL**: y  **TG**: y  **SBP:** y  **DBP**: y  **Other:** HOMA-IR, waist, weight | **Sampling method**: Public advertisement  **Jadad Score**; 1, 0, 1, C  **Randomization procedure**: Generated algorithm  **Allocation concealment**: Adequate  **Attrition (%**): 13.0  **Blinding pt**: No  **Blinding assessor**: NR  **Blinding provider**: NR  **BL comparable**: ND | |
| Brinkworth et al; 2004 | **Follow-up**: 12m  **No. study arms:** 2  **Setting** Outpatient Clinic; Adelaide, Australia  **Number**: 58 | **Age (y):** 50.2  **Sex (%F)**: 77.6  **Health condition**: Insulin >12uU/ml; BMI between 27 and 43;  **BL wt, BMI:** 34.0 kg/m^2^  **BL FPG**: 5.4 mmol/L  **BL A1C:** NR  **BL Insulin:** 16.0 µU/ml | **Duration:** 12m  **Frequency** **: :** Every 2w for 16w  **No. contacts**: 18  **Group/individual**: Individual  **Medium:** In-person  **Facilitator**: Dietitian  **Comparison group care:** UC | **Diet**: First 12w calorie-restricted diet (30% restriction or 6500 kJ/d)  30% protein; 40% carbs; 30% fat diet; Followed by a 4w period of energy balance with the same macronutrient composition. 52w follow-up.  **PA:** NR  **Behavioral:** NR | **BMI:** y  **FPG:** y  **A1C:** n  **Insulin:** y  **Cholesterol:** y  **LDL:** y  **HDL:** y  **TG:** y  **SBP:** y  **DBP:** y  **Other:** % body fat; lean body mass, dietary intake, | **Sampling method:** Public advertisement  **Jadad Score:** 0,0,1,C  **Randomization procedure:** NR  **Allocation** **concealment:** unclear  **Attrition (%):** 25.9  **Blinding pt:** No  **Blinding assessor:** No  **Blinding provider:** No  **BL comparable:** ND | |
| Broekhuizen et al; 2012 | **Follow-up**: 12m  **No. study arms:** 2  **Setting** Outpatient Clinic; Amsterdam, Netherlands  **Number**: 340 | **Age (y):** 45.3  **Sex (%F)**: 56.7  **Health condition**: Hypercholesterolemia; LDL > 75th percentile;  **BL wt, BMI:** 26.5 kg/m^2^  **BL FPG**: 4.9 mmol/L  **BL A1C:** NR  **BL Insulin:** NR | **Duration:** 12m  **Frequency:** 5 counseling sessions/12m  **No. contacts:** 5  **Group/individual**: Individual  **Medium:** In-person  **Facilitator**: Dietitian  **Comparison group care:** UC | **Diet**: General Counseling on healthy diet  **PA:** General counseling on adopting PA  **Behavioral:** Improve knowledge; motivation, self-efficacy for healthy behaviors and CVD risk reduction; non-smoking and statin therapy compliance | **BMI:** y  **FPG:** y  **A1C:** n  **Insulin:** n  **Cholesterol:** y  **LDL:** y  **HDL:** y  **TG:** y  **SBP:** y  **DBP**: y  **Other:** NR | **Sampling method:**  From cascade screening program  **Jadad Score:** 2,1,1,A  **Randomization procedure:** Random number list computerized  **Allocation** **concealment:** Adequate  **Attrition (%):** 7.4  **Blinding pt:** Yes  **Blinding assessor:** Yes  **Blinding provider:** No  **BL comparable:** ND | |
| Burke V et al; 2007 & 2008 | **Follow-up**: 36m  **No. study arms:** 2  **Setting** Clinic; Perth, Australia  **Number**: 241 | **Age (y):** 56.2  **Sex z(%F)**: 55.6  **Health condition**: Overweight and obese adults  **BL wt, BMI:** 30.1 kg/m^2^  **BL FPG**: 5.0 mmol/L  **BL A1C:** NR  **BL Insulin:** 1.8 µU/ml | **Duration:** 36m  **Frequency:** **:** weekly first 6m; every 2w for next 3m and monthly for the last 3m  **No. contacts:** 32  **Group/individual**: Group  **Medium:** In-person  **Facilitator**: Clinic educator + dietitian  **Comparison group care:** UC | **Diet**: Calorie restricted diets (1200 to 1500 cal for women; 1500 to 1800 for men); 25% from fat;  **PA**: 150mins of PA/d by the initial 6w and thereafter increase or maintain that goal. Walking recommended as PA**.**  **Behavioral:** Self-monitoring; goal setting; cognitive re-structuring; stimulus control; demonstrations and skill development. | **BMI:** y  **FPG:** y  **A1C:** n  **Insulin:** y  **Cholesterol:** y  **LDL:** y  **HDL:** y  **TG:** y  **SBP:** n  **DBP:** n  **Other:** PA, food intake, WC | **Sampling method:**  Recruitment at clinic  **Jadad Score:** 0,0,1,C  **Randomization procedure:** NR  **Allocation** **concealment:** Unclear  **Attrition (%):** 16.2  **Blinding pt:** NR  **Blinding assessor:** NR  **Blinding provider:** No  **BL comparable:** ND | |
| Burtscher et al. 2009&2012 | **Follow-up:** 12m  **No. study arms:** 2  **Setting:** Clinic, Western Austria  **Number:** 36 | **Age (y):** 57.5  **Sex (%F):** 56.0  **Health condition**: IFG: 100-125 mg/dL  **BL wt:** 83.2 kg  **BMI**: 29.0 kg/m^2^  **BL FPG**: 6.o mmol/L  **BL A1C:** NR  **BL Insulin:** NR | **Duration:** 12m  **Frequency:** 1/w  **No. contacts:** NR  **Group/individual:** Group  **Medium:** In-person  **Facilitator:** Exercise scientist  **Comparison group care**: Counseling alone | **Diet:** Fat intake < 30% of energy, saturated fat intake to <10% of energy  **PA:** PA (walking, cycling, jogging, etc) for at least 30 min/d. Given a list of training facilities near them,  individually tailored aerobic exercise programs and circuit type training for 2 1-hour/w  **Behavioral:** NR | **BMI:** y  **FPG:** y  **A1C:** n  **Insulin:** n  **Cholesterol:** n  **LDL:** n  **HDL:** y  **TG:** n  **SBP:** y  **DBP:** y  **Other:** NR | **Sampling method:** Clinic screening  **Jadad Score :**1,0,0, C  **Randomization procedure:** Nested cohort approach  **Allocation** **concealment:** NR  **Attrition(%):** 0  **Blinding pt:** No  **Blinding assessor:** No  **Blinding provider:** No  **BL comparable:** ND | |
| Chirinos et al. 2016 | **Follow-up:** 12m  **No. study arms:** 2  **Setting:** Clinic, FL  **Number:** 120 | **Age (y):** 51.7  **Sex (%F):** 55.8  **Health condition**: MetS  **BL wt:** 87.9 kg  **BMI**: NR  **BL FPG**: 4.9 mmol/L  **BL A1C:** NR  **BL Insulin:** 1.5 µU/ml | **Duration:** 12m  **Frequency:** 17 /yr  **No. of contacts:** 17  **Group/individual:** Group  **Medium:** In-person  **Facilitator:** Clinicians  **Comparison group care**: UC | **Diet:** 1200-1500 kcal/d for one weighting<100 kg; 1800-2000 kcal/d for one weighting ≥100k kg.  **PA:** brisk walking start with 4 15-min/w in week 1, increase to 5 30-min/w in week 5.  **Behavioral:** behavior modification and stress management. | **BMI:** n  **FPG:** y  **A1C:** n  **Insulin:** y  **Cholesterol:** n  **LDL:** n  **HDL:** y  **TG:** y  **SBP:** y  **DBP:** y  **Other**: insulin resistance | **Sampling method:** Community screening  **Jadad Score :**2,1,1,A  **Randomization procedure:** A block randomization schedule  **Allocation** **concealment:** Adequate  **Attrition(%):** 22.5  **Blinding pt:** No  **Blinding assessor:** Yes  **Blinding provider:** Yes  **BL comparable:** ND | |
| Choo et al. 2014 | **Follow-up:** 12m  **No. study arms:** 3  **Setting:** Community, Seoul South Korea  **Number:** 110 | **Age (y):** 43.1  **Sex (%F):** 100  **Health condition**: Elevated WC  **BL wt:** 72.4 kg  **BMI**: 28.5 kg/m^2^  **BL FPG**: 5.0 mmol/L  **BL A1C:** NR  **BL Insulin:** NR | **Duration:** 12m  **Frequency:** 3/w  **No. contacts:** 20  **Group/individual:** Both  **Medium:** In-person  **Facilitator:** Exercise trainer  **Comparison group care:** Three PA groups | **Diet:** Daily calorie intake (1200 kcal if weight<90.5kg; 1500 kcal if weight≥90.5kg) and fat (≤25% of total cal)  **PA:** 60-min exercise session (3/w), 30-min treadmill, 30-min bike exercise with 50-70% of HRR.  **Behavioral:** Behavior change strategies including goal setting and self-monitoring | **BMI:** y  **FPG:** y  **A1C:** n  **Insulin:** n  **Cholesterol:** y  **LDL:** y  **HDL:** y  **TG:** y  **SBP:** y  **DBP:** n  **Other**: VO2max | **Sampling method:** Community screening  **Jadad Score :**2,0,1,C  **Randomization procedure:** A random allocation computer program  **Allocation** **concealment:** NR  **Attrition(%):** 55.0  **Blinding pt:** No  **Blinding assessor:** Yes  **Blinding provider:** No  **BL comparable:** ND | |
| Clifton et al. 2007. | **Follow-up**: 12m  **No. study arms** : 2  **Setting**: Australia, community  **Number**: 119 | **Age (y)**: 49.0  **Sex (%F)**: 100  **Health condition**: health women with BMI 27-40  **BL wt, BMI**: 32.8 kg/m^2^  **BL FPG**: 6.1 mmol/L  **BL A1C**: NR  **BL insulin**: 9.9 µU/ml | **Duration:** 12m  **Frequency**: every 4w; then follow up every 3m afterwards  **No. contacts**: 4  **Group/individual**: Individual  **Medium**: In-person  **Facilitator**: Dietician  **Comparison group care**: High carbohydrate diet | **Diet**: High protein diet- 34% protein, 20% fat, 46% carbs  **PA**: NR  **Behavioral**: NR | **BMI**: n  **FPG**: y  **A1C**: n  **Insulin**: y  **Cholesterol**: n  **LDL**: y  **HDL**: y  **TG**: n  **SBP**: n  **DBP**: n  **Other**: Triacylglyerol, CRP | **Sampling method**: Public advertisement  **Jadad Score**: 1, 0, 1, C  **Randomization procedure** : NR  **Allocation concealment**: Unclear  **Attrition (%)**: 33.6  **Blinding pt**: No  **Blinding assessor**: No  **Blinding provider**: No  **BL comparable**: ND | |
| Cole et al. 2013 | **Follow-up**: 12m  **No. study arms** : 2  **Setting**: San Antonio, Texas, community  **Number**: 94 | **Age (y)**: 58.3  **Sex (%F)**: 46  **Health condition**: Pre-DM with IFG of 100-125 mg/dL  **BL wt, BMI**: 30.8 kg/m^2^  **BL FPG**: 6.1 mmol/L  **BL A1C**: 5.9%  **BL insulin**: NR | **Duration:** 12m  **Frequency**: 1/m in the first 3m  **No. contacts**: 3  **Group/individual**: Both  **Medium**: In-person  **Facilitator**: Dietician  **Comparison group care:** UC | **Diet**: A nutrition-based shared medical appointment intervention including lifestyle change  **PA**: NR  **Behavioral**: NR | **BMI**: y  **FPG**: y  **A1C**: y  **Insulin**: n  **Cholesterol**: y  **LDL**: y  **HDL**: y  **TG**: y  **SBP**: y  **DBP**: y  **Other:** PA | **Sampling method**: Health care system enroller  **Jadad Score**: 2, 0, 0, C  **Randomization procedure** : Computer-generated random-number list  **Allocation concealment**: Unclear  **Attrition (%)**: 31.0  **Blinding pt**: No  **Blinding assessor**: No  **Blinding provider**: No  **BL comparable**: ND | |
| Coon et al; 1985 | **Follow-up**: 12m  **No. study arms:** 2  **Setting** Outpatient Clinic; Baltimore, MD, USA  **Number**: 20 | **Age (y):** 59.5  **Sex (%F)**: 0  **Health condition**: Healthy older  **BL wt, BMI:** 29.0 kg/m^2^  **BL FPG**: 5.4 mmol/L  **BL A1C:** NR  **BL Insulin:** 13.0 µU/ml | **Duration:** 12m  **Frequency** **:** Weekly counseling sessions  **No. contacts:** 30  **Group/individual**: Group  **Medium:** In-person  **Facilitator**: Dietitian + exercise physiologist  **Comparison group care:** weight loss vs exercise group | **Diet**: Reduce caloric intake  and follow the AHA principles of proper nutrition  **PA**: The endurance training consisted of supervised stationary bicycling  indoors and walking and jogging outdoors 3/w  **Behavioral**: Development of patterns of self-control to limit  overeating and reduce caloric intake, understanding the influence of mood and emotions on eating behavior | **BMI:** y  **FPG:** y  **A1C:** n  **Insulin:** y  **Cholesterol:** y  **LDL:** y  **HDL:** y  **TG:** y  **SBP:** y  **DBP:** y  **Other:** Fat free mass, WHR, VO2max; fat mass | **Sampling method:**  By advertisement  **Jadad Score:** 0,0,1,C  **Randomization procedure:** NR  **Allocation** **concealment:** unclear  **Attrition (%):** 0  **Blinding pt:** NR  **Blinding assessor:** NR  **Blinding provider:** No  **BL comparable:** Weight loss group had higher weight and fat free mass | |
| Cox et al; 2010 | **Follow-up**: 12m  **No. study arms:** 2  **Setting:** Community setting; Perth, Australia  **Number**: 116 | **Age (y):** 55.5  **Sex (%F)**: 100  **Health condition**: Healthy sedentary women  **BL wt, BMI:** 26.4 kg/m^2^  **BL FPG**: 5.1 mmol/L  **BL A1C:** NR  **BL Insulin:** 6.2 µU/ml | **Duration:** 12 m  **Frequency** **:** 3/w supervised PA sessions  **No. contacts:** 90  **Group/individual**: Group  **Medium:** In-person  **Facilitator**: Exercise physiologist  **Comparison group care:** Walking vs swimming program | **Diet**: NR  **PA**: Supervised 3/w PA for 6m (24w). Each session included a 10-min warm-up and 5-min stretching, followed by 30-min of swimming or walking at a moderate intensity (60%-70% HRR),  **Behavioral**: Encourage adoption and adherence to an exercise program | **BMI:** y  **FPG:** y  **A1C:** n  **Insulin:** y  **Cholesterol:** y  **LDL:** y  **HDL:** y  **TG:** y  **SBP:** y  **DBP:** y  **Other:** WHR, VO2max; energy intake | **Sampling method:**  Community screen  **Jadad Score:** 2,0,1,C  **Randomization procedure:** Random number list computerized  **Allocation** **concealment:** Unclear  **Attrition (%):** 25.9  **Blinding pt:** NR  **Blinding assessor:** NR  **Blinding provider:** No  **BL comparable:** ND | |
| Ditschuneit et al, 2001 | **Follow-up:** 24m  **No. study arms**: 2  **Setting:** Clinics, Ulm Germany  **Number:** 100 | **Age (y):** 45.7  **Sex** (%F): 79.0  **Health condition**: Overweight or obesity  **BL wt, &BMI:** 33.4 kg/m^2^  **BL FPG**: 5.0 mmol/L  **BL A1C:** NR  **BL Insulin:** 21.5 µU/ml | **Duration:** 24m  **Frequency:** 1/m  **No. contacts:** 27  **Group/individual:** Individual  **Medium:** In-person  **Facilitator:** Staff nutritionist  **Comparison group care:** Isoenergetic diet vs. energy-restricted diet | **Diet:** Monthly instruction of balanced diet: 1200-1500 kcal/d, 19-21% of energy as protein, 48-54% as carbs, 25-34% as fat  **PA:** NR  **Behavioral:** NR | **BMI:** n  **FPG:** y  **A1C:** n  **Insulin:** y  **Cholesterol:** y  **LDL:** n  **HDL:** y  **TG:** y  **SBP:** y  **DBP:** y  **Other**: NR | **Sampling method:** Hospital referral  **Jadad Score:** 2, 0, 1, C  **Randomization procedure:** Using computer-generated number  **Allocation** **concealment:** unclear  **Attrition (%):** 27.0  **Blinding pt:** No  **Blinding assessor:** No  **Blinding provider:** No  **BL comparable:** ND | |
| Donnelly et al, 2000 | **Follow-up:** 18m  **No. study arms:** 2  **Setting:** University lab  **Number :** 22 | **Age (y):** 51.5  **Sex** (%F): 100  **Health condition**: BMI >25, low aerobic capacity and represented a pop at risk for continued weight gain, not currently engaged in PA  **BL wt:** 83.6kg  **BMI:** 31.2 kg/m^2^  **BL FPG**: 5.5 mmol/L  **BL A1C:** NR  **BL Insulin:** 14.0 µU/ml | **Duration:** 18m  **Frequency:** Group one 3/w; group two 10/w  **No. contacts:** NR  **Group/individual:** Individual  **Medium:** In-person  **Facilitator:** supervisor in performance lab  **Comparison group care**: Intermittent group | **Diet:** NR  **PA:** Continuous group exercised for 30mins at 60-75% of max aerobic capacity 3/w; Intermittent group was instructed to walk briskly at 50-65% HRR 2/d 15 mins per session 5 d/w at home or work with a min or 2hrs between sessions  **Behavioral:** NR | **BMI**: y  **FPG:** y  **A1C:** n  **Insulin:** y  **Cholesterol:** y  **LDL:** n  **HDL:** y  **TG:** n  **SBP:** y  **DBP:** y  **Other**: energy intake, waist, hip, VO2, HR | **Sampling method:** NR  **Jadad Score:** 0, 0, 0, C  **Randomization procedure:** NR  **Allocation** **concealment:** Unclear  **Attrition (%):** 0  **Blinding pt:** NR  **Blinding assessor:** NR  **Blinding provider:** NR  **BL comparable:** ND | |
| Esposito et al. 2003 | **Follow-up:** 24m  **No. study arms:** 2  **Setting:** Clinic  **Number:** 120 | **Age (y):** 34.6  **Sex** (%F): 100%  **Health condition:** Pre-menopausal obese women  **BL wt, BMI:** 34.9 kg/m^2^  **BL FPG**: 5.9 mmol/L  **BL A1C:** NR  **BL Insulin:** 14.0 µU/ml | **Duration:** 24 m  **Frequency:** 1/m  **No. of contacts:** NR  **Group/individual:** Group  **Medium:** In-person  **Facilitator:** Nutritionist and exercise trainer  **Comparison group care:** UC | **Diet:** 1300 cal/d for the first year and 1500 cal/d for the second. 50-60% carbs, 15-20% protein and less 30% fat.  **PA:** Women received advice on PA, mainly walking, but also included swimming, or aerobic ball games  **Behavioral:** Education on how to reduce dietary calories, personal goal setting and self-monitoring. | **BMI:** y  **FPG:** y  **A1C:** n  **Insulin**: y  **Cholesterol:** y  **LDL:** n  **HDL:** y  **TG:** y  **SBP:** y  **DBP:** y  **Other:** waist / hip circumference | **Sampling method**: Outpatient department pts  **Jadad Score**: 2,1,1, A  **Randomization procedure:** Using envelopes  **Allocation** **concealment:** Adequate  **Attrition (%):** 6.7  **Blinding pt:** No  **Blinding assessor:** Yes  **Blinding provider:** Yes  **BL comparable:** ND | |
| Esposito et al. 2004a | **Follow-up**: 24m  **No. study arms**: 2  **Setting**: Naples, Italy; clinical  **Number** : 110 | **Age (y)** : 43.3  **Sex (%F)**: 0  **Health condition**: Obese men with erectile dysfunction  **BL wt, BMI**: 36.7 kg/m^2^  **BL FPG**: 5.8 mmol/L  **BL A1C**: NR  **BL insulin**: 20.0 µU/ml | **Duration:** 24 m  **Frequency:** 1/m  **No. contacts**: 24  **Group/individual**: Group  **Medium:** In- person  **Facilitator**: Nutritionist, exercise trainer  **Comparison group care**: UC (General oral and written information at baseline and bimonthly visits) | **Diet**: Monthly sessions with nutritionist yr 1, then bimonthly yr 2  **PA**: Monthly sessions with exercise trainer yr 1, then bimonthly yr 2  **Behavioral**: Monthly sessions for setting goals, and self-monitoring; behavioral and psychological counseling offered | **BMI**: y  **FPG**: y  **A1C**: n  **Insulin**: y  **Cholesterol**: y  **LDL**: n  **HDL**: y  **TG**: y  **SBP**: y  **DBP**: y  **Other:** weight | **Sampling method**: Recruited from clinics  **Jadad Score**: 2,0,1, C  **Randomization procedure** : Computer generated  **Allocation concealment**: Adequate  **Attrition (%):** 5.5  **Blinding pt**: No  **Blinding assessor**: Yes  **Blinding provider**: No  **BL comparable**: ND | |
| Esposito et al. 2004b & 2009 | **Follow-up**: 24 m  **No. study arms**: 2  **Setting**: Naples, Italy, University hospital  **Number**: 180 | **Age (y)**: 43.9  **Sex (%F):** 45.0  **Health condition**: Metabolic syndrome  **BL wt:** 77.5 kg  **BMI**: 28.0 kg/ m^2^  **BL FPG**: 6.3 mmol/L  **BL A1C**: NR  **BL insulin**: 15.5 µU/ml | **Duration**: 24 m  **Frequency**: 12/y 1st y; 6/y 2nd y  **No. contacts**: 18  **Group/individual**: Group  **Medium**: In-person  **Facilitator**: Nutritionist  **Comparison**: Usual care (Information on healthy food choices) | **Diet**: Carb, 50%-60%; proteins, 15%-20%; total fat, <30%; saturated fat, <10%; cholesterol, <300 mg, fruits, 250-300 g; vegetables 125-150 g; walnuts 25-50 g; whole grains 400 g; and increased consumption of olive oil.  **PA**: increase their level of PA, walking ≥30 min/d, swimming or playing aerobic ball games (eg, soccer).  **Behavioral**: Behavioral and psychological counseling | **BMI**: y  **FPG**: y  **A1C**: n  **Insulin**: y  **Cholesterol**: y  **LDL**: n  **HDL**: y  **TG**: y  **SBP**: y  **DBP**: y  **Other**: WC, HOMA score, endothelial function score, hs-CRP and cytokines, | **Sampling method**: Patient screen  **Jadad Score:**  2,0,0,C  **Randomization procedure**: Computer-generated number **Allocation** **concealment**: Adequate  **Attrition (%)**: 8.9  **Blinding pt**: No  **Blinding assessor**: Yes  **Blinding provider**: No  **BL comparable**: ND | |
| Fatouros et al. 2005 | **Follow-up**: 12m  **No. study arms**: 4  **Setting**: Community, Greece  **Number**: 50 | **Age (y):** 70.4  **Sex (%F)**: 0  **Health condition**: Inactive men  **BL wt, BMI**: 29.5 kg/m^2^  **BL FPG**: 5.9 mmol/L  **BL A1C**: NR  **BL insulin**: 14.2 µU/ml | **Duration:** 12 m  **Frequency**: 3/w x 24w  **No. contacts**: 72  **Group/individual**: Unclear  **Medium**: In-person  **Facilitator**: Unclear  **Comparison group care**: UC | **Diet**: NR  **PA**: 60mins with 8 resistance exercises with 2 sets/exercise x 8w then 3 sets w 9-24.  (low, medium and high intensity-- LI, MI, HI)  LI: 45% max strength  MI: 60% max strength  HI: 80% max strength  **Behavioral**: NR | **BMI**: y  **FPG**: y  **A1C**: n  **Insulin**: y  **Cholesterol**: n  **LDL**: n  **HDL**: n  **TG**: n  **SBP**: n  **DBP**: n  **Other**: weight | **Sampling method**: Recruited from nursing homes  **Jadad Score** : 0, 0, 1, C  **Randomization procedure**: NR  **Allocation concealment**: Unclear  **Attrition (%)**: 0  **Blinding pt**: No  **Blinding assessor**: No  **Blinding provider**: No  **BL comparable**: ND | |
| Fernandez et al, 2012 | **Follow-up:** 12m  **No. study arms:** 4  **Setting:** Spain- a university  **Number:** 40 | **Age (y):** 40.9  **Sex** (%F): 67.5  **Health condition**: Overweight/obese  **BL wt, :** 88.0 kg  **BMI:**  31.8 kg/m^2^  **BL FPG**: 4.6mmol/L  **BL A1C:** NR  **BL insulin**: 21.2 µU/ml | **Duration:** 12m  **Frequency:** daily record food intake & discuss 2/m for 16w then every 3m  **No. contacts: U**nclear  **Group/individual:** Individual  **Medium:** In-person  **Facilitator:** Dietician  **Comparison group care:** Another diet group as comparison | **Diet:** 2 diet groups for IR and IS groups: diet A: 40%carbs/30%protein/30%fat; diet B: 55%carbs/15%protein/30%fat  **PA:** NR  **Behavioral:** NR | **BMI:** y  **FPG:** y  **A1C: n**  **Insulin:** y  **Cholesterol:** y  **LDL:** y  **HDL:** y  **TG:** y  **SBP:** n  **DBP:** n  **Other:** HOMA-IR, macronutrients, total kcal | **Sampling method:** Recruited from community **Jadad Score:** 1, 0, 0, C  **Randomization procedure :** Using a website  **Allocation** **concealment:** Unclear  **Attrition (%):** 60  **Blinding pt:** No  **Blinding assessor:** Yes  **Blinding provider:** No  **BL comparable:** ND | |
| Ferrara et al. 2012 | **Follow-up**: 24m  **No. study arms**: 2  **Setting**: Clinic + small groups  **Number**: 188 | **Age (y):** 56.4  **Sex (%F):** 47.9  **Health condition**: HT people with stable BP  **BL wt, BMI**: 29.2 kg/m^2^  **BL FPG**: 5.6 mmol/L  **BL A1C**: NR  **BL insulin**: NR | **Duration:** 24m  **Frequency**: every 1m x 3, and every 3m x 3  **No. contacts**: 6  **Group/individual**: Group  **Medium**: In-person  **Facilitator**: Dietician and doctors  **Comparison group care**: UC (Advice for Mediterranean diet) | **Diet**: Mediterranean diet low in salt + information about HT and importance of diet for HT and other CV risk factors, how to avoid fatty/salty foods  **PA**: Advice to do aerobic physical activity 3 /w  **Behavioral:** Information on avoiding smoking | **BMI**: y  **FPG**: y  **A1C**: n  **Insulin**: n  **Cholesterol**: y  **LDL**: y  **HDL**: y  **TG**: y  **SBP**: y  **DBP**: y  **Other**: weight | **Sampling method**: Recruited from clinic  **Jadad Score** : 0,0,1, C  **Randomization procedure**: NR  **Allocation concealment**: Unclear  **Attrition (%):** 0  **Blinding pt**: No  **Blinding assessor**: Yes  **Blinding provider**: No  **BL comparable**: ND | |
| Fischer et al. 2016 | **Follow-up**: 12m  **No. study arms**: 2  **Setting**: Clinic  **Number**: 163 | **Age (y):** 46.4  **Sex (%F):** 75.8  **Health condition**: PreDM  **BL wt,** 89.2 kg  **BMI**: NR  **BL FPG**: NR  **BL A1C**: 6.0%  **BL insulin**: NR | **Duration:** 12m  **Frequency:** 6/w  **No. of contacts:** NR  **Group/individual:** Group  **Medium:** Online  **Facilitator:** Health coach, nutritionist, nurse  **Comparison group care**: UC | **Diet**: Modified National DPP curriculum using text messages related to nutrition.  **PA**: Increase PA  **Behavioral:** Motivation, skill teaching, problem solving, stress reduction | **BMI**: n  **FPG**: n  **A1C**: y  **Insulin**: n  **Cholesterol**: n  **LDL**: n  **HDL**: n  **TG**: n  **SBP**: y  **DBP**: n  **Other**: weight | **Sampling method**: Recruited from clinic  **Jadad Score** : 2,0,1, B  **Randomization procedure**: A clock-generated seed  **Allocation concealment**: Unclear  **Attrition (%):** 5.7  **Blinding pt**: NR  **Blinding assessor**: NR  **Blinding provider**: NR  **BL comparable**: ND | |
| Fisher et al, 2012 | **Follow-up:** 12m  **No. study arms:** 3  **Setting:** NR  **Number:** 97 | **Age (y):** Range: 21-46  **Sex** (%F): 100  **Health condition**: Premenopausal, sedentary  **BL wt,:** 77.3kg  **BMI:** 28.0 kg/m^2^  **BL FPG**: 4.8 mmol/L  **BL A1C:** NR  **BL insulin**: 11.4 µU/ml | **Duration:** 12m  **Frequency** 2/w for exercise  **No. contacts:** NR  **Group/individual:** NR  **Medium:** NR  **Facilitator:** Exercise physiology study personnel  **Comparison group care:** Diet only | **Diet:** balanced diet focused on low-density food intake  **PA:** Two exercise groups were scheduled to train 2/w- one group was aerobic and the other was resistance training. Both exercise groups were also assigned the same diet regimen as the diet-only group.  **Behavioral:** NR | **BMI:** y  **FPG:** y  **A1C:** n  **Insulin:** y  **Cholesterol:** n  **LDL:** n  **HDL:** n  **TG:** n  **SBP:** n  **DBP:** n  **Other:** Intra-abdominal adipose tissue, | **Sampling method:** Recruited from community  **Jadad Score:** 0, 0, 0, C  **Randomization procedure:** NR  **Allocation** **concealment:** Unclear  **Attrition (%):** 0  **Blinding pt:** NR  **Blinding assessor:** NR  **Blinding provider:** NR  **BL comparable:** ND | |
| Fogelholm et al, 2000 | **Follow-up :** 24m  **No. study arms:** 3  **Setting:** Finland  **Number:** 82 | **Age (y): R**ange:30-45  **Sex** (%F): 100  **Health condition**: Obese, premenopausal, otherwise healthy but don’t exercise  **BL wt,:** 92kg  **BMI:** 34.0 kg/m^2^  **BL FPG**: 5.1 mmol/L  **BL A1C:** NR  **BL insulin**: 12.7 µU/ml | **Duration:** 24m  **Frequency:** group1: walk 2-3h/w; group 2: walk 4-6h/w  **No. contacts:** Unclear  **Group/individual:** Group  **Medium:** In-person  **Facilitator: N**utritionist  **Comparison group care:** UC (Not encouraged to exercise) | **Diet:** very low energy for weight loss but only first 12w – all subjects participated  **PA:** walking programs in which pedometers were used  **Behavioral:** monitored high-risk situations for overeating | **BMI:** y  **FPG:** y  **A1C:** n  **Insulin:** y  **Cholesterol:** y  **LDL:** n  **HDL:** y  **TG:** y  **SBP:** y  **DBP:** y  **Other:** WC, body wt, metabolic syndrome | **Sampling method**: Recruited from ads  **Jadad Score:** 2, 0, 1, C  **Randomization procedure:** Random permuted blocks within strata  **Allocation** **concealment:** Unclear  **Attrition (%):** 9.8  **Blinding pt:** NR  **Blinding assessor:** NR  **Blinding provider:** NR  **BL comparable:** ND | |
| Fonolla et al. 2009 | **Follow-up**: 12  **No. study arms**: 3  **Setting**: Community, metropolitan Granada, Spain  **Number**: 297 | **Age (y):** 46.0  **Sex (%F):** 15.5  **Health condition**: Moderate CVD risk  **BL wt, BMI**: 28.8 kg/m^2^  **BL FPG**: 5.6 mmol/L  **BL A1C**: NR  **BL insulin**: NR | **Duration**: 12m  **Frequency:** NR  **No. contacts:** ongoing milk consumption at home  **Group/individual:** Individual  **Medium**: NR  **Facilitator**: NR  **Comparison group care**: Provided enriched, semi-skimmed or skimmed milk for one year. | **Diet**: Enriched milk had pUFA, oleic acids, vitamins A, D, E, B6 and folic acid.  **PA**: NR  **Behavioral**: NR | **BMI**: y  **FPG**: y  **A1C**: n  **Insulin**: n  **Cholesterol**: y  **LDL**: y  **HDL**: y  **TG**: n  **SBP**: n  **DBP**: n  **Other**: Triacylglycerol, CRP, homocysteine | **Sampling method**: Recruited from community  **Jadad Score**: 0, 1, 1, C  **Randomization procedure**: NR  **Allocation concealment**: Unclear  **Attrition (%):** 14.8  **Blinding pt**: Yes  **Blinding assessor**: Yes  **Blinding provider**: Yes  **BL comparable**: ND | |
| Frank et al, 2005 | **Follow-up:** 12m  **No. study arms:** 2  **Setting:** Seattle, WA  **Number:** 173 | **Age (y):** 60.7  **Sex** (%F): 100  **Health condition**: NR  **BL wt,** NR  **BMI:** 30.4 kg/m^2^  **BL FPG**: 5.4 mmol/L  **BL A1C:** NR  **BL insulin**: 17.9 µU/ml | **Duration:** 12m  **Frequency:** 5/w at supervised facility/w during months 1-3 and exercise 2d/w at home. For months 4-12, 1/w at the facility and 4/w at home  **No. contacts:** NR  **Group/individual:** Both  **Medium:** In-person  **Facilitator:** NR  **Comparison group care:** 1/w 45 min stretching class | **Diet:** NR  **PA:** Intervention group did moderate intensity exercise at least 45 mins 5/w 3x at supervised facility/w during months 1-3 and exercise 2d/w at home. For months 4-12 they attended 1x/w at the facility and 4x/w at home  **Behavioral:** NR | **BMI:** n  **FPG:** y  **A1C:** n  **Insulin:** y  **Cholesterol:** n  **LDL:** n  **HDL:** n  **TG:** y  **SBP:** n  **DBP:** n  **Other:** HOMA, leptin | **Sampling method**: Mass mailings and media  **Jadad: Score** 0 0 0 C  **Randomization procedure:** NR  **Allocation** **concealment:** Unclear  **Attrition (%):** 1.7  **Blinding pt:** NR  **Blinding assessor:** NR  **Blinding provider:** NR  **BL comparable:** ND | |
| Groeneveld et al. 2008&2010 | **Follow-up**: 12m  **No. study arms:** 2  **Setting**: Dutch construction industry, Amsterdam, The Netherlands  **Number**: 816 | **Age (y):** 46.6  **Sex (%F)**: 0  **Health condition**: overweight/obese male construction workers with insufficient PA and at high risk for CVD and HT;  **BL wt.:** 92.6 kg  **BMI:** 28.5 kg/m^2^  **BL FPG**: NR  **BL A1C:** 5.7%  **BL insulin**: NR | **Duration:** 12m  **Frequency:** Regular scheduled contacts and 6-months follow-up  **No. of contacts:** 10  **Group/individual:** Individual  **Medium:** In-person  **Facilitator:** Trained volunteer occupational physicians and nurses  **Comparison group care:** UC | **Diet** –Adopting individualized healthy eating via motivational interviews  **PA:** PA via motivational interviews  **Behavioral:** Individualized counselling via a series of motivational interviews on participant’s attitude, self-efficacy, and social influence. | **BMI:** y  **FPG:** n  **A1C:** y  **Insulin:** n  **Cholesterol:** n  **LDL:** n  **HDL:** y  **TG:** n  **SBP:** y  **DBP:** y  **Others**: Cardiorespiratory fitness, smoking status, PA | **Sampling method:** Screenings program  **Jadad Score:** 2,0,1,C  **Randomization procedure:** Using random allocation software  **Allocation** **concealment:** Adequate  **Attrition (%):** 27.6  **Blinding pt.:** No  **Blinding assessor:** Yes  **Blinding provider:** No  **BL comparable:** ND | |
| Heshka et al. 2003 | **Follow-up**: 24m  **No. study arms:** 2  **Setting**: 6 US clinical centers across the nation  **Number**: 423 | **Age (y):** 44.5  **Sex(%F)**: 84.6  **Health condition**: overweight men and women (BMI – 27 to 40); included those who needed weight reduction for their health problems  **BL wt:** 93.6 kg  **BMI:** 33.7 kg/m^2^  **BL FPG**: 5.0 mmol/L  **BL A1C:** NR  **BL insulin:** 18 µU/ml | **Duration:** 24m  **Frequency**: daily  **No. contacts:** weekly  **Group/individual**: groups  **Medium**: In-person  **Facilitator:** a dietitian, previous Weight Watchers program graduates  **Comparison group care**: UC | **Diet:** a nutritionally balanced, moderate-deficit diet designed for weight loss up to 0.9 kg/w.  **PA:** An activity plan according to current NIH guidelines; Attendance at free Weight Watchers sessions  **Behavioral:** A behavior modification plan as part of programs for the intervention group, including weekly support group meetings | **BMI:** y  **FPG:** y  **A1C:** n  **Insulin:** y  **Cholesterol:** y  **LDL:** n  **HDL:** y  **TG:** y  **SBP:** y  **DBP:** y  **Others:** Body fat, waist circumference, | **Sampling method:** Clinic-based recruitment;  **Jadad Score:** 2,0,1, C  **Randomization procedure:** Using random number table  **Allocation concealment:** Adequate  **Attrition(%):** 27  **Blinding pt:** NR  **Blinding assessor:** NR  **Blinding provider:** NR  **BL comparable:** ND | |
| Imayama et al. 2013  Foster-Schubert et al. 2012 Mason et al. 2011&2013 | **Follow-up**: 12m  **No. study arms:** 4  **Setting**: Fred Hutchinson Cancer research Center, Seattle, WA  **Number**: 439 | **Age (y):** 58.0  **Sex(%F)**: 100  **Health condition**: Postmenopausal women  **BL wt:** 83.6 kg  **BMI:** 30.9 kg/m^2^  **BL FPG**: 5.4 mmol/L  **BL A1C:** NR  **BL insulin:** 12.9 µU/ml | **Duration:** 12m  **Frequency**: 1/w in 6m, 1/m in 6m  **No. contacts:** 30  **Group/individual**: Both  **Medium**: In-person and e-mail  **Facilitator:** Dietitian  **Comparison group care:** UC | **Diet:** caloric intake of 1200-2000 kcal/d, ≤30% fat, 10% weight loss by week 24; with weight maintenance thereafter  **PA:** 45min/d, 5d/w of moderate-to-vigorous intensity PA for 12m; 3 supervised sessions/w at facility, 2d/w at home till 70-80% of maximal HR  **Behavioral:** NR | **BMI:** y  **FPG:** y  **A1C:** n  **Insulin:** y  **Cholesterol:** n  **LDL:** n  **HDL:** y  **TG:** n  **SBP:** n  **DBP:** n  **Others:** NR | **Sampling method:** Clinic-based recruitment;  **Jadad Score:** 2,1,1, A  **Randomization procedure:** Computerized randomization  **Allocation concealment:** Adequate  **Attrition(%):** 9.1  **Blinding pt:** No  **Blinding assessor:** Yes  **Blinding provider:** Yes  **BL comparable:** ND | |
| Juul et al. 2016 | **Follow-up**: 12m  **No. study arms:** 2  **Setting**: Community, Denmark  **Number**: 127 | **Age (y):** median: 58  **Sex(%F)**: 68.6  **Health condition**: High risk for DM  **BL wt: median:** 89 kg for IG; 85 kg for CG  **BMI:** median: 31 for IG; 30 kg/m^2^ for CG  **BL FPG**: NR  **BL A1C:** 5.8%  **BL insulin:** NR | **Duration:** 12m  **Frequency:** 4/5w in the first 5w  **No. of contacts:** 6  **Group/individual:** Group  **Medium:** In-person  **Facilitator:** Dietitian, occupational therapist  **Comparison group care:** UC | **Diet:** Weight reduction>5%, total-fat intake <30%, saturated-fat intake <10%, fibre-intake≥15g/1000kcal  **PA:** increase PA  **Behavioral:** NR Lifestyle change based on transformative learning, health literacy theory, and dimensions of health knowledge and action competence. | **BMI:** y  **FPG:** n  **A1C:** y  **Insulin: n**  **Cholesterol: y**  **LDL:** y  **HDL:** y  **TG:** n  **SBP:** y  **DBP:** y  **Others:** SF-12 | **Sampling method:** From a referral;  **Jadad Score:** 2,0,1, B  **Randomization procedure:** used sealed envelopes, randomization  **Allocation concealment:** Adequate  **Attrition(%):** 15.0  **Blinding pt:** No  **Blinding assessor:** NR  **Blinding provider:** NR  **BL comparable:** ND | |
| Kanaya et al. 2012 Delgadillo et al. 2010 | **Follow-up**: 12m  **No. study arms:** 2  **Setting**: Community in Berkeley, Oakland, Richmond, CA  **Number**: 238 | **Age (y):** 56.5  **Sex(%F)**: 73.5  **Health condition**: with risk for DM  **BL wt:** 79.7 kg  **BMI:** 30.0 kg/m^2^  **BL FPG**: 5.2 mmol/L  **BL A1C:** NR  **BL insulin:** NR | **Duration:** 12m  **Frequency**: NR  **No. contacts:** 19  **Group/individual**: Both  **Medium**: In-person and phone  **Facilitator:** Counselor  **Comparison group care:** UC | **Diet**: Live Well, Be Well, 12 telephone counseling calls to educate healthy diet  **PA**: Live Well, Be Well, 12 telephone counseling calls to educate PA  **Behavioral**: Self-selected and attainable goal-setting and action plans to enhance self-efficacy, and motivation | **BMI:** y  **FPG:** y  **A1C:** n  **Insulin:** n  **Cholesterol:** n  **LDL:** y  **HDL:** y  **TG:** y  **SBP:** y  **DBP:** n  **Others:** Caloric intake and PA | **Sampling method:** Community-based  **Jadad Score:** 2,1,1, A  **Randomization procedure:** Using stratum-specific sequential number  **Allocation concealment:** Adequate  **Attrition(%):** 12.2  **Blinding pt:** No  **Blinding assessor:** Yes  **Blinding provider:** Yes  **BL comparable:** ND | |
| Kanaya et al 2014 | **Follow-up**: 12m  **No. study arms:** 2  **Setting**: Community in San Francisco, San Diego, CA  **Number**: 180 | **Age (y):** 55.0  **Sex(%F)**: 72.0  **Health condition**: MetS and HT  **BL wt:** 93.9 kg  **BMI:** 34.3 kg/m^2^  **BL FPG**: 5.8 mmol/L  **BL A1C:** 5.9%  **BL insulin:** 27.5 µU/ml | **Duration:** 12m  **Frequency**: 2/w 3m, 1/w 3m, 1/m 6m  **No. contacts:** 42  **Group/individual**: Both  **Medium**: In-person and  **Facilitator:** Instructors  **Comparison group care:** Practicing stretching | **Diet**: NR  **PA**: Restorative yoga group practices lyengar poses in a group setting 2/w 3m, 1/w 3m, and 1/m 6m, at home for 3 30min/w;  **Behavioral**: NR | **BMI:** y  **FPG:** y  **A1C:** y  **Insulin:** y  **Cholesterol:** y  **LDL:** y  **HDL:** y  **TG:** y  **SBP:** y  **DBP:** y  **Others:** Caloric intake and PA | **Sampling method:** Clinic and community-based  **Jadad Score:** 2,0,1, C  **Randomization procedure:** Using stratum-specific sequential number  **Allocation concealment:** Adequate  **Attrition(%):** 21.1  **Blinding pt:** No  **Blinding assessor:** No  **Blinding provider:** No  **BL comparable:** BMI different in BL | |
| Katula et al. 2010 & 2011 & 2013 | **Follow-up**: 24 m  **No. study arms**: 2  **Setting**: Forsyth County, NC, community  **Number**: 301 | **Age (y)**: 57.9  **Sex(%F)**: 58  **Health condition**: Pre-diabetes and overweight or obese  **BL wt:** 93.7 kg  **BMI**: 32.7 kg/m^2^  **BL FPG**: 5.9 mmol/L  **BL A1C**: NR  **BL insulin:** 16.7 µU/ml | **Duration**: 24 m  **Frequency**: 4 /m, plus 3 RD sessions in m 1-6; 2 /m in m 7-24  **No. of contacts**: 63  **Group/individual**: Both  **Medium**: In person & telephone  **Facilitators**: CHWs and RDs  **Comparison**: UC | **Diet**: Caloric intake: 1200-1800 kcal/d and to attain ~0.3kg WL/w for the first six m and thereafter, unless BMI falls below 20 kg/m2. Dietary topics included nutrition basics, energy balance, healthy eating.  **PA**: Fitness improvements achieved through moderate PA (≥180 min/w).  **Behavioral**: Self-efficacy, outcome expectations and incentives targeted, group dynamics and problem-solving. | **BMI**: y  **FPG**: y  **A1C**: n  **Insulin**: y  **Cholesterol**: n  **LDL**: n  **HDL**: n  **TG**: n  **SBP**: n  **DBP**: n  **Other**: WC, HOMA | **Sampling method**: Community-based 3-stage screen  **Jadad Score:**  1,0,0,C  **Randomization procedure**: Web-based data management system  **Allocation** **concealment**: No  **Attrition**: 12.6%  **Blinding pt**: No  **Blinding assessor**: No  **Blinding provider**: No  **BL comparable**: ND | |
| Kawano et al. 2009 | **Follow-up:** 12m  **No. study arms:** 2  **Setting:** Community**,** Japan  **Number:** 217 | **Age(y):** 60.9  **Sex(%F):** 66.5  **Health condition**: FPG: 100-140 mg/dL  **BL wt:** 58.2 kg  **BMI:** 23.7 kg/m^2^  **BL FPG**: 5.1 mmol/L  **BL A1C:** 5.1%  **BL insulin:** NR | **Duration 12**  **Frequency:** 1/m for diet; 2/w for exercise  **No. contacts:** 104  **Group/individual:** Group  **Medium:** In-person  **Facilitator:** Dietitian/sports physician  **Comparison group care:** UC | **Diet:** Monthly dietary classes for 5 months with a dietitian  **PA:** Ball exercises, exercise ergometers, aquatic exercise, tube exercises and stretching (2/w for at least 30 min in addition to their regular activities)  **Behavioral:** NR | **BMI:** y  **FPG:** y  **A1C:** y  **Insulin:** n  **Cholesterol:** y  **LDL:** y  **HDL:** y  **TG:** y  **SBP:** y  **DBP:** y  **Other:** NR | **Sampling method:** Population sample  **Jadad Score:** 1,0,1,C  **Randomization procedure:** Drawing lots  **Allocation** **concealment:** NR  **Attrition(%):** 27.2  **Blinding pt:** Yes  **Blinding assessor:** No  **Blinding provider:** No  **BL comparable:** ND | |
| Keogh et al. 2007 | **Follow-up**: 12m  **No. study arms**: 2  **Setting**: Outpatient  **Number** : 36 | **Age (y):** 48.7  **Sex (%F):** 68  **Health condition**: healthy  **BL wt, BMI**: 32.9 kg/m^2^  **BL FPG**: 5.9 mmol/L  **BL A1C**: NR  **BL insulin**: 14.8 µU/ml | **Duration**: 12m  **Frequency**: 2/w  **No. contacts**: NR  **Group/individual**: NR  **Medium**: In person  **Facilitator**: RD  **Comparison group care**: High carb 60% carb, 20% fat, 20% protein 4% sat fat, 5% PUFA, 7% MUFA, 40 g fiber | **Diet**: low carb – 33% carbs, 27% fat, 40% protein 7% sat fat, 6% PUFA, 13% MUFA, 26 g fiber  **PA**:  **Behavioral**: | **BMI**: y  **FPG**: y  **A1C**: n  **Insulin**: y  **Cholesterol**: y  **LDL**: y  **HDL**: y  **TG**: y  **SBP**: y  **DBP**: y  **Other**: CRP | **Sampling method**: Recruited by newspaper ad  **Jadad Score**: 0,0,1,C  **Randomization procedure**: NR  **Allocation concealment**: Unclear  **Attrition (%):** 30.6  **Blinding pt**: No  **Blinding assessor**: No  **Blinding provider**: No  **BL comparable**: ND | |
| Lawton et al. 2009 | **Follow-up**: 24m  **No. study arms:** 2  **Setting**:  17 primary care practices, Wellington, New Zealand;  **Number**: 1089 | **Age(y):** 58.9  **Sex(%F)**: 100  **Health condition**: Physically inactive women  **BL wt.:** 72.9 kg**,**  **BMI:** 29.2 kg/m^2^  **BL FPG**: 5.0 mmol/L  **BL A1C:** 5.5%  **BL insulin:** 6.9 µU/ml | **Duration:** 24m  **Frequency:** 1/w  **No. contacts:** An initial 7-13 min-counseling plus six 15-min-follow-up calls  followed by Monthly  **Group/individual**: Individual  **Medium**: In-person  **Facilitator**: A community-based exercise facilitator  **Comparison: usual care**: UC | **Diet:** NR  **PA:** Increase PA; moderate intensity aerobic exercise of 150 min/w ; 7-13 min initial counseling; phone support for 3m (15-min- five calls); a 30-min visit at 6m; Assessed by a questionnaire; 7-day PA log and energy expenditure  **Behavioral:** Increase PA using motivational interview techniques | **BMI:** y  **FPG:** y  **A1C:** y  **Insulin:** y  **Cholesterol:** y  **LDL:** n  **HDL:** y  **TG:** n  **SBP:** y  **DBP:** y  **Other**: WC, quality of life scores; energy expenditure | **Sampling method:** Recruited from clinics  **Jadad Score:** 2,2,1,A  **Randomization procedure:** Computer generated numbers;  **Allocation** **concealment:** Adequate  **Attrition(%):** 7.4  **Blinding pt.:** Yes  **Blinding assessor:** Yes  **Blinding provider:** No  **BL comparable:** ND | |
| Lim et al. 2010 | **Follow-up**: 12m  **No. study arms:** 4  **Setting**: Adelaide University, Adelaide, Australia  **Number**: 113 | **Age(y):** 47  **Sex(%F)**: 82.3  **Health condition**: Non-diabetic overweight/obese volunteer with 1 or more CV risk factors  **BL wt.:** 89.8 kg  **BMI:** 32.0 kg/m^2^  **BL FPG**: 5.4 mmol/L  **BL A1C:** NR  **BL insulin:** 9.1 µU/ml | **Duration:** 12m  **Frequency:** Visits at Month 3, 6, 9, 12, and 15.  **No. contacts:** 11  **Group/individual**: Individual  **Medium**: In-person  **Facilitator**: A qualified dietitian  **Comparison group care:** UC | **Diet**: 3 isocaloric diets: (1) very low carb [60% fat, 4% carb]; (2) very low fat [10% fat, 70% carb]; high unsaturated fat [30% fat, 50% carb];  **PA:** NR  **Behavioral:** Subjects were advised to adhere to their respective allocated diet. | **BMI:** y  **FPG:** y  **A1C:** n  **Insulin:** y  **Cholesterol:** y  **LDL:** y  **HDL:** y  **TG:** y  **SBP:** y  **DBP:** y  **Others:** C reactive protein, homocysteine, | **Sampling method:** Recruited by ads  **Jadad Score:** 1,0,1,C  **Randomization procedure:** NR  **Allocation** **concealment:** Unclear  **Attrition(%):** 38.9  **Blinding pt.:** NR  **Blinding assessor:** NR  **Blinding provider:** NR  **BL comparable:** ND | |
| Lombard et al. 2010 | **Follow-up**: 12m  **No. study arms:** 2  **Setting**: An urban community setting, Melbourne, elementary school environment; Australia;  **Number**: 250 | **Age(y):** 40.4  **Sex(%F)**: 100  **Health condition**: Women with young children, regardless of their BMI values  **BL wt:** 74.0 kg,  **BMI:** 27.8 kg/m^2^  **BL FPG**: 4.6 mmol/L  **BL A1C:** NR  **BL insulin:** NR | **Duration:** 12m  **Frequency:** 1/w at Week 1, 2, 3, and 16.  **No. contacts:** 17  **Group/individual**: groups  **Medium**: In-person  **Facilitator**: Dietitian  **Comparison** **group care**: UC | **Diet:** NR  **PA:** encouraged to join walking groups or to walk with friends; a pedometer provided.  **Behavioral:** Included goal setting, self-monitoring, social support, problem solving, & relapse prevention; also focused on behavior change skills related to diet and PA; | **BMI:** y  **FPG:** y  **A1C:** n  **Insulin:** n  **Cholesterol:** y  **LDL:** y  **HDL:** y  **TG:** y  **SBP:** n  **DBP:** n  **Other**: WC, energy intake, energy expenditure | **Sampling method:** School-based recruitment;  **Jadad Score:** 2,2,1,A  **Randomization procedure:** Computer generated numbers;  **Allocation** **concealment:** Adequate  **Attrition(%):** 14.0  **Blinding pt:** Yes  **Blinding assessor:** Yes  **Blinding provider:** No  **BL comparable:** ND | |
| Ma et al. 2009 & 2013 | **Follow-up**: 15m  **No. study arms**: 3  **Setting**: Clinic, Los Altos, CA, primary care  **Number**: 241 | **Age (y)**: 52.9  **Sex(%F)**: 47.0  **Health condition**: Overweight and pre-DM and/or MetS  **BL wt:** 93.8 kg  **BMI**: 32.0 kg/m2  **BL FPG**: 5.6 mmol/L  **BL A1C**: NR  **BL insulin:** NR | **Duration**: 15m  **Frequency**: Coach-led: 1/w in months 1-3;  Self-directed: ½w  **No. of contacts**: ~30  **Group/individual**: Group  **Medium**: In person or DVD, plus email  **Facilitators**: RD & lifestyle coach  **Comparison**: UC | **Diet**: 7% weight loss through fat and calorie reduction and healthier food choices.  **PA**: 7% weight loss through 150 min/w of moderate PA, increasing to 60 min/d if weight loss goal not achieved.  **Behavioral**: Goal-setting, skill building and self monitoring and building self efficacy, positive outcome expectations, and self management skills, | **BMI**: y  **FPG**: y  **A1C**: n  **Insulin**: n  **Cholesterol**: y  **LDL**: y  **HDL**: y  **TG**: y  **SBP**: y  **DBP**: y  **Other**: WC | **Sampling method**: By 2-step screening  **Jadad Score:**  2,0,1,C  **Randomization procedure**: Computerized algorithm  **Allocation** **concealment**: Adequate  **Attrition(%)**: 8.3  **Blinding pt**: No  **Blinding assessor**: Yes  **Blinding provider**: No  **BL comparable**: ND | |
| Marrero et al. 2016 | **Follow-up**: 12m  **No. study arms**: 2  **Setting**: Community, Indianapolis IN  **Number**: 225 | **Age (y)**: 52.0  **Sex(%F)**: 84.4  **Health condition**: pre-DM  **BL wt:** 100.5 kg  **BMI**: 36.8 kg/m2  **BL FPG**: 6.0 mmol/L  **BL A1C**: 5.8%  **BL insulin:** NR | **Duration**: 12m  **Frequency**: 1/w  **No. of contacts**: NR  **Group/individual**: Group  **Medium**: In-person  **Facilitators**: Trained group leaders  **Comparison**: UC | **Diet**: Modified DPP model with modest weight loss (5-10%) via caloric restriction, dietary modification  **PA**: moderate PA, **Behavioral**: self-monitoring of weight, stimulus control, and relapse prevention. | **BMI:** y  **FPG:** y  **A1C:** y  **Insulin:** n  **Cholesterol:** y  **LDL:** n  **HDL:** y  **TG:** n  **SBP:** y  **DBP:** y  **Other:** body weight %, | **Sampling method:** Recruited from screening  **Jadad Score:** 2,0,1,B  **Randomization procedure: A computer-generated** randomization list  **Allocation** **concealment:** Unclear  **Attrition (%):** 22.2  **Blinding pt:** No  **Blinding assessor:** NR  **Blinding provider:** NR  **BL comparable:** ND | |
| Marsh et al. 2010 | **Follow-up:**  12m  **No. study arms:** 2  **Setting:** Sydney, Australia  **Number** 96 | **Age (y):** 30.2  **Sex** (%F): 100  **Health condition**: Polycystic ovarian syndrome  **BL wt, BMI:** 34.5 kg/m^2^  **BL FPG**: 4.8 mmol/L  **BL A1C:** NR  **BL insulin:** 15.6 µU/ml | **Duration:** 12m  **Frequency 1/w** fortnightly for 4-6 visits, 2-4w thereafter  **No. contacts:** Varied  **Group/individual:** Individual  **Medium:** In person, phone or email  **Facilitator:** Accredited practicing dietician  **Comparison group care:** Same diet plan, but with high GI foods instead of low GI | **Diet:** Reduced energy, low-fat, low saturated fat, moderate-to –high fiber with similar macronutrient density, low GI carbs. Menu plans, shopping lists, key carb foods, newsletter sent out to increase compliance.  **PA:** Both groups informed of the benefits of PA and encouraged to exercise.  **Behavioral:** NR | **BMI:** y  **FPG:** y  **A1C:** n  **Insulin:** y  **Cholesterol:** y  **LDL:** y  **HDL:** y  **TG:** y  **SBP:** n  **DBP:** n  **Other:** body fat %, | **Sampling method:** Recruited from community  **Jadad Score:** 1,2,1,A  **Randomization procedure:** Using alternating order  **Allocation** **concealment:** Unclear  **Attrition (%):** 49.0  **Blinding pt:** Yes  **Blinding assessor:** Yes  **Blinding provider:** No  **BL comparable:** ND | |
| Mason et al 2016 | **Follow-up:**  12m  **No. study arms:** 2  **Setting:** Community, San Francisco, CA  **Number** 194 | **Age (y):** 47.0  **Sex** (%F): 78.0  **Health condition**: Obesity  **BL wt,** NR  **BMI:** 35.5 kg/m^2^  **BL FPG**: 4.8 mmol/L  **BL A1C:** NR  **BL insulin:** NR | **Duration:** 5.5m  **Frequency:** 1/w for 12w, ½w for 3w, ¼w for the last one  **No. of contacts:** 16  **Group/individual:** Group  **Medium:** In-person  **Facilitator:** Dietician  **Follow-up interval:** 12m **Comparison group care:** Same diet-exercise program without mindfulness | **Diet:** Diet-exercise program with mindfulness training, focusing on modest caloric reduction by 500 calories.  **PA:** Bicycling, swimming, strength training, and walking **Behavioral:** Focusing on mind training, stress reduction. | **BMI:** y  **FPG:** y  **A1C:** n  **Insulin:** n  **Cholesterol:** n  **LDL:** n  **HDL:** n  **TG:** n  **SBP:** n  **DBP:** n  **Other:** n | **Sampling method:** Recruited from community  **Jadad Score:** 2,0,1,B  **Randomization procedure:** A computer-generated random allocation sequence  **Allocation** **concealment:** Unclear  **Attrition (%):** 23.2  **Blinding pt:** No  **Blinding assessor:** No  **Blinding provider:** No  **BL comparable:** ND | |
| McAuley et al. 2005 | **Follow-up:** 12m  **No. study arms:** 3  **Setting:** Otago, New Zealand  **Number:** 93 | **Age (y):**  (range:30-70y)  **Sex** (%F): 100  **Health condition**: Overweight insulin resistant women  **BL wt, BMI:** 35.0 kg/m^2^  **BL FPG**: 5.1mmol/L  **BL A1C:** NR  **BL insulin:** 13.9 µU/ml | **Duration:** 12m  **Frequency:** 1/w  **No. contacts:** 17  **Group/individual:** NR  **Medium:** In-person  **Facilitator:** NR  **Comparison group care:** 6 servings of breads and cereals, preferably whole grains, 3 servings vegetables, 2 servings fruit, 2 servings low fat milk, one serving of lean meat, | **Diet:** 20g of carbs daily for 1^st^ 2w, carb intake increased by 5g/d a week for weeks 3-8 up to 50g daily by week 8. Weeks 8-16 5g/d increase per week continued;  total energy, 40% from low GI carbs, 30% from protein and 30% from fat, primarily monounsaturated.  **PA:**  Advised to participate in 30 min of any activity 5 d/w  **Behavioral:** NR | **BMI:** y  **FPG:** y  **A1C:** n  **Insulin:** y  **Cholesterol:** y  **LDL:** y  **HDL:** y  **TG:** y  **SBP:** y  **DBP:** y  **Other:** WC, fat mass, fat-free mass, 2-h glucose, CRP | **Sampling method:** Recruited by ads  **Jadad Score:** 2,0,1,C  **Randomization procedure:** NR  **Allocation** **concealment:** Adequate  **Attrition (%):** 18.3  **Blinding pt:** No  **Blinding assessor:** NR  **Blinding provider:** No  **BL comparable:** ND | |
| Mellberg et al. 2014 | **Follow-up:** 24m  **No. study arms:** 2  **Setting:** Community, Umea, Sweden  **Number:** 70 | **Age (y):**  59.9  **Sex** (%F): 100  **Health condition**: Postmenopausal women  **BL wt, BMI:** 32.7 kg/m^2^  **BL FPG**: 5.2 mmol/L  **BL A1C:** NR  **BL insulin:** 8.7 µU/ml | **Duration:** 24m  **Frequency:** 8/6m, then 4/18m  **No. contacts:** 12  **Group/individual:** Both  **Medium:** In-person  **Facilitator:** Dietitian  **Comparison group care:** Nordic Nutrition Recommendations group with 15 E% protein, 25-30 E% fat, and 55-60 E% carb. | **Diet:** Palaeolithic-type diet with 30% of energy intake (E%) from protein, 40 E% fat and 30 E% carb, and high intake of MUFA and PUFA **PA:**  NR  **Behavioral:** NR | **BMI:** y  **FPG:** y  **A1C:** n  **Insulin:** y  **Cholesterol:** y  **LDL:** y  **HDL:** y  **TG:** y  **SBP:** y  **DBP:** y  **Other:** WC, fat mass, body weight | **Sampling method:** Recruited by ads  **Jadad Score:** 2,0,1,C  **Randomization procedure:** block randomization  **Allocation** **concealment:** Adequate  **Attrition (%):** 30  **Blinding pt:** No  **Blinding assessor:** Yes  **Blinding provider:** No  **BL comparable:** ND | |
| Muto et al. 2001 | **Follow-up**: 18m  **No. study arms**: 2  **Setting**: Community, worksite, Japan  **Number**: 302 | **Age (y):** 42.5  **Sex (%F):** 0  **Health condition**: One abnormal lab or exam (either BMI, SBP, DBP, TC, TG, or FBG)  **BL wt**: 70.9 kg  **BMI**: 24.7 kg/m^2^  **BL FPG**: 5.6 mmol/L  **BL A1C**: NR  **BL insulin**: NR | **Duration**: 18m  **Frequency**: 2/yr  **No. contacts**: 4  **Group/individual**: Group  **Medium**: In-person  **Facilitator**: Physician, dietician, exercise trainer  **Comparison group care**; UC | **Diet**: 4 day group program with education on nutrition,  **PA**: 4 day group program with education on PA  **Behavioral**: 4 day group program with education on stress, CVD risk factors through lectures, training, individual counseling, and group discussion. | **BMI**: y  **FPG**: y  **A1C**: n  **Insulin**: n  **Cholesterol**: n  **LDL**: n  **HDL**: y  **TG**: y  **SBP**: y  **DBP**: y  **Other**: NR | **Sampling method**: Community screening  **Jadad Score**: 1, 0, 1, B  **Randomization procedure**: NR  **Allocation concealment**: NR  **Attrition (%):** 7.4  **Blinding pt**: No  **Blinding assessor**: No  **Blinding provider**: No  **BL comparable**: ND | |
| Narayan et al. 1998 | **Follow-up:** 12m  **No. study arms:** 2  **Setting:** Pima**,** Arizona  **Number:** 95 | **Age (y):** (range: 25-50y)  **Sex** (%F): 75.8  **Health condition**: Obese  **BL wt, BMI:** (Range:20.2-59.9 kg/m^2^)  **BL FPG**: (Range:4.2-6.5mmol/L)  **BL A1C:** (Range:4.5-6.35)  **BL insulin**: (Range: 24-137pm) | **Duration:** 12m  **Frequency:** 1/yr  **No. of contacts:** NR  **Group/individual:** Group  **Medium:** In-person  **Facilitator:** NR  **Comparison group care**: UC | **Diet:** Reduce fat and alcohol intake; increase fiber intake  **PA:** Walking, water aerobics, softball, volleyball, community farming and gardening, cleaning local cemetery  **Behavioral:** 1/w meetings reinforced home visits with behavioral techniques, modeling/role play | **BMI:** y  **FPG:** y  **A1C:** y  **Insulin:** y  **Cholesterol:** y  **LDL:** n  **HDL:** n  **TG:** y  **SBP:** y  **DBP:** y  **Other:** Energy, fat and fiber intake | **Sampling method:** Recruited from community  **Jadad Score:** 1,0.0.B  **Randomization procedure:** NR  **Allocation** **concealment:** Unclear  **Attrition (%):** 2  **Blinding pt:** No  **Blinding assessor:** No  **Blinding provider:** No  **BL comparable:** ND | |
| Nilsson et al. 1992 | **Follow-up:** 12m  **No. study arms:** 2  **Setting:** Dalby district, Sweden  **Number:** 94 | **Age (y):**  55.0  **Sex** (%F): NR  **Health condition**: none  **BL wt,** 81.4kg  **BMI:** NR  **BL FPG**: 5.0 mmol/L  **BL A1C:** NR  **BL insulin**: 17.6 µU/ml | **Duration:** 12 m  **Frequency:** monthly after 1m intensive, plus occasional outings  **No. contacts:** NR  **Group/individual:** Group  **Medium:** In person  **Facilitator:** Nurse, dietician, or physiotherapist  **Comparison group care:** UC | **Diet:** Total fat <30% of daily energy intake,. Daily cholesterol intake <200mg, daily dietary fiber intake 30g, eating cold water fish was recommended. Reduction in dietary salt recommended  **PA:** Four PA sessions/y and special PA sessions/2w  **Behavioral:** Sessions help participants adhere to the healthier lifestyle. | **BMI:** y  **FPG:** y  **A1C:** n  **Insulin:** y  **Cholesterol:** y  **LDL:** y  **HDL:** y  **TG:** y  **SBP:** y  **DBP:** y  **Other:** WHR, smoking index, | **Sampling method:** Recruited from community  **Jadad Score:** 1,0,0,C  **Randomization procedure:** NR  **Allocation** **concealment:** Unclear  **Attrition (%):** 8.5  **Blinding pt:** No  **Blinding assessor:** NR  **Blinding provider:** No  **BL comparable:** ND | |
| Nilsson et al. 2001 | **Follow-up:** 18m  **No. study arms:** 2  **Setting:** Helsingborg, Sweden  **Number:** 113 | **Age (y):**  49.7  **Sex** (%F): 60.9  **Health condition**: none  **BL wt, BMI:** 27.8 kg/m^2^  **BL FPG**: 4.9 mmol/L  **BL A1C:** NR  **BL insulin**: 8.7 µU/ml | **Duration:** 18m  **Frequency:** 16/yr plus individual counseling  **No. contacts:** 72-78  **Group/individual:** Both  **Medium:** In person  **Facilitator:** Nurses, nutritionists, physiotherapists,  **Comparison group care:** UC | **Diet:** NR  **PA:** NR  **Behavioral:**  Individual counseling sessions covering weight reduction among the obese, improved diet and physical activity, stress management and smoking cessation. Program included lectures, discussions, videos, and outdoor activities. | **BMI:** y  **FPG:** y  **A1C:** n  **Insulin:** y  **Cholesterol:** y  **LDL:** y  **HDL:** y  **TG:** y  **SBP:** y  **DBP:** y  **Other:** WHR | **Sampling method:** Recruited from community  **Jadad Score:** 1,0,1,C  **Randomization procedure:** NR  **Allocation** **concealment:** Unclear  **Attrition (%):** 18.6  **Blinding pt:** No  **Blinding assessor:** NR  **Blinding provider:** No  **BL comparable:** ND | |
| Ockene et al. 2012  Merriam et al. 2009 | **Follow-up:** 12m  **No. study arms:** 2  **Setting:** Massachusetts  **Number:** 312 | **Age (y):** 52.0  **Sex** (%F): 74.4  **Health condition**: High Risk  **BL wt, BMI:** 33.9 kg/m^2^  **BL FPG**: 5.8 mmol/L  **BL A1C:** NR  **BL insulin:** 20.0 µU/ml | **Duration:** 12m  **Frequency:** 1/yr for diet counseling; 1/w for PA  **No. of contacts:** 16  **Group/individual:** Both  **Medium:** In-person  **Facilitator:** NR  **Comparison group care:** UC | **Diet:** Increase whole grain intake, decrease intake of starchy, sodium, total and saturated fat, portions of carbs and refined carbs  **PA:** Increase walking by 4000 steps per day over baseline  **Behavioral:** Social Cognitive Theory to promote positive attitudes and increase self efficacy | **BMI:** y  **FPG:** y  **A1C:** y  **Insulin:** y  **Cholesterol:** n  **LDL:** n  **HDL:** n  **TG:** n  **SBP:** n  **DBP:** n  **Other:** HOMA-IR | **Sampling method:** Recruited from community  **Jadad Score:** 1, 1, 1 A  **Randomization procedure:** Using a randomized block design  **Allocation** **concealment:** Unclear  **Attrition (%):** 7.4  **Blinding pt:** NR  **Blinding assessor:** No  **Blinding provider:** No  **BL comparable:** ND |  |
| Poston et al. 2006 | **Follow-up:** 12m  **No. study arms:** 2  **Setting:** Houston area  **Number:** 250 | **Age (y):** 41.0  **Sex** (%F): 92.4  **Health condition**: Overweight or obese  **BL wt, BMI:** 36.1 kg/m^2^  **BL FPG**: 4.5 mmol/L  **BL A1C:** NR  **BL insulin:** NR | **Duration:** 12m  **Frequency:** 1/m  **No. contacts:** 13  **Group/individual:** Individual  **Medium:** In person  **Facilitator:** Nurse  **Comparison group care:** UC | **Diet:** 30% calories consumed daily from fat and encouraged to keep a food diary  **PA:** Encouraged to expend 200 cal/d in exercise and given suggestions on how to accomplish this,  **Behavioral:** C cognitive-behavioral methods: self-monitoring, stimulus control, cognitive restructuring and stress management skills. | **BMI:** y  **FPG:** y  **A1C:** n  **Insulin:** n  **Cholesterol:** y  **LDL:** y  **HDL:** y  **TG:** y  **SBP:** y  **DBP:** y  **Other:** None | **Sampling method:** Recruited prescreen questionnaires mailed  **Jadad Score:** 1,0,1,C  **Randomization procedure:** NR  **Allocation** **concealment:** Unclear  **Attrition (%):** 45.6  **Blinding pt:** No  **Blinding assessor:** No  **Blinding provider:** No  **BL comparable:** ND | |
| Potteiger et al. 2002&2003 | **Follow-up:** 16m  **No. study arms:** 2  **Setting:** Kansas  **Number:** 66 | **Age (y):**  NR  **Sex** (%F): 57.6  **Health condition**: none  **BL wt, BMI:** (range: 25-34.6 kg/m^2^)  **BL FPG**: 5.5 mmol/L  **BL A1C:** NR  **BL insulin:** 11.4 µU/ml | **Duration:** 16m  **Frequency:** 3-5 d/w  **No. contacts:** Unclear  **Group/individual:** NR  **Medium:** In person  **Facilitator:** NR  **Comparison group care:** UC (Continued with daily activities as normal) | **Diet:** Ad libitum diet: 30–35% fat, 45–55% carb, and 10 – 25% protein  **PA:** Supervised exercise for 9m. Exercise progressed from 3d/w at 65% of HRR for 30 min to 5d/w at 75% of HRR for 45 min across 16w and then remained at 5d/w at 75% of HRR for 45min across the remainder of the study **Behavioral:** NR | **BMI:** n  **FPG:** n  **A1C:** n  **Insulin:** n  **Cholesterol:** n  **LDL:** n  **HDL:** n  **TG:** n  **SBP:** n  **DBP:** n  **Other:** Glucose areas under the curve | **Sampling method:** Recruited from an ongoing study  **Jadad Score:** 1,0,0,C  **Randomization procedure:** NR  **Allocation** **concealment:** Unclear  **Attrition (%):** 10.1  **Blinding pt:** No  **Blinding assessor:** No  **Blinding provider:** No  **BL comparable:** ND | |
| Reid et al. 2014 | **Follow-up:** 12m  **No. study arms:** 2  **Setting:** Clinic, Ottawa, Canada  **Number:** 426 | **Age (y):** 51.5  **Sex** (%F): 61.3  **Health condition**: With coronary risk  **BL wt:** NR  **BMI:** 29.4 kg/m^2^  **BL FPG**: 5.1 mmol/L  **BL A1C:** NR  **BL insulin:** NR | **Duration:** 12m  **Frequency:** 1/w in 12w  **No. contacts:** 17  **Group/individual:** Both  **Medium:** In-person and phone  **Facilitator:** Educator  **Comparison group care:** UC | **Diet:** Counseling including heathy eating  **PA:** Counseling session including PA  **Behavioral**: Counseling session including face-to-face and phone, regarding goal-setting, barriers overcome | **BMI:** y  **FPG:** y  **A1C:** n  **Insulin:** n  **Cholesterol:** y  **LDL:** y  **HDL:** y  **TG:** y  **SBP: y**  **DBP:** y  **Other:** Caloric intake and PA | **Sampling method:** Clinic sample  **Jadad Score:** 2,0,1,B  **Randomization procedure:** Computer-generated sequence  **Allocation** **concealment:** Adequate  **Attrition (%):** 25.8  **Blinding pt:** No  **Blinding assessor:** No  **Blinding provider:** No  **BL comparable:** ND | |
| Rossner et al. 1997 | **Follow-up:** 12m  **No. study arms:** 3  **Setting:** Obesity Unit, Karolinska Hospital; Stockholm, Sweden  **Number:** 93 | **Age(y):** 41.0  **Sex(%F)**: 67.7  **Health condition**: Obese with BMI> 30 kg/m  **BL BMI:** 38.7 kg/m^2^  **BL FPG**: 5.2 mmol/L  **BL A1C:** NR  **BL insulin:** NR | **Duration:** 12 m  **Frequency:** 4/yr  **No. of contacts:** 4  **Group/individual:** Both  **Medium:** In-person  **Facilitator:** Research nurse & a dietician  **Comparison:** 3 groups—Group. 1= Nutrilett of 420 kcal/day;  Group. 2= VLCD 530 kcal/day;  Group. 3= LCD 880 kcal/day | **Diet:** Nutrilett group: a supplement to take w/ water over 5 meals w/ 1 vitamin & 1 fish-oil tab; VLCD and LCD had no food until week 7 and then began a “balanced hypocaloric diet” 1,600 kcal/d  **PA:** Walking was encouraged  **Behavioral:** VLCD and LCD groups had continuous behavioral monitoring w/ reinforcement of balanced diet instructions | **BMI:** y  **FPG:** y  **A1C:** n  **Insulin:** n  **Cholesterol:** y  **LDL:** n  **HDL:** n  **TG:** y  **SBP:** y  **DBP:** y  **Other:** Body fat %; waist-hip ratio; | **Sampling method:** Recruited from clinics  **Jadad Score:** 1, 0, 0, B  **Randomization procedure:** Randomly assigned  **Allocation concealment:** Concealed randomization from pts, but could not conceal dietary supplement due to labeling  **Attrition(%):** 38.7  **Blinding pt:** Yes  **Blinding assessor:** NR  **Blinding provider:** NR  **BL comparable:** ND | |
| Ryttig et al. 1997 | **Follow-up:** 28m  **No. study arms:** 3  **Setting:** Obesity Unit, Karolinska Hospital; Stockholm, Sweden  **Number:** 81 | **Age(y):** 42.5  **Sex(%F)**: 54.3  **Health condition**: obesity  **BL BMI:** 37.7 kg/m^2^  **BL FPG**: 5.5 mmol/L  **BL A1C:** NR  **BL insulin:** NR | **Duration:** 26m  **Frequency:** 1/w for 1^st^ m, ½w for 2^nd^ m, 1/m for 6m  **No. of contacts:** 25  **Group/individual:** Both  **Medium:** In-person  **Facilitator:** Research nurse & a dietician  **Comparison group care:** 2 groups. Initially, then 3groups—  Group. A= 1,600 kcal/d  Group. B + C= VLCD plus 420 kcal/d; | **Diet:** Group. A pts. 1,600 kcal/d. Other pts. In a combined VLCD (Group. B + C): 420 kcal/d of Nutrilett nutrition powder with a balanced diet. The VLCD pts. Were re-randomized to a balanced hypocaloric diet plus 239 kcal/d of nutrition powder, not others. All pts. In both VLCD groups. Took a multivitamin  **PA:** NR  **Behavioral:** NR | **BMI:** y  **FPG:** y  **A1C:** n  **Insulin:** n  **Cholesterol:** y  **LDL:** n  **HDL:** y  **TG:** y  **SBP:** y  **DBP:** y  **Other:** Body fat %; WHR | **Sampling method:** Recruited from clinics  **Jadad Score:** 1, 0, 1, B  **Randomization procedure:** NR  **Allocation concealment:** Unclear  **Attrition(%):** 4.9  **Blinding pt:** Yes  **Blinding assessor:** NR  **Blinding provider:** NR  **BL comparable:** ND | |
| Sartorelli et al. 2005 | **Follow-up:** 12m  **No. study arms:** 2  **Setting:** Primary healthcare setting in Sao Paulo, Brazil  **Number:** 104 | **Age(y):** 45.5  **Sex(%F)**: 79.8  **Health condition**: first-degree relative w/ type 2 DM  **BL BMI:** 28.7 kg/m^2^  **BL FPG**: 5.2 mmol/L  **BL A1C:** NR  **BL insulin:** NR | **Duration:** 12m  **Frequency:** 3/6m  **No. of contacts:** 6  **Group/individual**: Both  **Medium:** In-person  **Facilitator:** Research assistants & nutritionist  **Comparison group care:** UC | **Diet:** Received diet prescription by a nutritionist w/ a food exchange list  **PA:** 30 min of walking/d encouraged  **Behavioral:** NR | **BMI:** y  **FPG:** y  **A1C:** n  **Insulin:** n  **Cholesterol:** y  **LDL:** y  **HDL:** y  **TG:** y  **SBP:** y  **DBP:** y  **Other:** Health checks, anthropometric, dietary intake, & PA | **Sampling method:** Recruited through screening program  **Jadad Score:** 1, 0, 1, B  **Randomization procedure:** Randomized with stratification.  **Allocation concealment:** Adequate  **Attrition(%):** 31.7  **Blinding pt:** Yes  **Blinding assessor:** No  **Blinding provider:** No  **BL comparable:** ND | |
| Sattin et al. 2016 | **Follow-up:** 12m  **No. study arms:** 2  **Setting:** Community, Augusta, GA  **Number:** 604 | **Age(y):** 46.5  **Sex(%F)**: 83.0  **Health condition**: Overweight or obesity  **BL wt**: 98.7 kg  **BL BMI:** 35.7 kg/m^2^  **BL FPG**: 5.0 mmol/L  **BL A1C:** 5.8%  **BL insulin:** NR | **Duration:** 3m  **Frequency:** 1/w for 12w  **No. of contacts:** 12  **Group/individual:** Group  **Medium:** In-person  **Facilitator:** Health provider, church health advisors  **Comparison group care:** Health education | **Diet:** Fit Body and Soul (FBAS) program used modified DPP model to weight loss>7%  **PA:** Increase PA by at least 150 MET-min/w.  **Behavioral:** Stimulus control, goal setting, and problem solving. | **BMI:** y  **FPG:** y  **A1C:** y  **Insulin:** n  **Cholesterol:** n  **LDL:** n  **HDL:** n  **TG:** n  **SBP:** y  **DBP:** y  **Other:** SF-12 | **Sampling method:** Recruited from church  **Jadad Score:** 1, 0, 1, B  **Randomization procedure:** NR  **Allocation concealment:** Adequate  **Attrition(%):** 0  **Blinding pt:** No  **Blinding assessor:** Yes  **Blinding provider:** No  **BL comparable:** ND | |
| Simkin-Silverman et al. 1995&1998&2003  Kuller et al. 2001&2006 | **Follow-up:** 54m  **No. study arms:** 2  **Setting:** Univ. of Pittsburgh, Allegheny Co., PA; presumed clinic  **Number:** 535 | **Age(y):** 47.0  **Sex(%F)**: 100  **Health condition**: Premenopausal women  **BL BMI:** 25.1 kg/m^2^  **BL FPG**: 5.4 mmol/L  **BL A1C:** NR  **BL insulin:** NR | **Duration:** 54m  **Frequency:** 15/20w  **No. of contacts:** at least 23x in 1^st^ yr.; 2-3x in years 2-4  **Group/individual**: Both  **Medium:** In-person, on phone, & e-mail  **Facilitator:** Trained nutritional & behavioral interventionists  **Comparison group care:** UC | **Diet:** Lowering intake of total fat to 25%, saturated fat to 7% and total cholesterol. To 100 mg/d; followed a 1,300 – 1,500 cal meal plan for 4w.  **PA:** Gradually increased PA to 1,000 kcal/w; emphasized walking  **Behavioral:** Problem-solving, stimulus control, goal setting, assertiveness training, relapse prevention, & cognitive techniques to maintain diet & exercise | **BMI:** y  **FPG:** y  **A1C:** n  **Insulin:** n  **Cholesterol:** y  **LDL:** y  **HDL:** y  **TG:** y  **SBP:** y  **DBP:** y  **Other:** Alcohol & cigarettes/d; WHR; calcium mg/d; calories/d; % fat/d | **Sampling method:** Recruited from registered voters  **Jadad Score:** 1, 1, 1, A  **Randomization procedure:** Using randomization sequence  **Allocation concealment:** Adequate  **Attrition(%):** 2.8  **Blinding pt:** Yes  **Blinding assessor:** Yes  **Blinding provider:** NR  **BL comparable:** ND | |
| Siu et al 2015 | **Follow-up:** 12m  **No. study arms:** 2  **Setting:** Community, Hong Kong, China  **Number:** 182 | **Age(y):** 565.0  **Sex(%F)**: 74.2  **Health condition**: MetS  **BL BMI:** NR  **BL FPG**: 5.8 mmol/L  **BL A1C:** NR  **BL insulin:** NR | **Duration:** 12m  **Frequency:** 3/w for 12m  **No. of contacts:** 156  **Group/individual:** Group  **Medium:** In-person  **Facilitator:** yoga instructors  **Comparison group care:** UC | **Diet:** NR  **PA:** Three 60-min yoga sessions weekly for 1 year. **Behavioral:** NR | **BMI:** n  **FPG:** y  **A1C:** n  **Insulin:** n  **Cholesterol:** n  **LDL:** n  **HDL:** y  **TG:** y  **SBP:** y  **DBP:** y  **Other:** Daily caloric intake | **Sampling method:** Recruited from screening  **Jadad Score:** 2, 0, 1, B  **Randomization procedure:** Computer-generated random sequence  **Allocation concealment:** Unclear  **Attrition(%):** 33.4  **Blinding pt:** NR  **Blinding assessor:** NR  **Blinding provider:** NR  **BL comparable:** ND | |
| Staten et al. 2004. | **Follow-up:** 12m  **No. study arms:** 3  **Setting** Arizona  **Number:** 361 | **Age (y):** 57.2  **Sex** (%F): 100  **Health condition**: Breast/Cervical cancer  **BL wt, BMI:** 29. Kg/m^2^  **BL FPG**: 5.9 mmol/L  **BL A1C:** NR  **BL insulin:** NR | **Duration:** 12m  **Frequency:** 1/yr for diet counseling;  **No. of contacts:** NR  **Group/individual:** Individual  **Medium:** In-person  **Facilitator:** NR  **Comparison group care**: Counselling only | **Diet:** Dietary fat intake of less than 30%; saturated fat, 10% of total energy intake; dietary fiber intake of 3.4 g per MJ.  **PA:** Moderate to high intensity PA for 30 min at least 5d/w  **Behavioral:** Behavioral change techniques influencing motivation, action and maintenance | **BMI:** y  **FPG:** y  **A1C:** n  **Insulin:** n  **Cholesterol:** y  **LDL:** n  **HDL:** n  **TG:** n  **SBP:** y  **DBP:** y  **Other:** n | **Sampling method:** Recruited from clinics  **Jadad Score:** 1,0,0,B  **Randomization procedure:** NR  **Allocation** **concealment:** Unclear  **Attrition (%):** 33.4  **Blinding pt:** Yes  **Blinding assessor:** No  **Blinding provider:** No  **BL comparable:** ND | |
| Stefanick et al. 1998 | **Follow-up:** 12 m  **No. study arms:** 4  **Setting:** Univ. clinic setting in Stanford, CA  **Number:** 377 | **Age(y):** 52.1  **Sex(%F)**: 47.7  **Health condition**: Men & women (postmenopausal) w/ abnormal lipoprotein levels  **BL BMI:** 26.7 kg/m^2^  **BL FPG**: 5.3 mmol/L  **BL A1C:** NR  **BL insulin:** NR | **Duration:** 12m  **Frequency:** 3/w  **No. of contacts:** Diet & Diet + exercise groups.= 16; exercise only= ~ 38  **Group/individual:** Both  **Medium:** In-person, mail, & phone  **Facilitator:** RD; exercise staff  **Comparison group care:**  Group. 1= aerobic exercise  Group. 2= diet only  Group. 3= diet + exercise  Control= UC | **Diet:** Diet & Diet + exercise groups. Both followed the Ntl. Cholesterol Educ. Program. Step 2 diet; 12w.  **PA:** Exercise group. & Diet+ exercise group. Had an initial meeting. Followed by a 6w. phase of 3 sessions/w (treadmill); 6 to 8m. maintenance phase of brisk walking sessions  **Behavioral:** NR | **BMI:** n  **FPG:** y  **A1C:** n  **Insulin:** n  **Cholesterol:** y  **LDL:** y  **HDL:** y  **TG:** y  **SBP:** y  **DBP:** y  **Other:** Caloric intake, WHR, VO2 max | **Sampling method:** Recruited from a screening  **Jadad Score:** 1, 1, 1, A  **Randomization procedure:** Assignments made by computer  **Allocation concealment:** Adequate  **Attrition(%):** 27.0  **Blinding pt:** Yes  **Blinding assessor:** Yes  **Blinding provider:** NR  **BL comparable:** ND | |
| Tapsell et al. 2014 | **Follow-up:** 12 m  **No. study arms:** 2  **Setting:** Community, Wollongong, Australia  **Number:** 120 | **Age(y):** 48.9  **Sex(%F)**: 75.0  **Health condition**: Healthy adults  **BL BMI:** 30.0 kg/m^2^  **BL FPG**: 5.3 mmol/L  **BL A1C:** NR  **BL insulin:** 11.0 µU/ml (median) | **Duration:** 12m  **Frequency:** 6/yr  **No. of contacts:** 6  **Group/individual:** Individual  **Medium:** In-person  **Facilitator:** Dietitian  **Comparison group care:** Two diet groups | **Diet:** All requested to consume at least 5 servings of vegetables each day focusing on vegetables, fruit, grain foods, meat/fish/eggs/cheese, milk/yoghurt, and nuts/seeds/spreads/oils, but different in 0.5 vs 1.0 cup cooked; 1 vs 2.0 cups of raw, respectively.  **PA:** NR  **Behavioral:** NR | **BMI:** y  **FPG:** y  **A1C:** n  **Insulin:** y  **Cholesterol:** y  **LDL:** y  **HDL:** y  **TG:** y  **SBP:** n  **DBP:** n  **Other:** Caloric intake, WC, body weight | **Sampling method:** Recruited from ads  **Jadad Score:** 2, 0, 1, C  **Randomization procedure:** Using RALLOC in STATA  **Allocation concealment:** Adequate  **Attrition(%):** 22.5  **Blinding pt:** No  **Blinding assessor:** Yes  **Blinding provider:** NR  **BL comparable:** ND | |
| ter Bogt et al. 2009 | **Follow-up:** 12m  **No. study arms:** 2  **Setting:** 11 general practice locations in the northern Netherlands  **Number:** 457 | **Age(y):** 56.1  **Sex(%F)**: 57.9  **Health condition**: HT, dyslipidemia, or both  **BL BMI:** 29.6 kg/m^2^  **BL FPG**: 5.2 mmol/L  **BL A1C:** NR  **BL insulin:** NR | **Duration:** 12m  **Frequency:** 1/m in m1-3  **No. of contacts:** 7  **Group/individual:** Individual  **Medium:** In-person  **Facilitator:** Nurse practitioner  **Comparison group care:** UC | **Diet:** NR  **PA:** NR  **Behavioral:** Provided Computer-guided lifestyle counseling | **BMI:** y  **FPG:** y  **A1C:** n  **Insulin:** n  **Cholesterol:** y  **LDL:** y  **HDL:** y  **TG:** n  **SBP:** y  **DBP:** y  **Other:** WC | **Sampling method:** Recruited from clinics  **Jadad Score:** 1, 0, 1, C  **Randomization procedure:** Computer generated number  **Allocation concealment:** Adequate  **Attrition(%):** 9.0  **Blinding pt:** Yes  **Blinding assessor:** NR  **Blinding provider:** NR  **BL comparable:** ND | |
| Thompson et al. 2005 | **Follow-up (months):** 12 m  **No. study arms:** 3  **Setting:** Rochester MN, Mayo Clinic (hospital)  **Number** 90 | **Age (y):** 41.4  **Sex** (%F): 85.6  **Health condition**: Obese (BMI 30-40) participants  **BL wt, BMI:** 34.8 kg/m^2^  **BL FPG**: 5.2 mmol/L  **BL A1C:** NR  **BL insulin:** 11.0 µU/ml | **Duration (m)** 12 m  **Frequency:** Biweekly if adhered /weekly if non-adherent  **No. contacts:** NR  **Group/individual:** Individual  **Medium:** In-person  **Facilitator:** Dietitian  **Comparison group care:** Moderate calcium diet (standard) | **Diet:** Three diet interventions with a calorie deficit of 500 cal/d comparing a moderate calcium diet (800 mg) to a high calcium (1400 mg) diet and high calcium + high fiber diet.  **PA:** Exercise (e.g., brisk walking, treadmill, or exercise bicycle) at least 30 min four times a week.  **Behavioral:** NR | **BMI:** y  **FPG:** y  **A1C:** n  **Insulin:** y  **Cholesterol:** y  **LDL:** y  **HDL:** y  **TG:** y  **SBP**: n  **DBP:** n  **Other:** Leptin, body fat, trunk fat, WC | **Sampling method:** Recruited from population screen  **Jadad Score:** 2,0,1, C  **Randomization procedure:** Computer randomization  algorithm  **Allocation** **concealment:** Adequate  **Attrition (%):** 13.3  **Blinding pt:** No  **Blinding assessor:** No  **Blinding provider:** No  **BL comparable:** ND | |
| Tsai et al. 2010 | **Follow-up:** 18m  **No. study arms:** 2  **Setting:** Clinic  **Number:** 50 | **Age (y):** 49.4  **Sex** (%F): 88  **Health condition**: Healthy subjects  **BL wt, BMI:** 36.5 kg/m^2^  **BL FPG**: 5.5 mmol/L  **BL A1C:** NR  **BL insulin:** NR | **Duration:** 12 m  **Frequency:** 0, 2, 4, 8, 12, 16,  20, and 24 weeks  **No. contacts:** 8  **Group/individual:** Both  **Medium:** In-person  **Facilitator:** Medical assistants  **Comparison group care:** UC | **Diet:** Consume 1,200–1,500 kcal/d (if <250 lb) or 1,500–1,800 kcal/d (if ≥250 lb), to keep daily records of their food intake (in diaries provided).  **PA:** Gradually  increase their PA to 175 min/w (e.g., by walking).  **Behavioral:** Weight-Control Information provided | **BMI:** y  **FPG:** y  **A1C:** n  **Insulin:** n  **Cholesterol:** y  **LDL:** y  **HDL:** y  **TG:** y  **SBP:** y  **DBP:** y  **Other:** WC | **Sampling method**: Recruited from flyers  **Jadad Score:** 1, 0,0, C  **Randomization procedure:** Using sealed envelopes  **Allocation** **concealment:** Questionable  **Attrition (%):** 6.0  **Blinding pt:** No  **Blinding assessor:** No  **Blinding provider:** No  **BL comparable:** ND | |
| Vainionpaa et al. 2007 | **Follow-up:** 12m  **No. study arms:** 2  **Setting:** Clinic and home  **Number:** 120 | **Age (y):** (range:35-40y)  **Sex** (%F): 100  **Health condition**: Healthy subjects  **BL wt, BMI:** 25.3 kg/m^2^  **BL FPG**: 4.8 mmol/L  **BL A1C:** NR  **BL insulin:** 4.9 µU/ml | **Duration:** 12 m  **Frequency:** 3/w for 12m  **No. contacts:** 156  **Group/individual**: Both  **Medium:** In-person  **Facilitator:** Physiotherapist  **Comparison group care:** UC | **Diet:** NR  **PA:** Train for 10 min daily at home. PA was measured through an accelerometer  **Behavioral:** NR | **BMI:** y  **FPG:** y  **A1C:** n  **Insulin:** y  **Cholesterol:** y  **LDL:** y  **HDL:** y  **TG:** y  **SBP:** n  **DBP:** n  **Other**: Hip and WC, muscle and fat CSA, VO2max, ECG | **Sampling method**: Recruited from community  **Jadad Score:** 2, 0, 0, C  **Randomization procedure:** Randomly sequenced  **Allocation** **concealment:** Questionable  **Attrition (%):** 33.3  **Blinding pt:** No  **Blinding assessor:** No  **Blinding provider:** No  **BL comparable:** ND | |
| Vetter et al. 2013 Wadden et al. 2011 | **Follow-up:** 24m  **No. study arms:** 3  **Setting:** Clinic, Pennsylvania  **Number:** 390 | **Age (y):** 51.5  **Sex** (%F): 79.7  **Health condition**: MetS  **BL wt:** 107.7 kg  **BMI:** 38.5 kg/m^2^  **BL FPG**: 5.8 mmol/L  **BL A1C:** NR  **BL insulin:** 13.5 µU/ml | **Duration:** 24 m  **Frequency:** 1/m counseling session, 1/3m primary care provider visit  **No. contacts:** NR  **Group/individual**: Both  **Medium:** In-person  **Facilitator:** Primary care provider  **Comparison group care:** UC | **Diet:** A balance diet: 1200-1500 kcal/d for pts <113.6kg; 1500-1800 kcal/d for pts≥113.6kg. 15-20% from protein, 20-35% from fat  **PA:** Increasing PA to 180min/w, provided pedometer.  **Behavioral**: DPP-like lesson | **BMI:** y  **FPG:** y  **A1C:** n  **Insulin:** y  **Cholesterol:** y  **LDL:** y  **HDL:** y  **TG:** y  **SBP:** y  **DBP:** y  **Other:** NR | **Sampling method**: Referral from clinic  **Jadad Score:** 2, 0, 1, C  **Randomization procedure:** Computer-generated algorithm  **Allocation** **concealment:** NR  **Attrition (%):** 13.8  **Blinding pt:** No  **Blinding assessor:** No  **Blinding provider:** No  **BL comparable:** ND | |
| Von Thiele Schwarz et al. 2008 | **Follow-up:** 12m  **No. study arms:** 3  **Setting:** Worksite  **Number:** 195 | **Age (y):** 46.6  **Sex** (%F): 100  **Health condition**: Healthy women  **BL wt, BMI:** NR  **BL FPG**: 5.0 mmol/L  **BL A1C:** 4.4%  **BL insulin:** NR | **Duration:** 12 m  **Frequency:** Daily  **No. contacts:** 3  **Group/individual:** Individual  **Medium:** In –person  **Facilitator:** Not specified  **Comparison group care:** UC | **Diet :** NR  **PA:** 2.5 hrs of weekly workhours were allocated to mandatory PA on 2d. PA was medium to high intensity at 55-89% maximum heart rate (vigorous). Participants were allowed to choose any activity.  **Behavioral:** NR | **BMI:** y  **FPG:** y  **A1C:** y  **Insulin:** n  **Cholesterol:** y  **LDL**: y  **HDL:** y  **TG:** y  **SBP:** y  **DBP:** y  **Other:** Prolactin, waist-hip circumference | **Sampling method**: Recruited from community  **Jadad Score:** 1, 0, 1, C  **Randomization procedure:** NR  **Allocation** **concealment:** Bad  **Attrition (%):** 9.2  **Blinding pt:** No  **Blinding assessor:** No  **Blinding provider:** No  **BL comparable:** ND | |
| Wantanabe et al. 2003 | **Follow-up:** 12m  **No. study arms:** 2  **Setting:** Tokyo, Japan  **Number:** 173 | **Age (y):** 55.1  **Sex** (%F): 0  **Health condition**: high risk  **BL wt, BMI:** 24.4 kg/m^2^  **BL FPG**: 5.8 mmol/L  **BL A1C:** NR  **BL insulin:** NR | **Duration:** 12m  **Frequency:** 1/yr  **No. of contacts:** 2  **Group/individual:** Both  **Medium:** In-person  **Facilitator:** NR  **Comparison group care:** Conventional dietary reduction | **Diet:** Control portions; assess intake, glucose control and individual dietary counseling, learning to read menus based upon individual RD  **PA:** NR  **Behavioral:** Increase motivation to improve dietary practice | **BMI:** y  **FPG:** y  **A1C:** n  **Insulin:** n  **Cholesterol:** y  **LDL:** n  **HDL:** y  **TG:** y  **SBP:** y  **DBP:** y  **Other:** Liver function | **Sampling method:** Recruited from clinics  **Jadad Score:** 1, 0, 0, C  **Randomization procedure:** NR  **Allocation** **concealment:** Unclear  **Attrition (%):** 9.8  **Blinding pt:** Yes  **Blinding assessor:** No  **Blinding provider:** No  **BL comparable:** ND | |
| Weinstock et al.1998 | **Follow-up:** 23 m  **No. study arms:** 3  **Setting:** Clinic  **Number:** 45 | **Age (y):** 43.3  **Sex** (%F): 100  **Health condition:** Obese women  **BL wt, BMI:** 35.9 kg/m^2^  **BL FPG**: 5.1 mmol/L  **BL A1C:** NR  **BL insulin:** 15.4 µU/ml | **Duration:** 23 m  **Frequency:**  1^st^ 28w– weekly sessions. W29-W48 biweekly group sessions. Every 3m thereafter  **No. of contacts:** NR  **Group/individual:** Group  **Medium:** In-person  **Facilitator:** Psychologist/ dietitian  **Comparison group care:** Diet only | **Diet:** Liquid diet (925kcal/d) with a dinner entrée and a salad. Each serving of the liquid provided 150 kcal. After week 18 diet went up to 1250kcal/d  **PA:** Supervised training sessions (first 28w), 2 workouts/w (w28-49) and unsupervised thereafter.  **Behavioral:** NR | **BMI:** y  **FPG:** y  **A1C:** n  **Insulin:** y  **Cholesterol:** n  **LDL:** n  **HDL:** n  **TG:** n  **SBP:** y  **DBP:** y  **Other:**  NR | **Sampling method**: Subsample of a larger study  **Jadad Score:** 1,0,0, C  **Randomization procedure:** NR  **Allocation** **concealment:** Bad  **Attrition (%):** 0  **Blinding pt:** No  **Blinding assessor:** No  **Blinding provider:** No  **BL comparable:** ND | |
| Weiss et al. 2006 | **Follow-up:** 12m  **No. study arms:** 3  **Setting:** Clinic  **Number:** 48 | **Age (y):** 56.8  **Sex** (%F): 63.2  **Health condition:** Obese sedentary men and women  **BL wt, BMI:** 27.3 kg/m^2^  **BL FPG**: 5.3 mmol/L  **BL A1C:** NR  **BL insulin:** 7.8 µU/ml | **Duration:** 12 m  **Frequency:**  1/3m  **No. contacts:** 5  **Group/individual:** Both  **Medium:** In-person  **Facilitator:** Dietitian, exercise technician  **Comparison group care:** UC | **Diet:** Decrease calorie intake. Substitute foods with a low calorie density with those with a high calorie density.  **PA:** Participants exercised either in the facility or on their own.  **Behavioral:** Offered advice for eating a healthy diet and yoga classes | **BMI:** y  **FPG:** y  **A1C:** n  **Insulin**: y  **Cholesterol:** n  **LDL:** n  **HDL:** n  **TG:** n  **SBP:** n  **DBP:** n  **Other:** OGTT, VO2 max, fat mass | **Sampling method**: Recruited from community  **Jadad Score:** 1,0,1, C  **Randomization procedure:** NR  **Allocation** **concealment:** Bad  **Attrition (%):** 4.2  **Blinding pt:** No  **Blinding assessor:** No  **Blinding provider:** No  **Blinding provider:** No  **BL comparable:** ND | |
| Wing et al. 1995 | **Follow-up:** 18m  **No. study arms:** 2  **Setting:** Clinic  **Number:** 202 | **Age (y):** 37.4  **Sex** (%F): 48.1  **Health condition:** Obese adults  **BL wt, BMI:** 30.9 kg/m^2^  **BL FPG**: 5.5 mmol/L  **BL A1C:** NR  **BL insulin:** 27.1 µU/ml | **Duration:** 18m  **Frequency:**  0, 6, 12, 18m  **No. contacts:** 4  **Group/individual:** Individual  **Medium:** In-person  **Facilitator:** NR  **Comparison group care:** UC | **Diet:** 1000 or 1500 cal/d diets based on the initial weight. The cal intake was gradually increased if they achieved weight-loss goal 14, 18 or 24 kg.  **PA:** Exercise in the form of walking or cycling gradually increasing from 250cal/w to 1000 cal/w  **Behavioral:** NR | **BMI:** y  **FPG:** y  **A1C:** n  **Insulin:** n  **Cholesterol:** y  **LDL:** y  **HDL:** y  **TG:** y  **SBP:** y  **DBP:** y  **Other:** Waist/hip circumference | **Sampling method**: Recruited by ads  **Jadad Score:** 1,0,0,C  **Randomization procedure:** NR  **Allocation** **concealment:** Bad  **Attrition (%):** 21.3  **Blinding pt:** No  **Blinding assessor:** No  **Blinding provider:** No  **BL comparable:** ND | |
| Wing et al. 1998. | **Follow-up:** 24m  **No. study arms:** 4  **Setting:** Pittsburgh, PA  **Number:** 154 | **Age (y):** 45.7  **Sex** (%F): 79  **Health condition**: Overweight adults  **BL wt, BMI:** 35.9 kg/m^2^  **BL FPG**: 5.9 mmol/L  **BL A1C:** 7.2%  **BL insulin:** 15.9 µU/ml | **Duration:** 24m  **Frequency:** Two group sessions/w for four weeks; then monthly sessions  **No. of contacts:** 31  **Group/individual:** Group  **Medium:** In-person  **Facilitator:** NR  **Comparison group care:** UC | **Diet:** 800-1000kcal/d diet, with 20% cal as fat (weeks 1-8), then adjust 1200-1500kcal/d diet; self monitored cal intake  **PA:** 50-60 min walk with gradually increase PA to 1500 kcal/w through brisk walking 5d/w (3 miles each day)  **Behavioral:** Stimulus control, assertion, behavior chain analysis, problem solving and relapse prevention | **BMI:** y  **FPG:** y  **A1C:** y  **Insulin:** y  **Cholesterol:** y  **LDL:** y  **HDL:** y  **TG:** y  **SBP:** y  **DBP:** y  **Other:** WHR | **Sampling method:** Recruited by ads  **Jadad Score:** 1,0,0, C  **Randomization procedure:** NR  **Allocation** **concealment:** Unclear  **Attrition (%):** 22.0  **Blinding pt:** No  **Blinding assessor:** No  **Blinding provider:** No  **BL comparable:** ND | |
| Wycherley et al. 2012 | **Follow-up:** 12m  **No. study arms:** 2  **Setting:** Clinic, Australia  **Number:** 123 | **Age (y):** 50.8  **Sex** (%F): 0  **Health condition**: Overweight or obese adults  **BL wt:** 103.7 kg  **BMI:** 33.0 kg/m^2^  **BL FPG**: 5.8 mmol/L  **BL A1C:** NR  **BL insulin:** 10.0 µU/ml | **Duration:** 12m  **Frequency:** 3/12m  **No. of contacts:** 3  **Group/individual:** Group  **Medium:** In-person  **Facilitator:** NR  **Comparison group care:** High carbs diet | **Diet:** Isocaloric and moderate energy restricted diet, high protein (HP): 35% protein; 40% carbs; 25% fat.  **PA:**  **Behavioral:** | **BMI:** y  **FPG:** y  **A1C:** n  **Insulin:** y  **Cholesterol:** y  **LDL:** y  **HDL:** y  **TG:** y  **SBP:** y  **DBP:** y  **Other:** NR | **Sampling method:** Recruited by public ads  **Jadad Score: 2**,0,1, C  **Randomization procedure:** Computer-generated number  **Allocation** **concealment:** NR  **Attrition (%):** 44.7  **Blinding pt:** No  **Blinding assessor:** No  **Blinding provider:** No  **BL comparable:** ND | |
| Yeh et al. 2016 | **Follow-up:** 12m  **No. study arms:** 2  **Setting:** Community, New York  **Number:** 60 | **Age (y):** 58.9  **Sex** (%F): 56.7  **Health condition**: PreDM  **BL wt:** 67.5 kg  **BMI:** 26.1 kg/m^2^  **BL FPG**: 5.9 mmol/L  **BL A1C:** 6.1%  **BL insulin:** NR | **Duration:** 12m  **Frequency:** 1/2w for 6m, 1/m for 6m  **No. of contacts:** 18  **Group/individual:** Group  **Medium:** In-person  **Facilitator:** Physicians, community leaders  **Comparison group care:** UC | **Diet:** Modified DPP model focusing on healthy eating, rice bowls for portion control.  **PA:** Increase PA  **Behavioral:** Stress reduction and problem-solving skill | **BMI:** y  **FPG:** y  **A1C:** y  **Insulin:** n  **Cholesterol:** y  **LDL:** y  **HDL:** y  **TG:** y  **SBP:** y  **DBP:** y  **Other:** WC | **Sampling method:** Recruited from hospital record  **Jadad Score:** 1,0,1, C  **Randomization procedure:** NR  **Allocation** **concealment:** NR  **Attrition (%):** 3.3  **Blinding pt:** No  **Blinding assessor:** No  **Blinding provider:** No  **BL comparable:** ND | |

Abbreviations: A1C: glycated hemoglobin; BL: baseline; BMI: body mass index; cal: calorie; carbs: carbohydrates; CG: control group; CV: cardiovascular; CVD: cardiovascular disease; DBP: diastolic blood pressure; FPG: fasting plasma glucose; hr: hour; HDL: high density lipoprotein cholesterol; HOMA-IR: homeostasis model assessment insulin resistance; HT: hypertension; HRR: heart rate reserve; IG: intervention group; kcal: kilocalorie; kg: kilogram; LCD: low caloric diet; LDL: low density lipoprotein cholesterol; m: month; MetS: metabolic syndrome; min: minute; MJ: megajoule; MUFA: monounsaturated fatty acid; n: no; ND: no difference; NR: not reported; OGTT: oral glucose tolerance test; PA: physical activity; pt: patient; PUFA: polyunsaturated fatty acid; RD: registered dietarian; SBP: systolic blood pressure; TG: triglyceride; UC: usual care; VLCD: very low caloric diet; VO2max: maximal oxygen uptake; w: week; WC: waist circumference; WHR: waist-to-hip ratio; wt: weight; y: yes; yr: year;

| **Table C in S1 File. Lifestyle Interventional Effect: Meta-Analyses Results in Single Arm Models** | | | | | | | | | | | | | | |  | |  | |  |
| --- | --- | --- | --- | --- | --- | --- | --- | --- | --- | --- | --- | --- | --- | --- | --- | --- | --- | --- | --- |
|  | SBP (mmHg) | | |  | |  | | | DBP (mmHg) | |  | |  | | TC (mmol/L) | |  | |  |
|  | Study arms (sample size) | | | Pooled effect mean (95%CI) | | Hetero- geneity p value | | | Study arms (sample size) | | Pooled effect mean (95%CI) | | Hetero- geneity p value | | Study arms (sample size) | | Pooled effect mean (95%CI) | | Hetero- geneity p value |
| VLCD | NA | | |  | | |  | | NA | |  | |  | | 2 (54) | | -0.11 (-0.32, 0.10) | | 0.3 |
| LCD | 15 (529) | | | -4.14 (-7.10, -1.18) | | <0.01 | | | 15 (527) | | -3.90 (-5.45, -2.36) | | <0.01 | | 24 (829) | | -0.17 (-0.28, -0.07) | | <0.01 |
| LCD+PA | 5 (240) | | | 2.02 (-3.26, 7.31) | | <0.01 | | | 5 (240) | | 0.74 (-0.88, 2.37) | | 0.6 | | 8 (311) | | -0.23 (-0.42, -0.04) | | <0.01 |
| PA | 5 (281) | | | -4.06 (-8.17, 0.05) | | <0.01 | | | NA | |  | |  | | 8 (258) | | -0.08 (-0.35, 0.19) | | <0.01 |
|  |  | | |  | |  | | |  | |  | |  | |  | |  | |  |
| **Table C in S1 File. Lifestyle Interventional Effect: Meta-Analyses Results in Single Arm Models (cont.)** | | | | | | | | | | | | | | | | |  | |  |
|  | LDL-C (mmol/L) | | | | |  | | | HDL-C (mmol/L) | | | |  | | TG (mmol/L) | |  | |  |
|  | Study arms (sample size) | | | Pooled effect mean (95%CI) | | Hetero- geneity p value | | | Study arms (sample size) | | Pooled effect mean (95%CI) | | Hetero- geneity p value | | Study arms (sample size) | | Pooled effect mean (95%CI) | | Hetero- geneity p value |
| VLCD | NA | | |  | |  | | | NA | |  | |  | | 2 (54) | | -0.58 (-0.83, -0.32) | | 0.4 |
| LCD | 24 (807) | | | -0.25 (-0.34, -0.16) | | <0.01 | | | 24 (795) | | 0.12 (0.09. 0.15) | | <0.01 | | 22 (770) | | -0.34 (-0.40, -0.28) | | <0.01 |
| LCD+PA | 3 (71) | | | -0.46 (-0.86, 0.06) | | <0.01 | | | 5 (107) | | 0.04 (-0.02, 0.10) | | 0.5 | | 2 (61) | | -0.27 (-0.44, -0.09) | | 0.07 |
| PA | 6 (236) | | | -0.15 (-0.40, 0.11) | | <0.01 | | | 10 (429) | | 0.04 (0.01, 0.06) | | 0.12 | | 10 (579) | | -0.13 (-0.24, -0.02) | | <0.01 |
| Abbreviations: CI: confidence interval; DBP: diastolic blood pressure; HDL-C: high density lipoprotein cholesterol; LCD: low calorie | | | | | | | | | | | | | | | | | | | |
| diet; LDL-C: low density lipoprotein cholesterol; PA: physical activity; SBP: systolic blood pressure; TC: total cholesterol; | | | | | | | | | | | | | | | | | | | |
| TG: triglycerides; VLCD: very low calorie diet | | | | | | | | | | |  | |  | |  | |  | |  |
| **Table D in S1 File. Lifestyle Interventional Effect on Glucose Regulation and Weight Loss: Meta-Analyses Results** | | | | | | | | | | | | | | | | | |  |  |
|  | |  | FPG (mmol/L) | |  | | |  | |  | | Weight Loss (%) | |  | |  | |  |  |
|  | | Studies (sample size) | Pooled effect mean (95%CI) | | Effect size | | | Heterogeneity p value | | Studies (sample size) | | Pooled effect mean (95%CI) | | Effect size | | Heterogeneity p value | |  |  |
| LI vs UC (all studies*) | | 55 (9234) | -0.14 (-0.19, -0.10) | | 0.06 | | | <0.01 | | 49 (8728) | | -3.99 (-4.69, -3.29) | | 0.12 | | <0.01 | |  |  |
| LI vs UC (all studies^§^) | | 63 (10033) | -0.12 (-0.17, -0.08) | | 0.05 | | | <0.01 | | 57 (9347) | | -3.81 (-4.45, -3.17) | | 0.12 | | <0.01 | |  |  |
|  | |  |  | |  | | |  | |  | |  | |  | |  | |  |  |
| LI vs UC (Group 1^ƚ^) | | 24 (4383) | -0.09 (-0.13, -0.05) | | 0.07 | | | <0.01 | | 19 (3285) | | -3.63 (-4.75, -2.52) | | 0.11 | | <0.01 | |  |  |
| LI vs UC (Group 2^ǂ^) | | 31 (4941) | -0.18 (-0.25, -0.11) | | 0.07 | | | <0.01 | | 30 (5443) | | -4.20 (-5.14, -3.27) | | 0.12 | | <0.01 | |  |  |
|  | |  |  | |  | | |  | |  | |  | |  | |  | |  |  |
| LI vs UC (F/U=12 mo)* | | 46 (7626) | -0.11 (-0.15, -0.07) | | 0.06 | | | <0.01 | | 41 (7188) | | -3.68 (-4.50, -2.87) | | 0.10 | | <0.01 | |  |  |
| LI vs UC (13-23 mo)* | | 8 (1560) | -0.15 (-0.21, -0.09) | | 0.12 | | | 0.91 | | 6 (1289) | | -3.28 (-4.39, -2.17) | | 0.16 | | 0.09 | |  |  |
| LI vs UC (≥24 mo)* | | 15 (3423) | -0.12 (-0.23, -0.01) | | 0.04 | | | <0.01 | | 15 (3424) | | -3.58 (-4.98, -2.19) | | 0.09 | | <0.01 | |  |  |
|  | |  |  | |  | | |  | |  | |  | |  | |  | |  |  |
| PA vs UC | | 14 (1813) | -0.07 (-0.11, -0.03) | | 0.08 | | | 0.74 | | 12 (1663) | | -1.55 (-2.53, -0.57) | | 0.08 | | 0.38 | |  |  |
| D vs UC | | 7 (499) | -0.17 (-0.27, -0.08) | | 0.15 | | | 0.46 | | 6 (433) | | -6.21 (-8.63, -3.19) | | 0.24 | | <0.01 | |  |  |
| PA+D vs UC | | 34 (7012) | -0.15 (-0.21, -0.09) | | 0.06 | | | <0.01 | | 31 (6632) | | -4.12 (-4.93, -3.30) | | 0.12 | | <0.01 | |  |  |
| Abbreviations: CI: confidence interval; D: diet; FPG: fasting plasma glucose; LI: lifestyle intervention; mo: month; | | | | | | | | | | | | | | | | | |  |  |
| PA: physical activity; UC: usual care; vs: versus | | | | | | | | | |  | |  | |  | |  | |  |  |
| * All studies with attrition <30% | | | | |  | | |  | |  | |  | |  | |  | |  |  |
| § All studies with attrition <30% plus studies with attrition ≥30% | | | | | | | | | | | |  | |  | |  | |  |  |
| ƚ All studies with attrition <30% and participants with FPG<5.5 mmol/L or A1C <5.5% | | | | | | | | | | | | | |  | |  | |  |  |
| ǂ All studies with attrition <30% and participants with FPG≥5.5 mmol/L or A1C≥5.5% | | | | | | | | | | | | | |  | |  | |  |  |
